# Supplementary figures and images for: Multi-task snake optimization algorithm for global optimization and planar kinematic arm control problem
Source: PeerJ Comput Sci. 2025 Feb 11;11:e2688. doi: 10.7717/peerj-cs.2688 (PMC11888922; doi:10.7717/peerj-cs.2688)

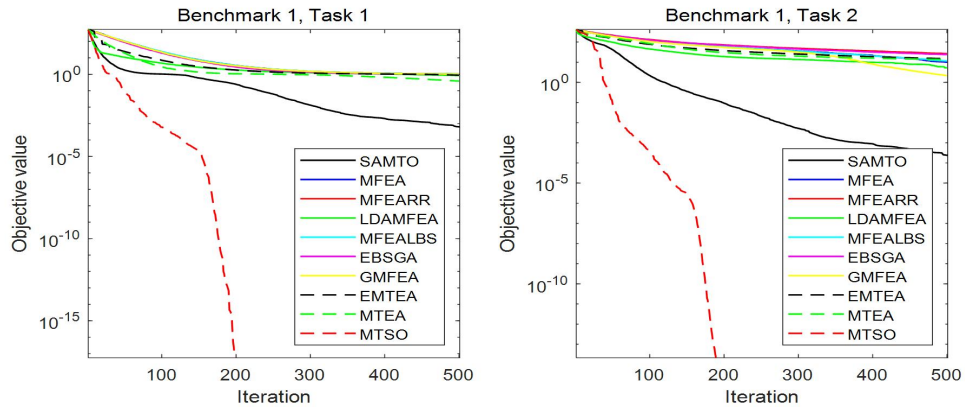

(a)

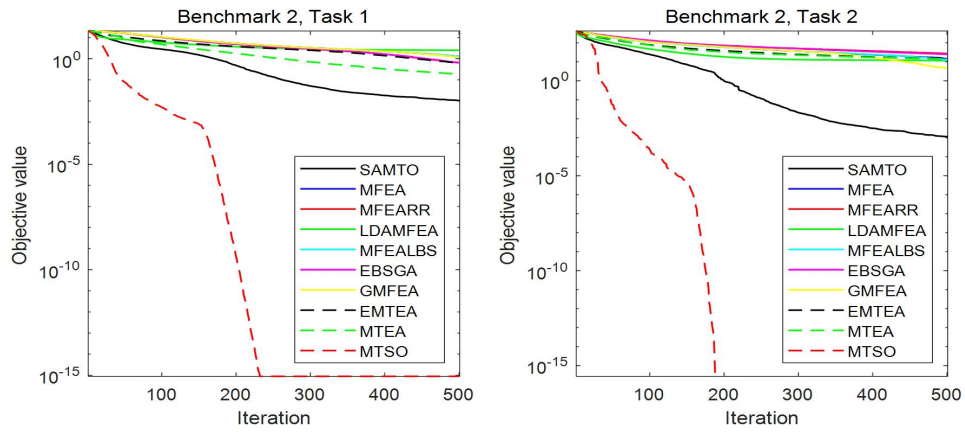

(b)

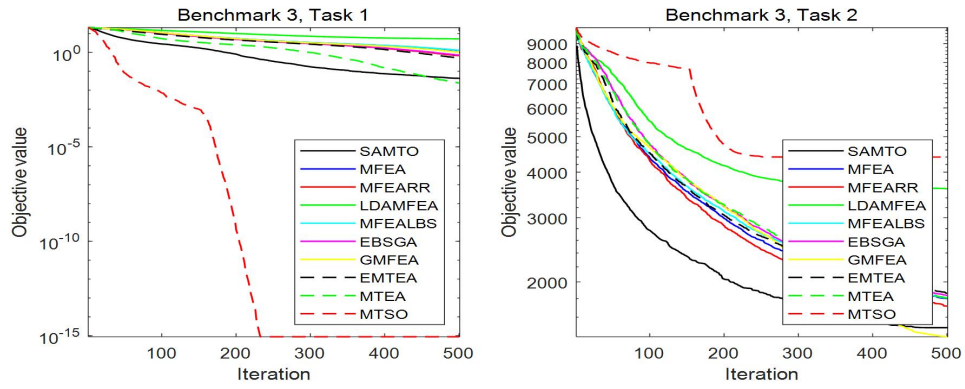

(c)

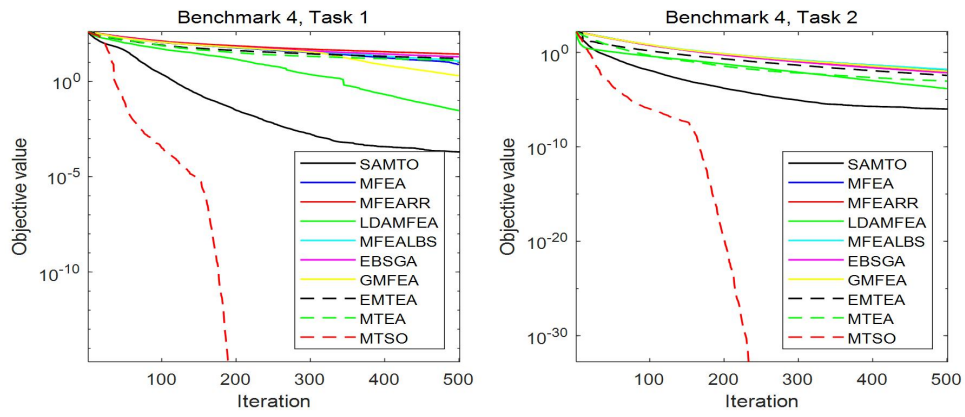

(d)

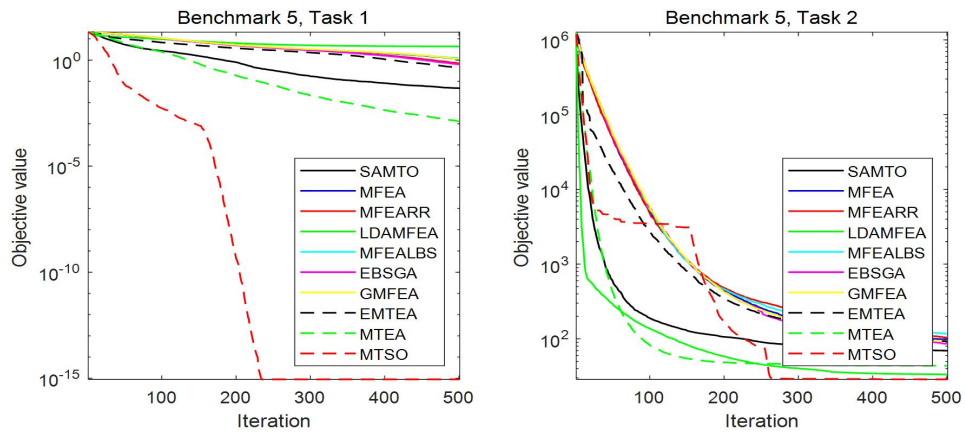

(e)

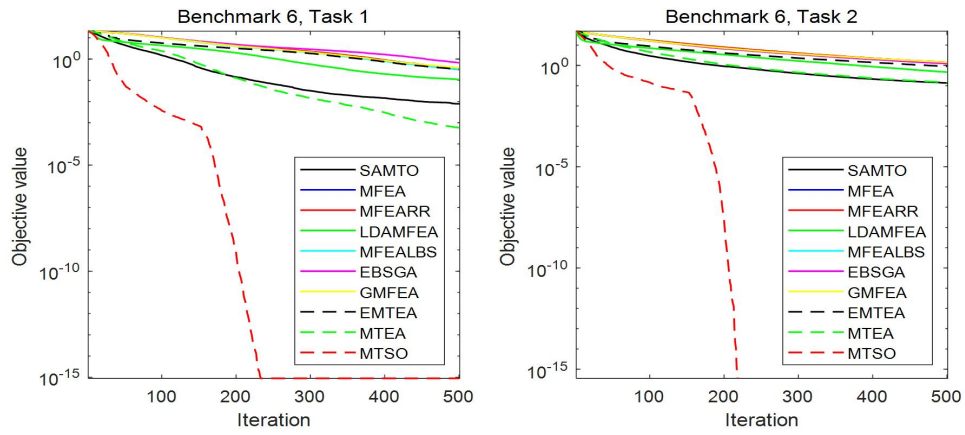

(f)

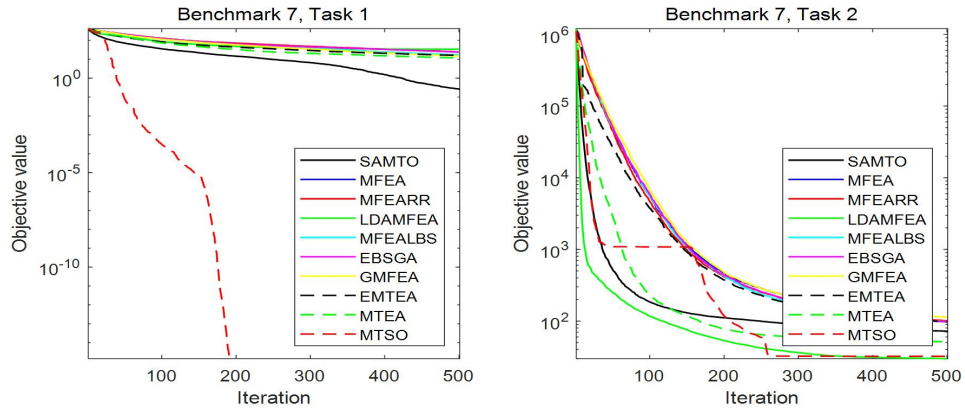

(g)

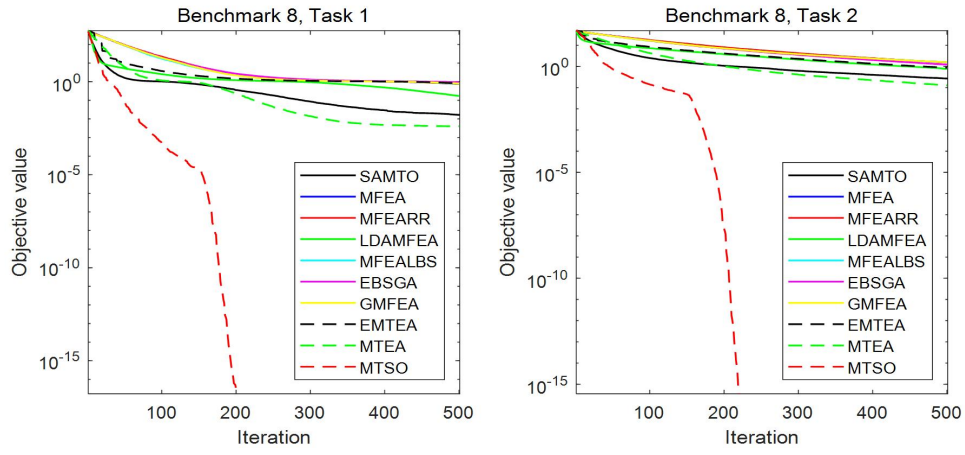

(h)

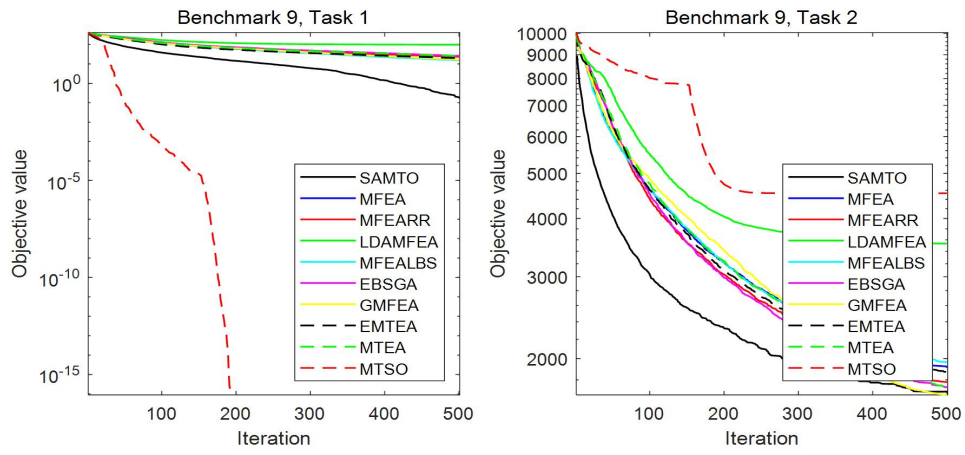

(i)

Supplement: Supplemental Information 1 [file peerj-cs-11-2688-s001.pdf]

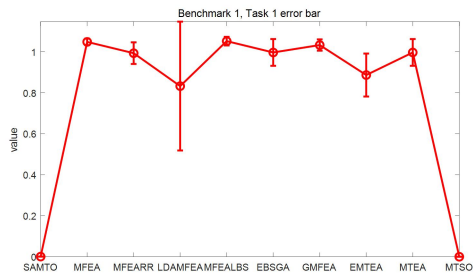

(1a)

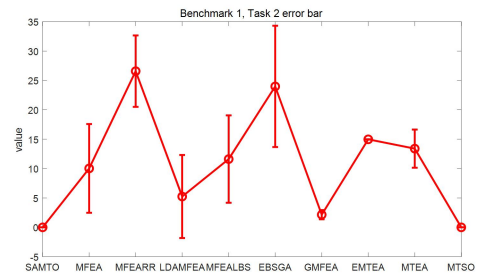

(1b)

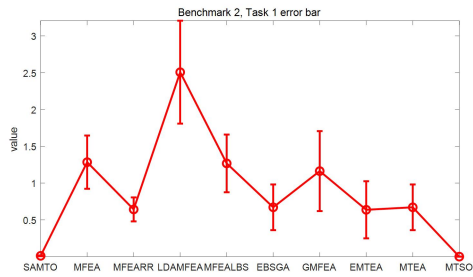

(2a)

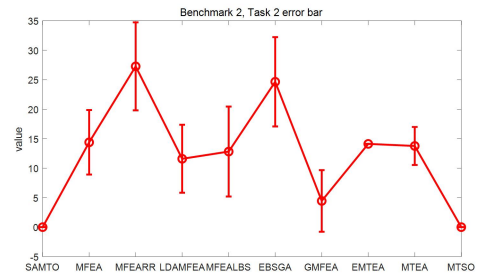

(2b)

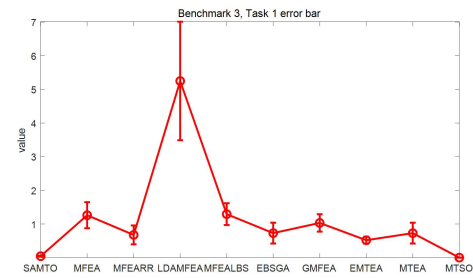

(3a)

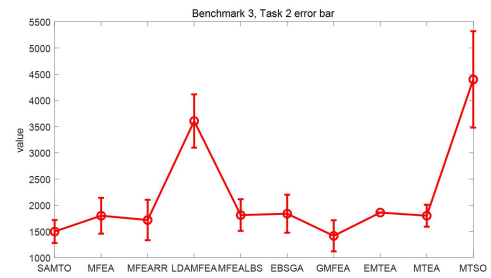

(3b)

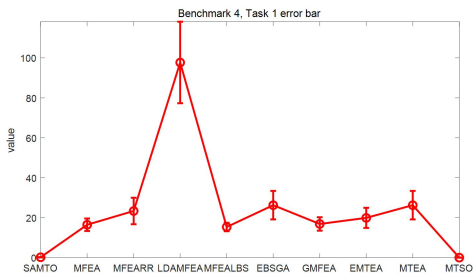

(4a)

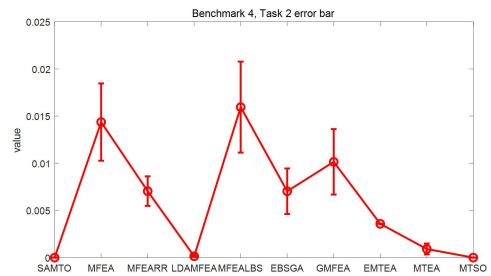

(4b)

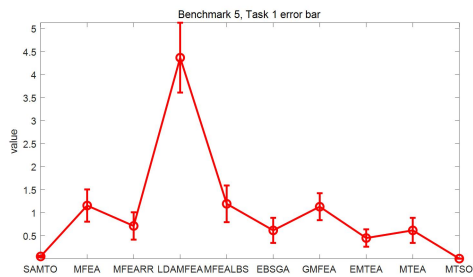

(5a)

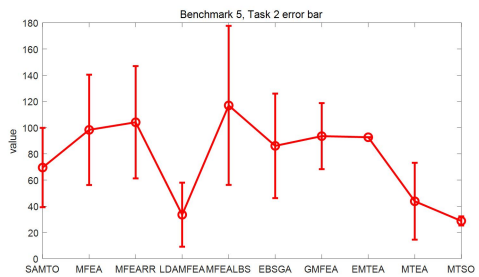

(5b)

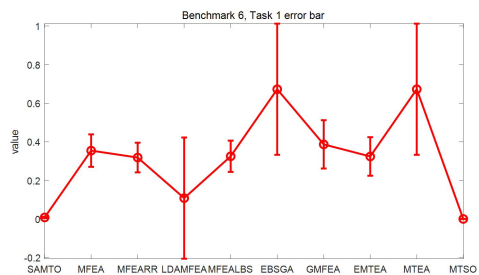

(6a)

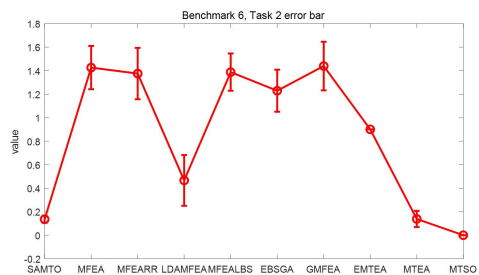

(6b)

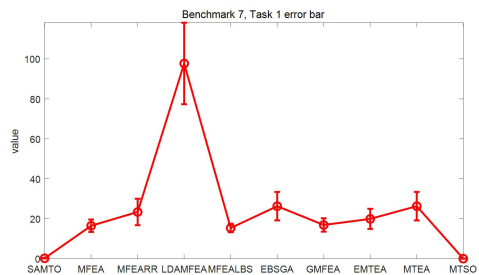

(7a)

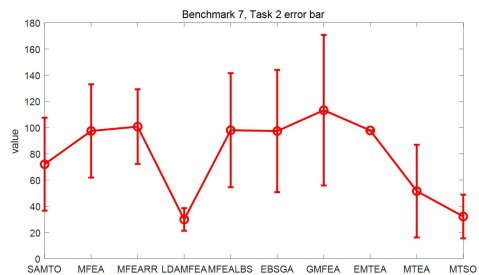

(7b)

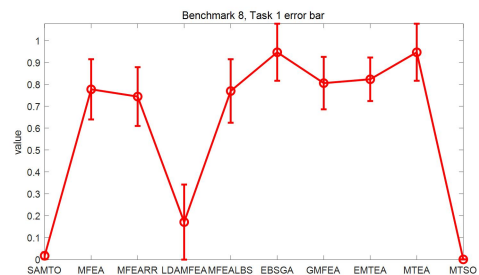

(8a)

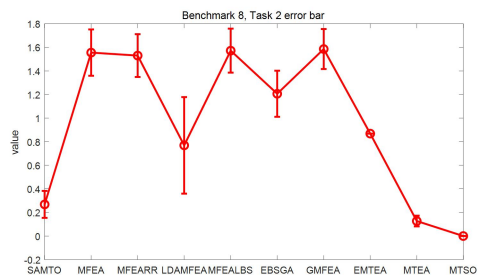

(8b)

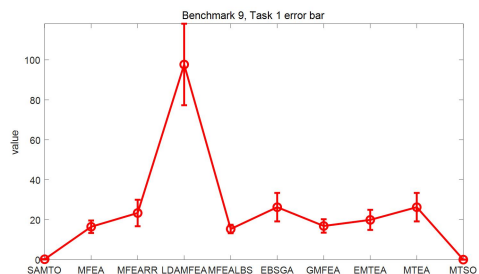

(9a)

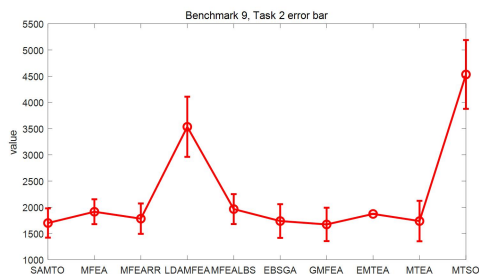

(9b)

Supplement: Supplemental Information 2 [file peerj-cs-11-2688-s002.pdf]

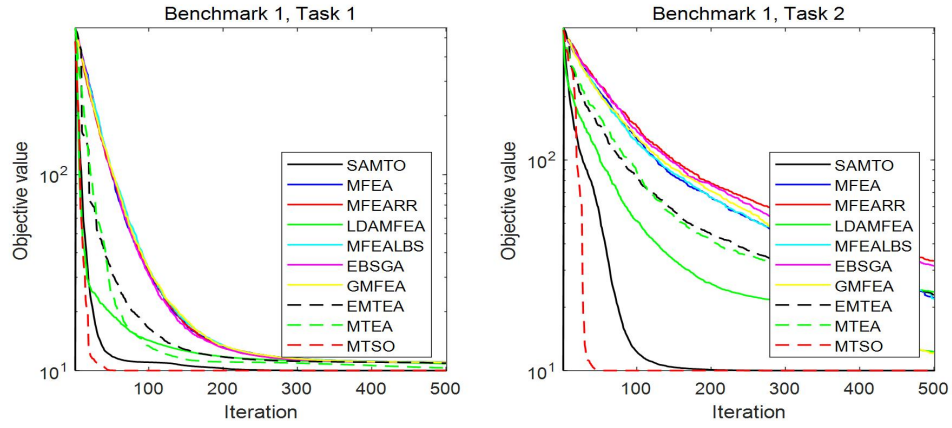

(a)

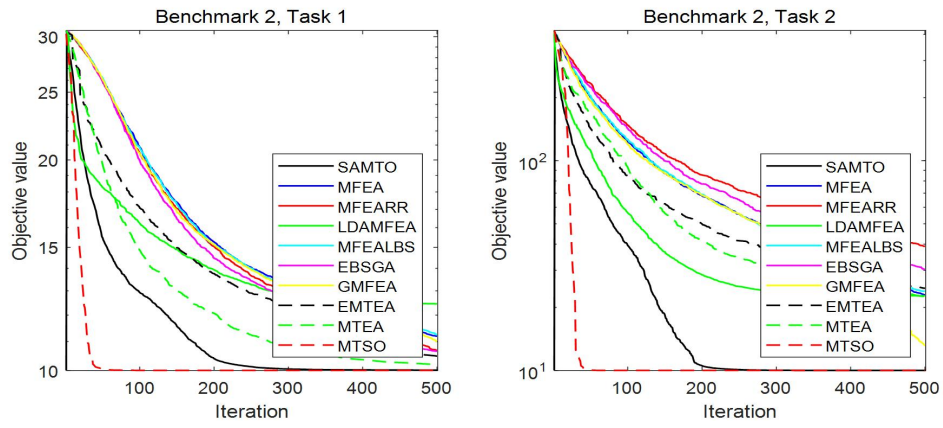

(b)

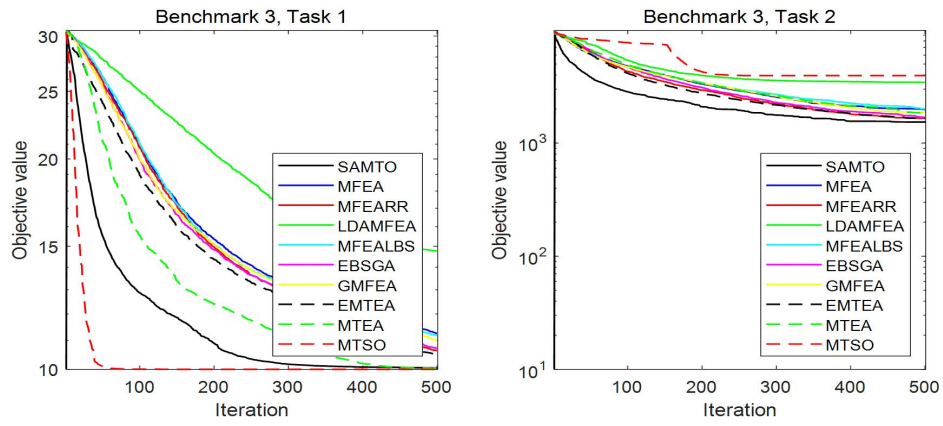

(c)

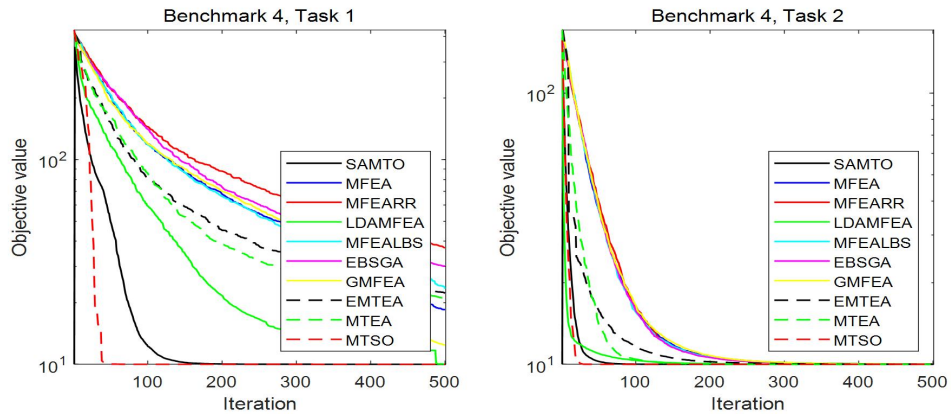

(d)

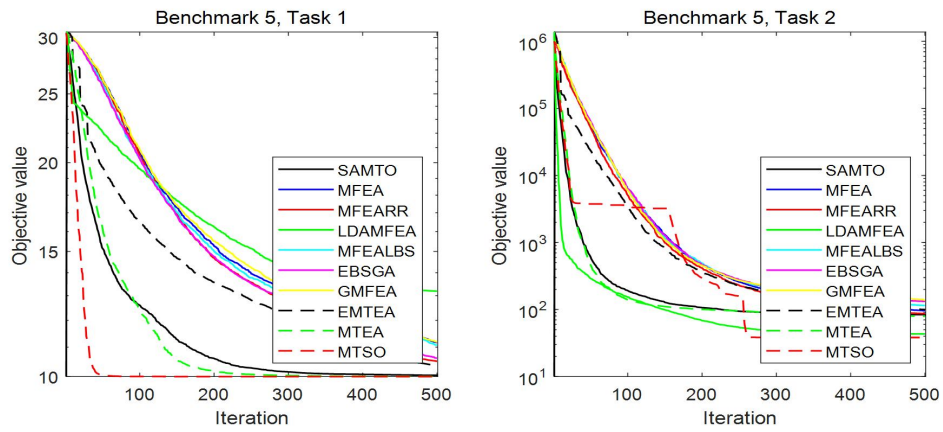

(e)

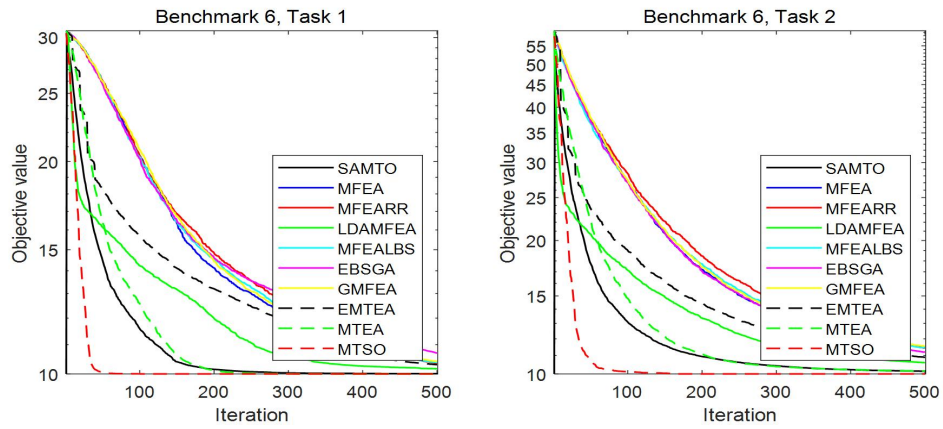

(f)

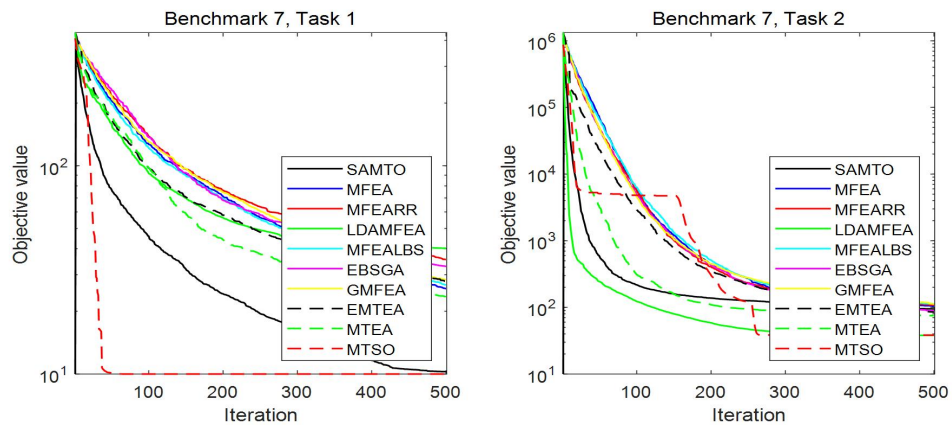

(g)

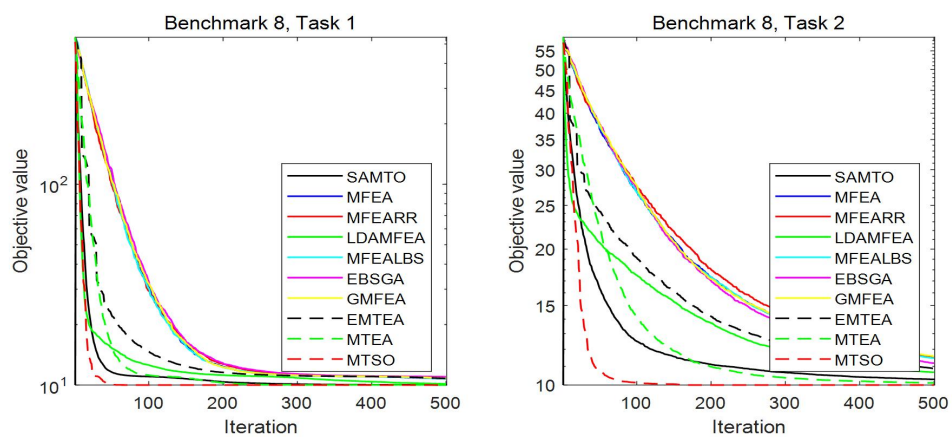

(h)

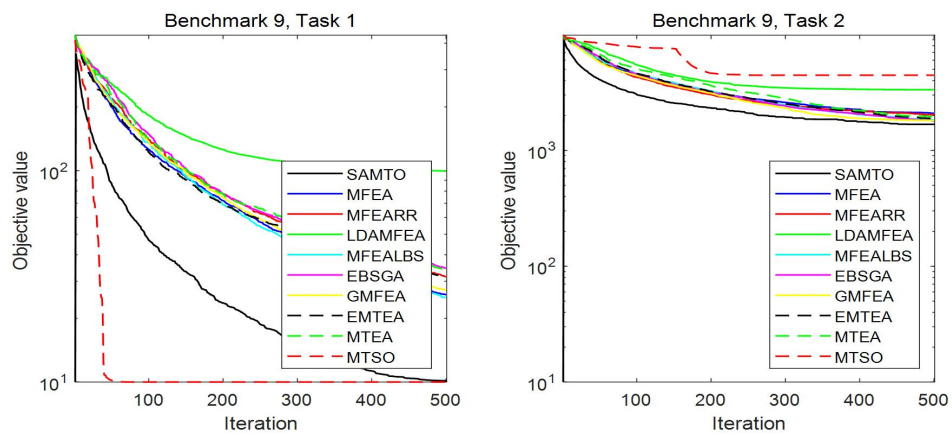

(i)

Supplement: Supplemental Information 3 [file peerj-cs-11-2688-s003.pdf]

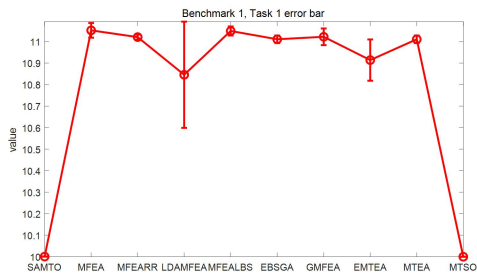

(1a)

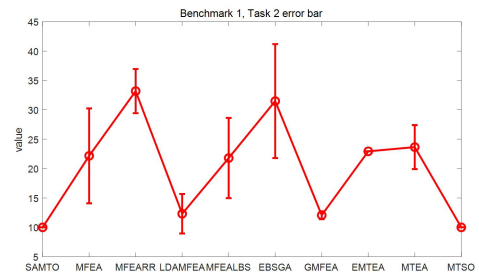

(1b)

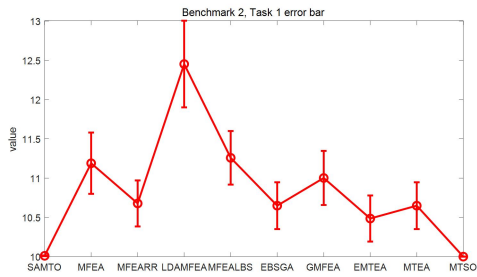

(2a)

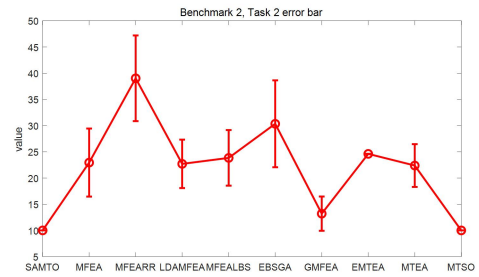

(2b)

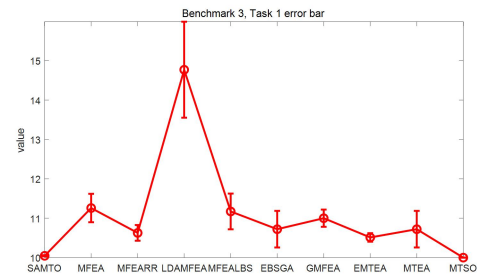

(3a)

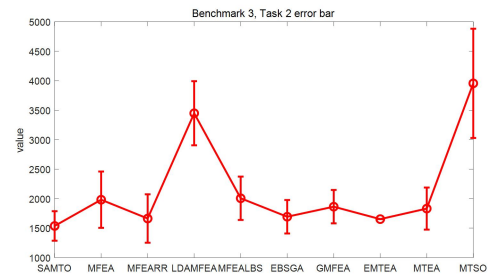

(3b)

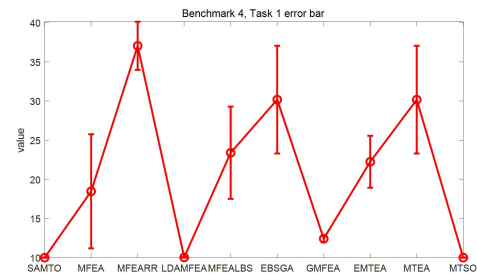

(4a)

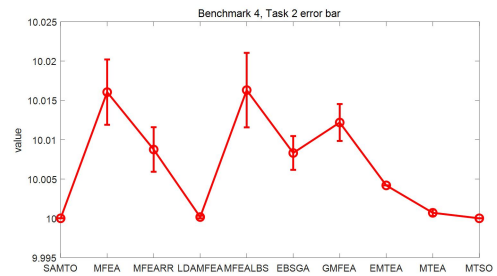

(4b)

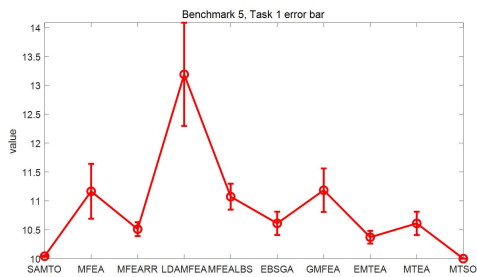

(5a)

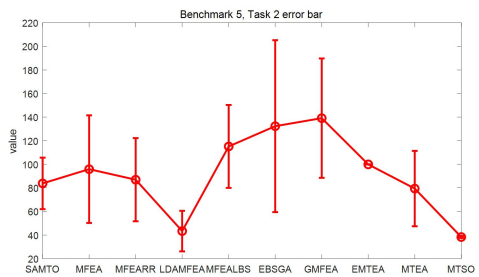

(5b)

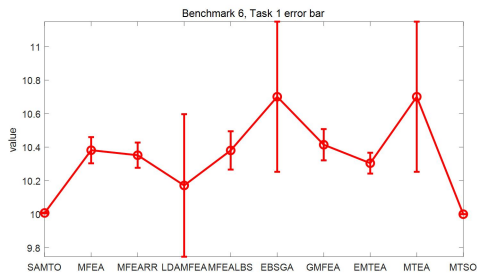

(6a)

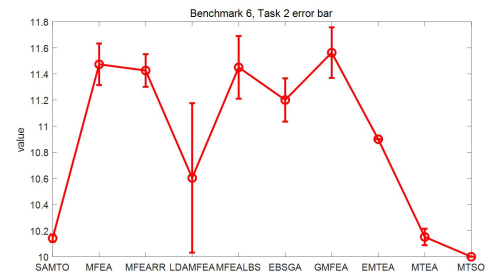

(6b)

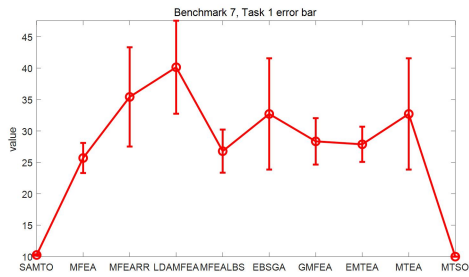

(7a)

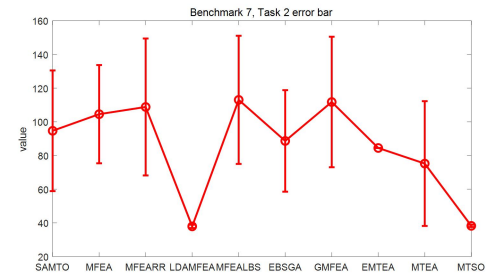

(7b)

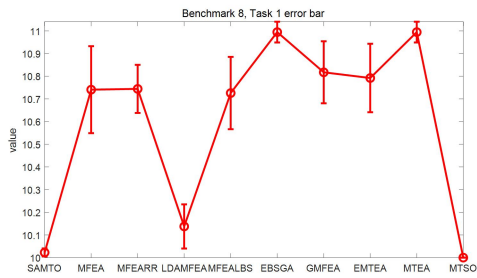

(8a)

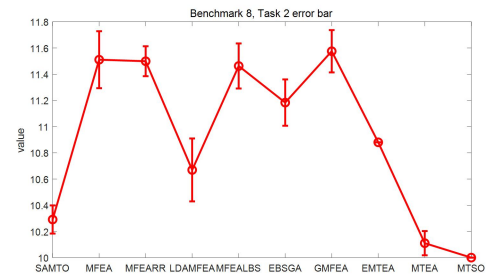

(8b)

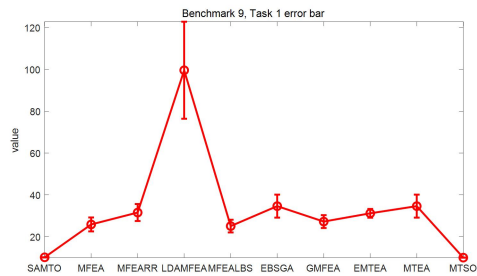

(9a)

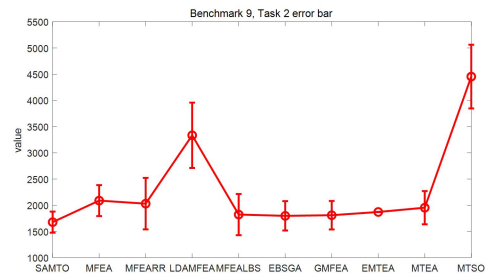

(9b)

Supplement: Supplemental Information 4 [file peerj-cs-11-2688-s004.pdf]

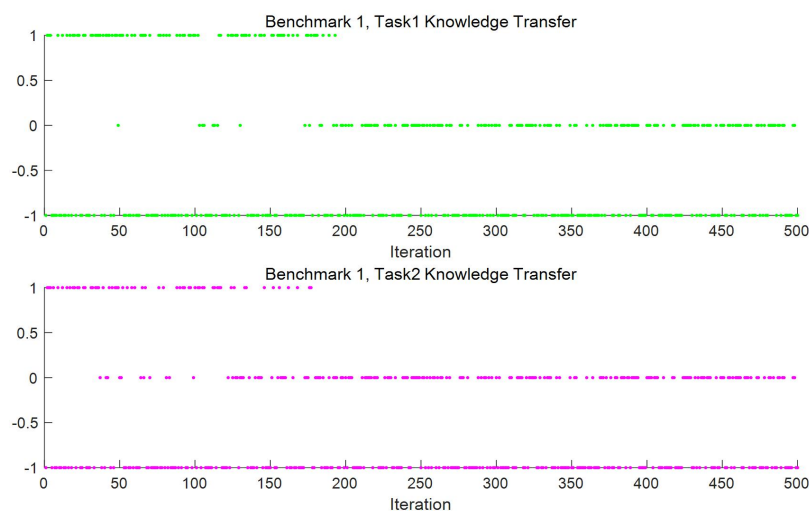

(1)

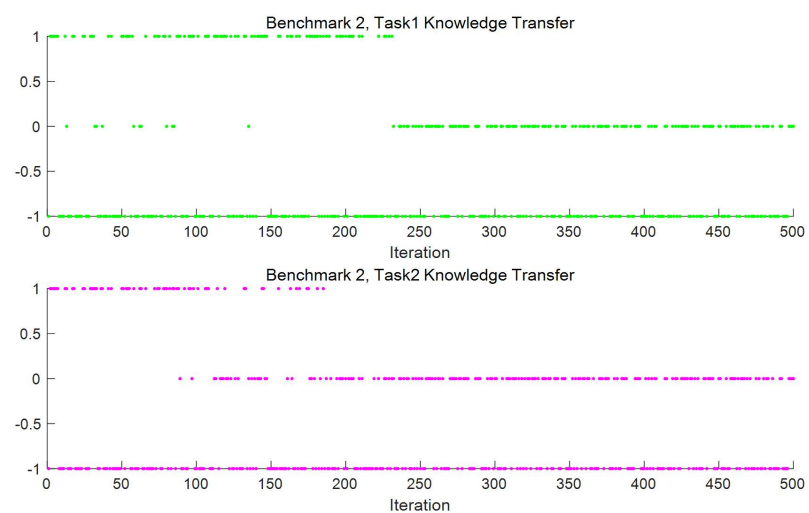

(2)

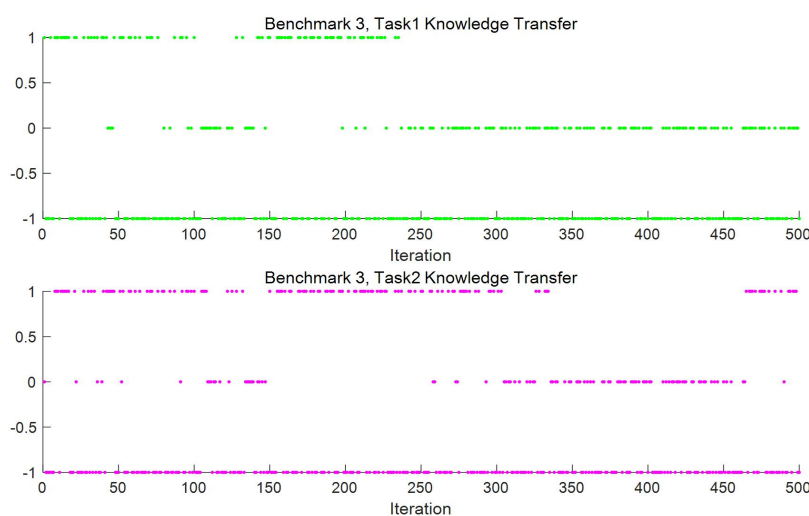

(3)

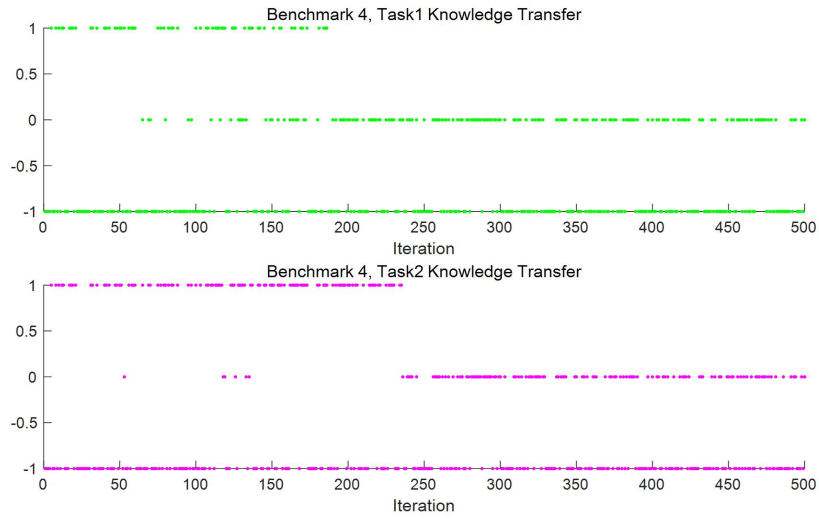

(4)

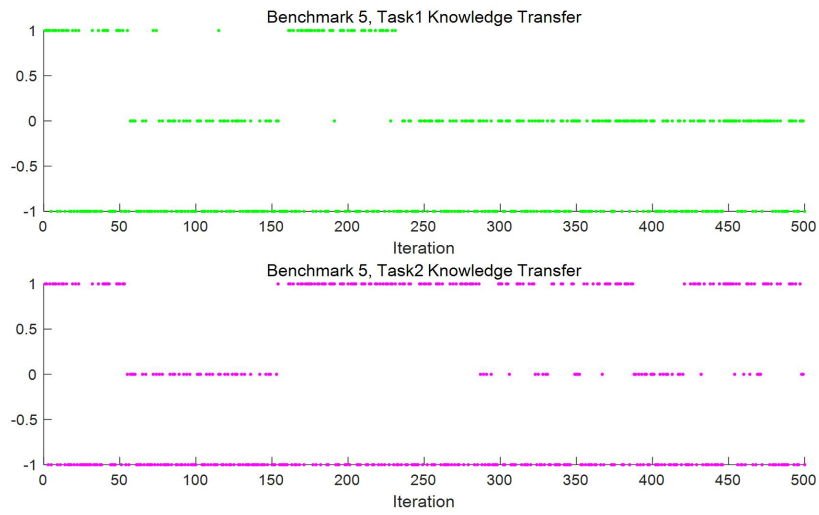

(5)

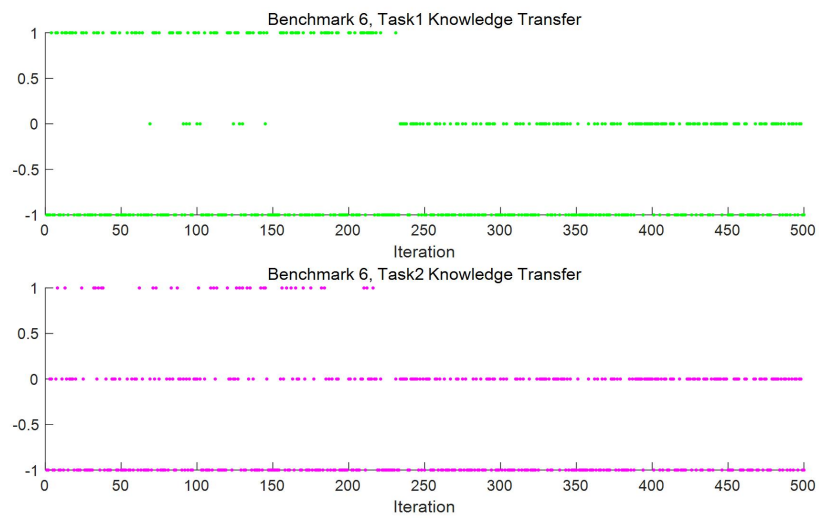

(6)

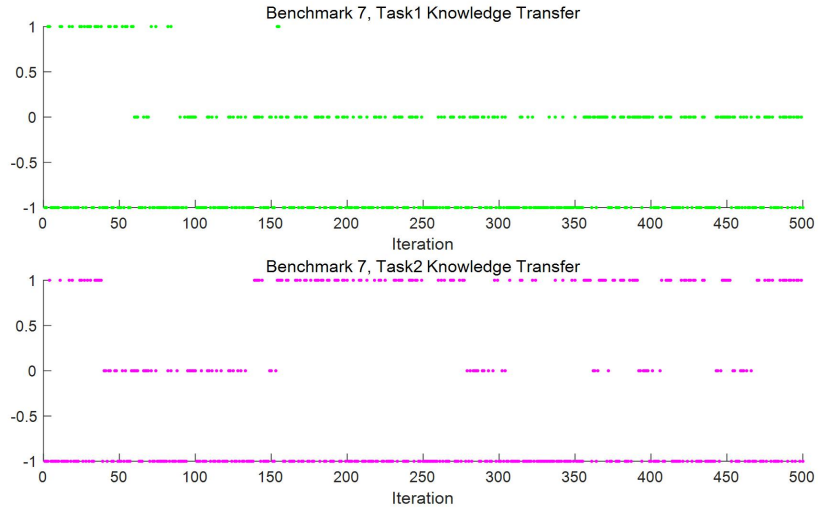

(7)

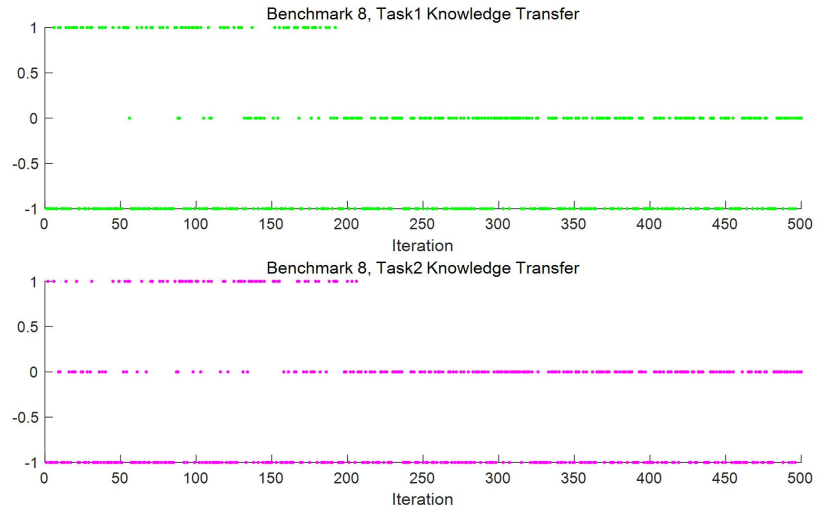

(8)

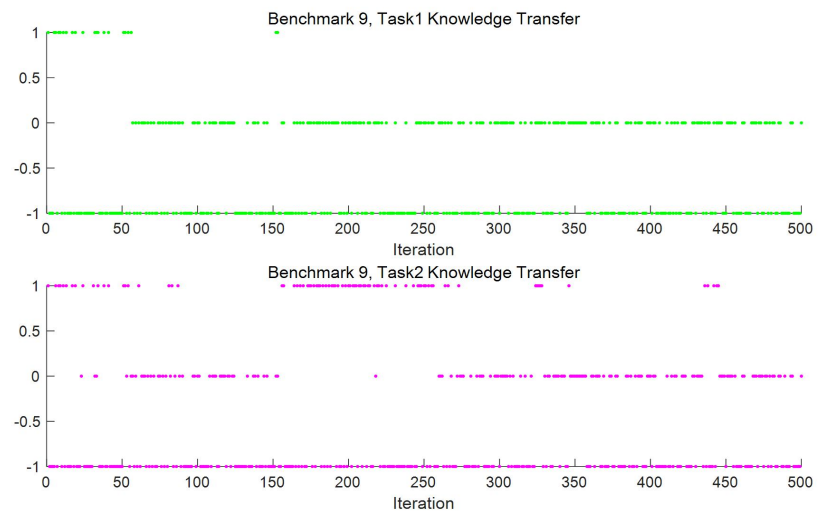

(9)

Supplement: Supplemental Information 5 [file peerj-cs-11-2688-s005.pdf]

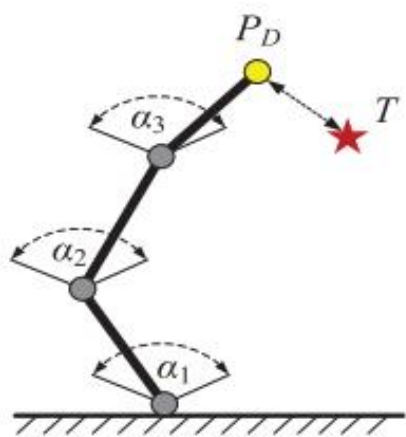

(a)

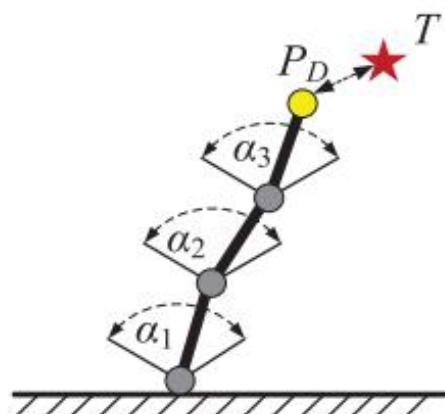

(b)

Supplement: Supplemental Information 6 — Task 1 (a), Task 2 (b). [file peerj-cs-11-2688-s006.pdf]

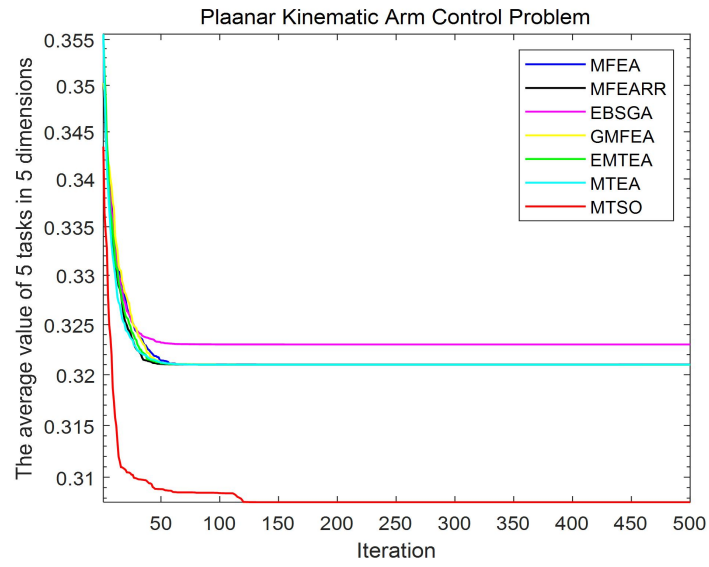

(a)

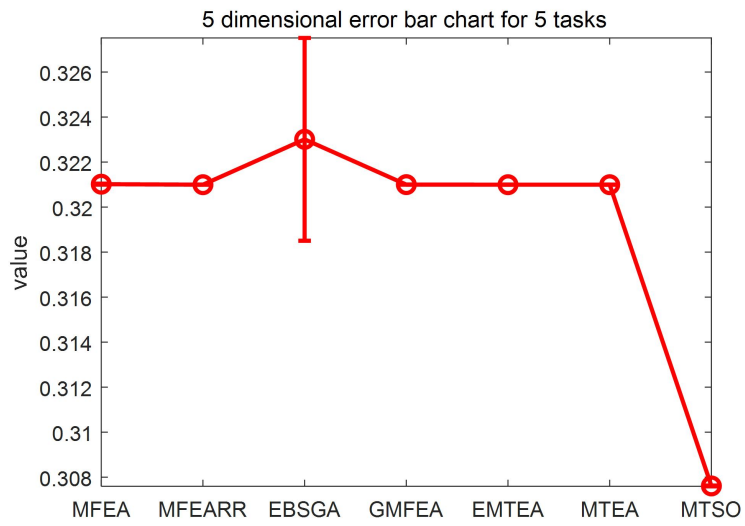

(b)

Supplement: Supplemental Information 7 [file peerj-cs-11-2688-s007.pdf]

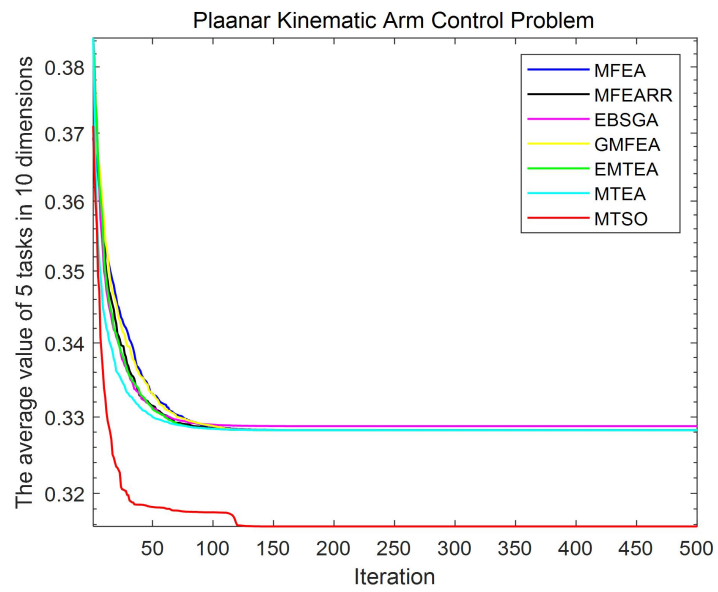

(a)

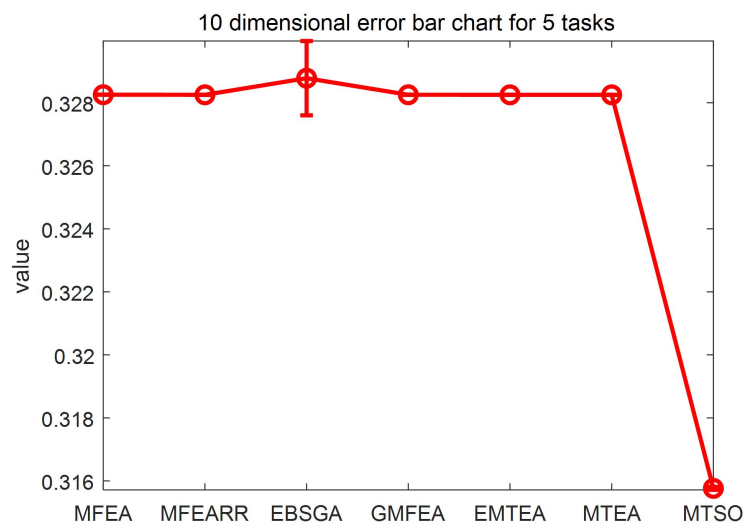

(b)

Supplement: Supplemental Information 8 [file peerj-cs-11-2688-s008.pdf]

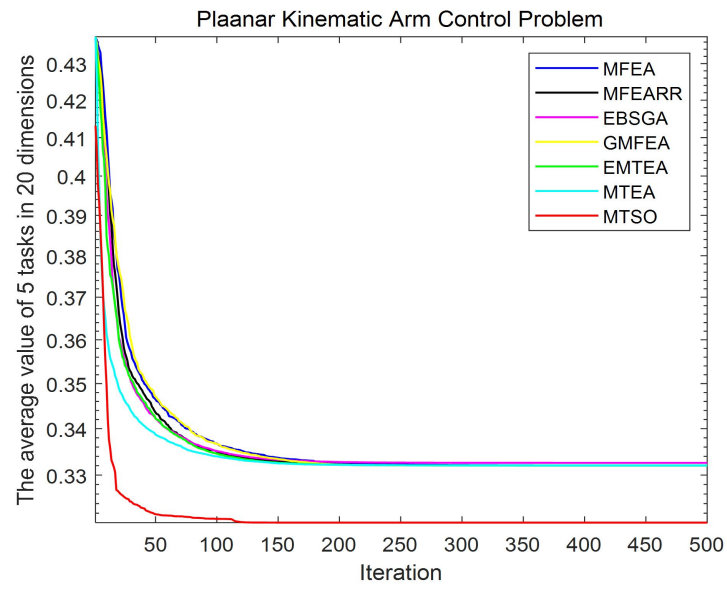

(a)

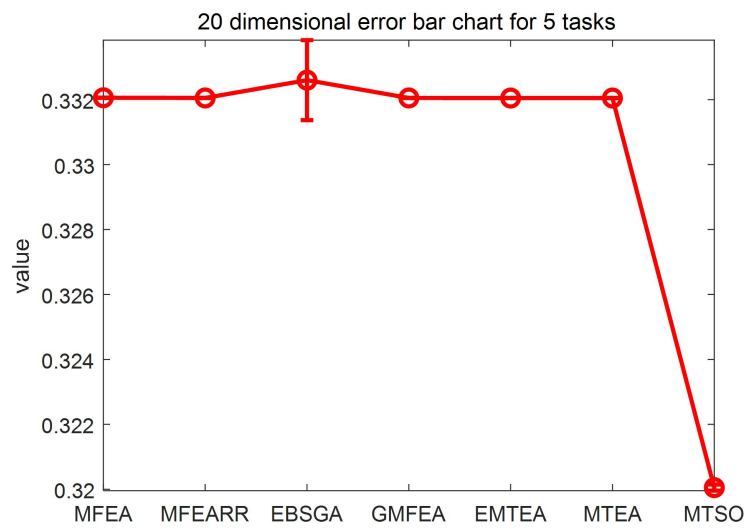

(b)

Supplement: Supplemental Information 9 [file peerj-cs-11-2688-s009.pdf]

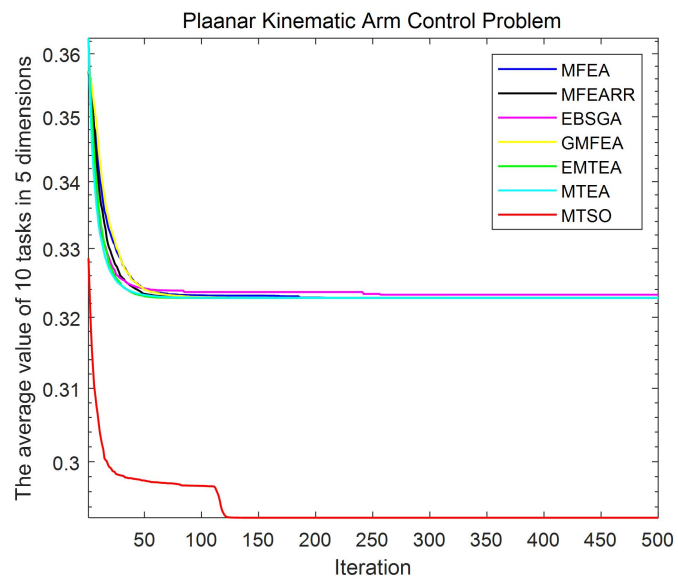

(a)

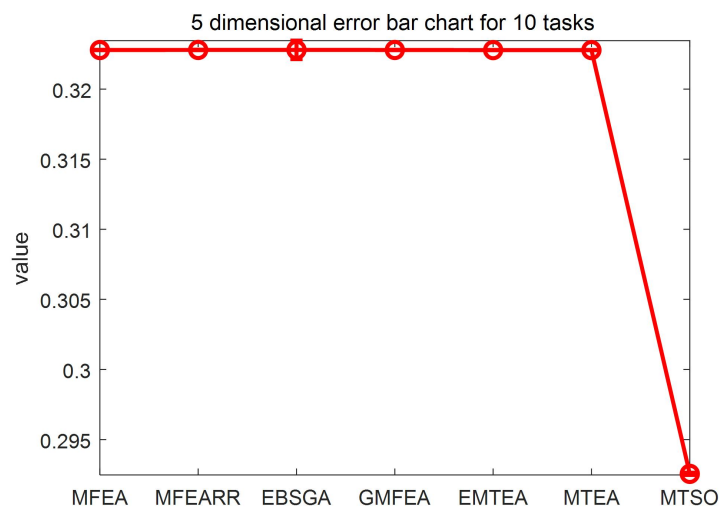

(b)

Supplement: Supplemental Information 10 [file peerj-cs-11-2688-s010.pdf]

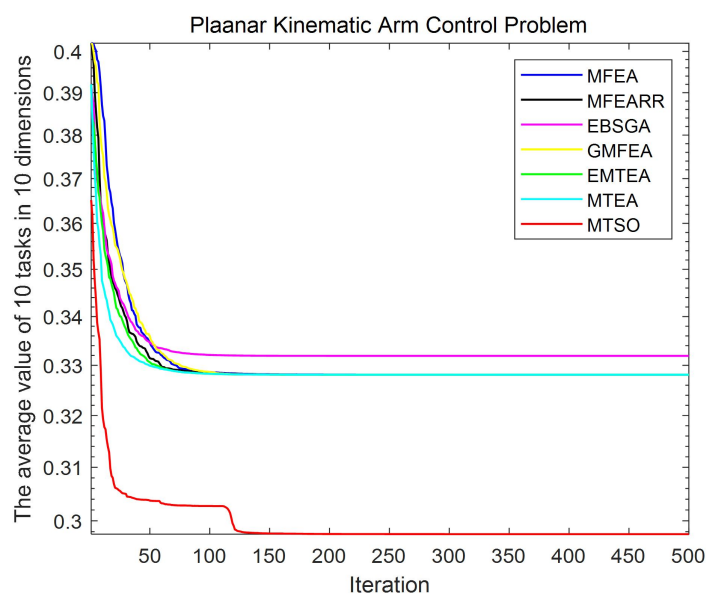

(a)

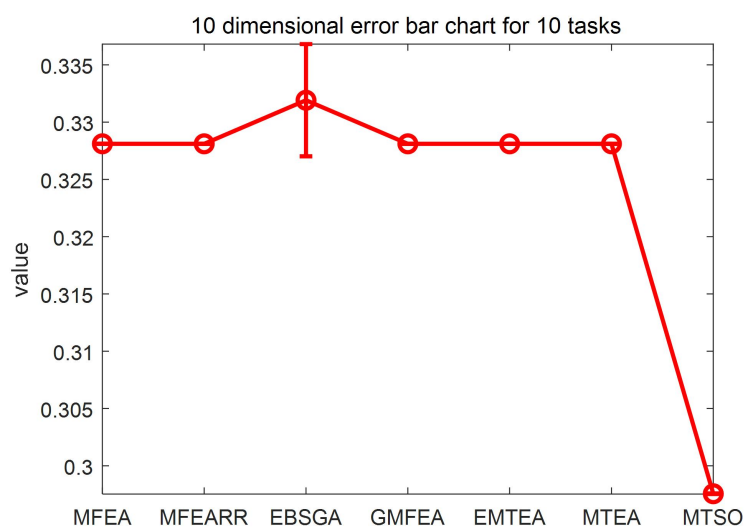

(b)

Supplement: Supplemental Information 11 [file peerj-cs-11-2688-s011.pdf]

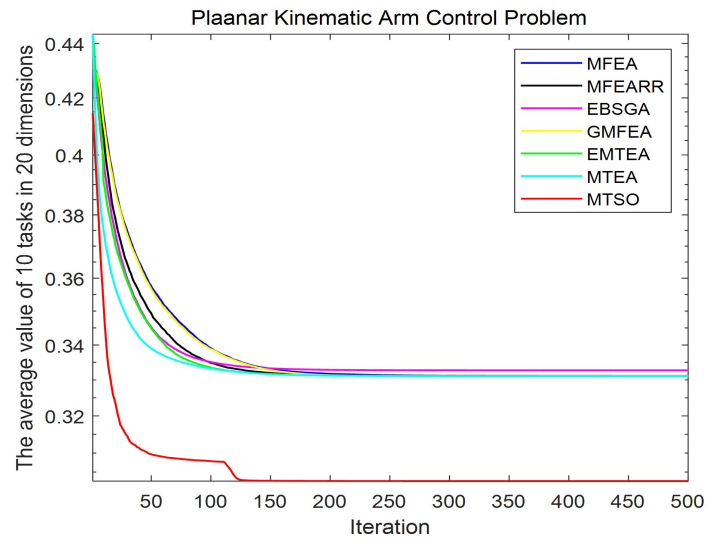

(a)

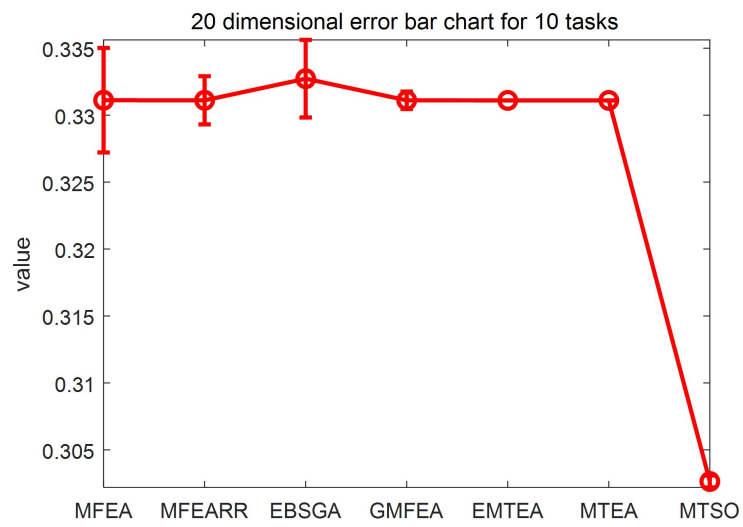

(b)

Supplement: Supplemental Information 12 [file peerj-cs-11-2688-s012.pdf]

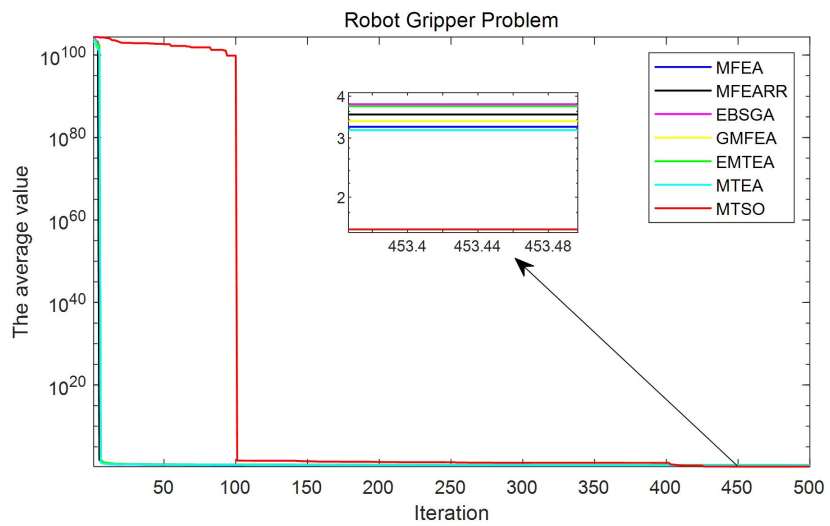

(a)

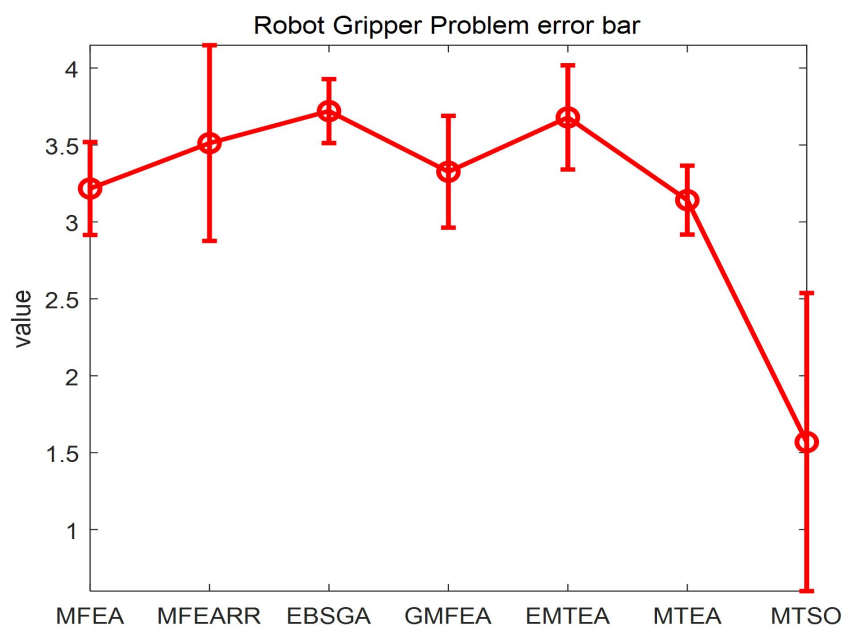

(b)

Supplement: Supplemental Information 13 [file peerj-cs-11-2688-s013.pdf]

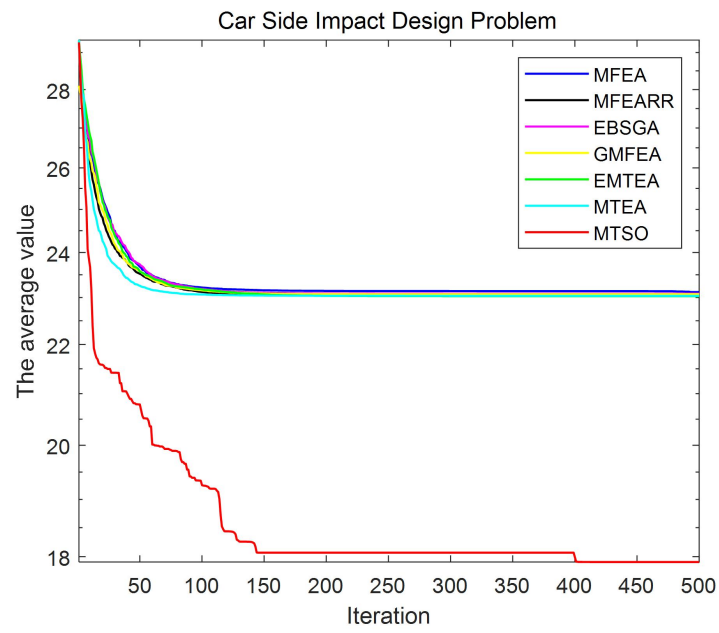

(a)

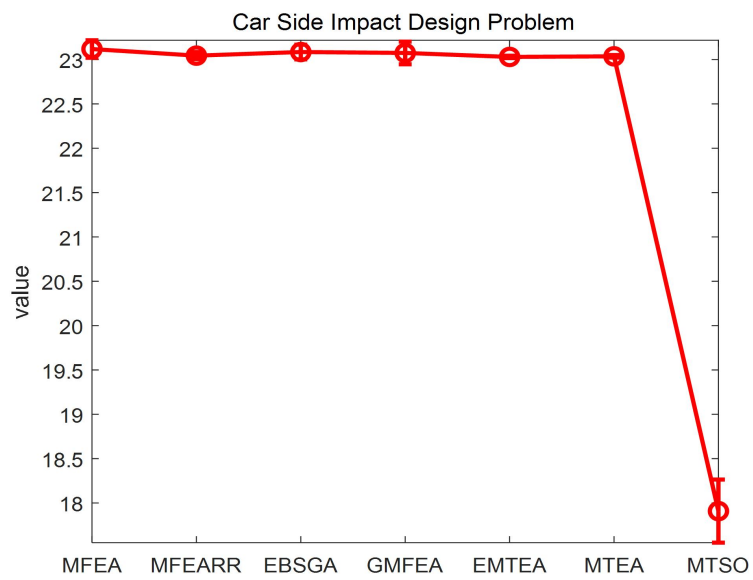

(b)

Supplement: Supplemental Information 14 [file peerj-cs-11-2688-s014.pdf]
