# Supplementary material for: Multi-task snake optimization algorithm for global optimization and planar kinematic arm control problem
Source: PeerJ Comput Sci. 2025 Feb 11;11:e2688. doi: 10.7717/peerj-cs.2688 (PMC11888922; doi:10.7717/peerj-cs.2688)
Supplement: Supplemental Information 15 [file peerj-cs-11-2688-s015.doc]

| **Symbol** | **Meaning** |
| --- | --- |
| MTO | The Multitask Optimization |
| MTSO | Multitask Snake Optimization Algorithm |
| SO | Snake Optimization Algorithm |
| SI | swarm intelligence |
| KT | Knowledge transfer |
| MFEA | Multi-Factor Evolutionary Algorithm |
| Std | The standard deviation |
| PKACP | Planar Kinematic Arm Control Problem |
