# Supplementary material for: Multi-task snake optimization algorithm for global optimization and planar kinematic arm control problem
Source: PeerJ Comput Sci. 2025 Feb 11;11:e2688. doi: 10.7717/peerj-cs.2688 (PMC11888922; doi:10.7717/peerj-cs.2688)
Supplement: Supplemental Information 16 [file peerj-cs-11-2688-s016.doc]

| **Algorithm 2** Multitask Snake Optimization Algorithm |
| --- |
| 1: Set Algorithm Parameters (Tasks,*N*, *T*,RMP,R1,nRepeat,ntasks) |
| 2: Population initialization |
| 3: **While** (*t* ≤ *T_max_* ) **do** |
| 4: Using the Snake Optimization Algorithm to individually optimize and solve n tasks.  5： Selecting the top fifth of individuals for each task as elite individuals. |
| 6: **While** ( k≤no_of_tasks) **do** |
| 7: Generate random numbers r1 and r2. |
| 8: **If** (*r1<RMP*) **then**  9: **If** (*r2<R1*) **then**  10: Randomly transferring knowledge from the elite repository to non-task-specific individuals. |
| 11: **Else**  12： Random perturbation applied to the worst-performing individuals in this task.  13： **End If**  14： **Else**  15： Reverse learning through lens imaging applied to all individuals in this task. |
| 16: **End If** |
| 17: **End While** |
| 18: **End While** |
| 19: Return to the best solution. |
