# Supplementary material for: Multi-task snake optimization algorithm for global optimization and planar kinematic arm control problem
Source: PeerJ Comput Sci. 2025 Feb 11;11:e2688. doi: 10.7717/peerj-cs.2688 (PMC11888922; doi:10.7717/peerj-cs.2688)
Supplement: Supplemental Information 17 [file peerj-cs-11-2688-s017.doc]

| **Number** | **Name** | **Global minimum** | **Dimension** | **Function Characteristics** |
| --- | --- | --- | --- | --- |
| 1 | Griewank | 0 | 30 | Multi-modal |
| 2 | Rastrigin | 0 | 30 | Multi-modal |
| 3 | Ackley | 0 | 30 | Multi-modal |
| 4 | Schwefel | 0 | 30 | Multi-modal |
| 5 | Sphere | 0 | 30 | Multi-modal |
| 6 | Weierstrass | 0 | 30 | Multi-modal |
| 7 | Rosenbrock | 0 | 30 | Multi-modal |
