# Supplementary material for: Multi-task snake optimization algorithm for global optimization and planar kinematic arm control problem
Source: PeerJ Comput Sci. 2025 Feb 11;11:e2688. doi: 10.7717/peerj-cs.2688 (PMC11888922; doi:10.7717/peerj-cs.2688)
Supplement: Supplemental Information 18 [file peerj-cs-11-2688-s018.doc]

| **Test Combinations** | |  | **SAMTO** | **MFEA** | **MFEARR** | **LDAMFEA** | **MFEALBS** | **EBSGA** | **GMFEA** | **EMTEA** | **MTEA** | **MTSO** |  |
| --- | --- | --- | --- | --- | --- | --- | --- | --- | --- | --- | --- | --- | --- |
| 1 | Task1 | Mean | 0.6312e-03 | 1.0477 | 0.9926 | 0.8321 | 1.0514 | 0.9961 | 1.0323 | 0.8860 | 0.3877 | **0** |  |
|  |  | Std | 0.5018e-03 | 0.0160 | 0.0527 | 0.3142 | 0.0212 | 0.0653 | 0.0276 | 0.1047 | 0.1400 | **0** |  |
|  |  | P-value | 1.8214e-07 | 8.0065e-09 | 8.0061e-09 | 8.0065e-09 | 8.0065e-09 | 8.0063e-09 | 8.0065e-09 | 8.0065e-09 | 8.0065e-09 | **-** |  |
|  | Task2 | Mean | 0.2372e-03 | 10.0278 | 26.5813 | 5.2459 | 11.6189 | 23.9840 | 2.1528 | 14.9788 | 13.3953 | **0** |  |
|  |  | Std | 0.2068e-03 | 7.5510 | 6.0821 | 7.0724 | 7.4337 | 10.3268 | 0.7868 | 3.7980 | 3.2456 | **0** |  |
|  |  | P-value | 1.8213e-07 | 8.0065e-09 | 8.0065e-09 | 8.0062e-09 | 8.0065e-09 | 8.0065e-09 | 8.0065e-09 | 2.1518e-05 | 8.0065e-09 | **-** |  |
|  | run time |  | 48.1177 | 27.6400 | 31.8536 | 43.9701 | 16.0716 | 349.477 | 17.4739 | 15.8550 | 16.9278 | **10.9489** |  |
| 2 | Task1 | Mean | 0.0106 | 1.2840 | 0.6417 | 2.5068 | 1.2674 | 0.6704 | 1.1626 | 0.6365 | 0.1848 | **0** |  |
|  |  | Std | 0.0051 | 0.3620 | 0.1626 | 0.6995 | 0.3921 | 0.3105 | 0.5437 | 0.3880 | 0.0737 | **0** |  |
|  |  | P-value | 1.8214e-07 | 8.0065e-09 | 8.0065e-09 | 8.0065e-09 | 8.0065e-09 | 8.0065e-09 | 8.0065e-09 | 8.0065e-09 | 8.0065e-09 | **-** |  |
|  | Task2 | Mean | 0.0011 | 14.3847 | 27.2652 | 11.5904 | 12.8151 | 24.6515 | 4.4426 | 14.1171 | 13.7643 | **0** |  |
|  |  | Std | 0.0012 | 5.4700 | 7.4646 | 5.7654 | 7.6266 | 7.5733 | 5.2226 | 3.0000 | 3.2217 | **0** |  |
|  |  | P-value | 1.8214e-07 | 8.0065e-09 | 8.0065e-09 | 8.0065e-09 | 8.0065e-09 | 8.0065e-09 | 8.0065e-09 | 2.1518e-05 | 8.0065e-09 | **-** |  |
|  | run time |  | 45.0181 | 23.0929 | 28.6530 | 40.4025 | 16.2880 | 17.7763 | 27.3497 | 15.3660 | 17.7276 | **10.4660** |  |
| 3 | Task1 | Mean | **0** | 1.3 | 0.7 | 5.2 | 1.3 | 0.7 | 1.0 | 0.5 | **0** | **0** |  |
|  |  | Std | 0.0144 | 0.3883 | 0.2806 | 1.7615 | 0.3247 | 0.3092 | 0.2580 | 0.1048 | 0.0203 | **0** |  |
|  |  | P-value | 1.8214e-07 | 8.0065e-09 | 8.0065e-09 | 8.0065e-09 | 8.0065e-09 | 8.0065e-09 | 8.0065e-09 | 8.0065e-09 | 8.0065e-09 | **-** |  |
|  | Task2 | Mean | 1.4979e+03 | 1.7993e+03 | 1.7172e+03 | 3.6059e+03 | 1.8113e+03 | 1.8374e+03 | **1.4147e+03** | 1.8591e+03 | 1.7998e+03 | 4.3995e+03 |  |
|  |  | Std | 220.3479 | 341.6261 | 385.8081 | 507.8107 | 302.3329 | 363.4312 | 296.8111 | 309.6063 | **208.9724** | 919.8908 |  |
|  |  | P-value | 1.2009e-05 | 6.7956e-08 | 6.7956e-08 | 0.0043 | 6.7956e-08 | 6.7956e-08 | 6.7956e-08 | 0.1167 | 6.7956e-08 | - |  |
|  | run time |  | 44.9512 | 27.6286 | 31.8203 | 43.8813 | 16.1299 | 17.5403 | 26.8410 | 15.8994 | 17.0137 | **11.0284** |  |
| 4 | Task1 | Mean | 0.1989e-03 | 7.7363 | 27.2852 | 0.0296 | 10.9225 | 19.4656 | 2.0076 | 16.0976 | 12.6099 | **0** |  |
|  |  | Std | 0.1659e-03 | 6.0853 | 8.7128 | 0.0164 | 7.0862 | 10.0420 | 0.7292 | 6.7000 | 4.3864 | **0** |  |
|  |  | P-value | 1.8214e-07 | 8.0065e-09 | 8.0065e-09 | 8.0065e-09 | 8.0065e-09 | 8.0065e-09 | 8.0065e-09 | 8.0065e-09 | 8.0065e-09 | **-** |  |
|  | Task2 | Mean | 0.0010 | 0.0144 | 0.0070 | 0.0001 | 0.0160 | 0.0070 | 0.0101 | 0.0036 | 0.0009 | **0** |  |
|  |  | Std | 0.0008e-03 | 0.0041 | 0.0016 | 0.0001 | 0.0048 | 0.0024 | 0.0035 | 0.0013 | 0.0006 | **0** |  |
|  |  | P-value | 1.8214e-07 | 8.0065e-09 | 8.0065e-09 | 8.0065e-09 | 8.0065e-09 | 8.0065e-09 | 8.0065e-09 | 2.1518e-05 | 8.0065e-09 | **-** |  |
|  | run time |  | 44.5827 | 24.3489 | 29.5979 | 43.5837 | 16.1211 | 17.4975 | 26.8535 | 15.8589 | 16.9460 | **10.8286** |  |
| 5 | Task1 | Mean | 0.0471 | 1.1535 | 0.7104 | 4.3679 | 1.1913 | 0.6138 | 1.1285 | 0.4474 | 0.0013 | **0** |  |
|  |  | Std | 0.0177 | 0.3503 | 0.2974 | 0.7589 | 0.3988 | 0.2728 | 0.2929 | 0.1884 | 0.0013 | **0** |  |
|  |  | P-value | 1.8214e-07 | 8.0065e-09 | 8.0065e-09 | 8.0065e-09 | 8.0065e-09 | 8.0065e-09 | 8.0065e-09 | 8.0065e-09 | 8.0065e-09 | **-** |  |
|  | Task2 | Mean | 69.5086 | 98.2676 | 104.0581 | 33.4881 | 116.9216 | 86.0457 | 93.4695 | 92.6134 | 43.7678 | **28.7488** |  |
|  |  | Std | 30.2752 | 42.1083 | 42.8453 | 24.4403 | 60.7051 | 39.8790 | 25.1838 | 30.4625 | 29.3195 | **3.4728** |  |
|  |  | P-value | 4.6958e-04 | 1.0646e-07 | 1.0646e-07 | 0.5792 | 1.8074e-05 | 1.8030e-06 | 1.2009e-06 | 0.1167 | 0.6554 | **-** |  |
|  | run time |  | 45.1073 | 23.9711 | 28.4164 | 41.3712 | 15.3974 | 16.1541 | 26.8961 | 16.3333 | 18.0274 | **12.0562** |  |
| 6 | Task1 | Mean | 0.0078 | 0.3541 | 0.3181 | 0.1078 | 0.3247 | 0.6721 | 0.3863 | 0.3238 | 0.0006 | **0** |  |
|  |  | Std | 0.0034 | 0.0840 | 0.0768 | 0.3140 | 0.0809 | 0.3398 | 0.1253 | 0.0999 | 0.0006 | **0** |  |
|  |  | P-value | 1.8214e-07 | 8.0065e-09 | 8.0065e-09 | 8.0065e-09 | 8.0065e-09 | 8.0065e-09 | 8.0065e-09 | 8.0065e-09 | 8.0065e-09 | **-** |  |
|  | Task2 | Mean | 0.1359 | 1.4264 | 1.3755 | 0.4663 | 1.3878 | 1.2296 | 1.4394 | 0.9007 | 0.1383 | **0** |  |
|  |  | Std | 0.0305 | 0.1844 | 0.2186 | 0.2159 | 0.1588 | 0.1791 | 0.2069 | 0.1496 | 0.0690 | **0** |  |
|  |  | P-value | 1.8214e-07 | 8.0065e-09 | 8.0065e-09 | 8.0065e-09 | 8.0065e-09 | 8.0065e-09 | 8.0065e-09 | 2.1518e-05 | 8.0065e-09 | **-** |  |
|  | run time |  | 51.4328 | 36.4051 | 44.0726 | 53.4250 | 29.0845 | 30.0345 | 38.1124 | 27.8354 | 28.9654 | **25.0328** |  |
| 7 | Task1 | Mean | 0.2599 | 15.8928 | 23.9193 | 33.8001 | 17.2246 | 24.2140 | 14.7936 | 15.9240 | 11.6804 | **0** |  |
|  |  | Std | 0.1448 | 3.1351 | 5.0561 | 11.1391 | 3.1973 | 5.8462 | 3.1364 | 4.1156 | 3.6569 | **0** |  |
|  |  | P-value | 1.8214e-07 | 8.0065e-09 | 8.0065e-09 | 8.0065e-09 | 8.0065e-09 | 8.0065e-09 | 8.0065e-09 | 8.0065e-09 | 8.0065e-09 | **-** |  |
|  | Task2 | Mean | 72.0445 | 97.4159 | 100.7158 | **29.8540** | 97.9908 | 97.3434 | 113.2386 | 97.8188 | 51.5326 | 32.1433 |  |
|  |  | Std | 35.4370 | 35.6230 | 28.5418 | **8.6396** | 43.5270 | 46.6113 | 57.4444 | 31.4901 | 35.3664 | 16.6833 |  |
|  |  | P-value | 0.0014 | 4.539e-07 | 4.539e-07 | 0.0639 | 4.539e-07 | 3.4156e-07 | 2.9598e-07 | 0.1604 | 0.7150 | - |  |
|  | run time |  | 45.0336 | 24.2641 | 29.4173 | 43.3907 | 16.1454 | 17.4576 | 26.7884 | 15.8866 | 16.9636 | **10.9071** |  |
| 8 | Task1 | Mean | 0.0165 | 0.7770 | 0.7439 | 0.1704 | 0.7694 | 0.9463 | 0.8054 | 0.8230 | 0.0040 | **0** |  |
|  |  | Std | 0.0157 | 0.1376 | 0.1344 | 0.1715 | 0.1453 | 0.1303 | 0.1200 | 0.0994 | 0.0057 | **0** |  |
|  |  | P-value | 1.8214e-07 | 8.0065e-09 | 8.0065e-09 | 8.0065e-09 | 8.0065e-09 | 8.0065e-09 | 8.0065e-09 | 8.0065e-09 | 8.0065e-09 | **-** |  |
|  | Task2 | Mean | 0.2681 | 1.5554 | 1.5301 | 0.7688 | 1.5719 | 1.2065 | 1.5856 | 0.8684 | 0.1271 | **0** |  |
|  |  | Std | 0.1142 | 0.1963 | 0.1816 | 0.4091 | 0.1867 | 0.1956 | 0.1693 | 0.1212 | 0.0454 | **0** |  |
|  |  | P-value | 1.8214e-07 | 8.0065e-09 | 8.0065e-09 | 8.0065e-09 | 8.0065e-09 | 8.0065e-09 | 8.0065e-09 | 2.1518e-05 | 8.0065e-09 | **-** |  |
|  | run time |  | 52.6870 | 35.6631 | 41.3456 | 52.3301 | 28.4588 | 30.6134 | 41.3324 | 28.6141 | 30.7128 | **26.4293** |  |
| 9 | Task1 | Mean | 0.2 | 16.5 | 23.3 | 97.7 | 15.3 | 26.2 | 16.8 | 19.9 | 25.5 | **0** |  |
|  |  | Std | 0.0901 | 3.1174 | 6.6179 | 20.4052 | 2.1104 | 7.1304 | 3.3623 | 5.0259 | 7.2174 | **0** |  |
|  |  | P-value | 1.8214e-07 | 8.0065e-09 | 8.0065e-09 | 8.0065e-09 | 8.0065e-09 | 8.0065e-09 | 8.0065e-09 | 8.0065e-09 | 8.0065e-09 | **-** |  |
|  | Task2 | Mean | 1.6982e+03 | 1.9133e+03 | 1.7811e+03 | 3.5338e+03 | 1.9642e+03 | 1.7361e+03 | **1.6711e+03** | 1.8715e+03 | 1.7356e+03 | 4.5326e+03 |  |
|  |  | Std | 279.4933 | **237.3464** | 290.2974 | 573.9668 | 285.3062 | 321.4278 | 318.1099 | 371.4803 | 386.4274 | 656.4530 |  |
|  |  | P-value | 1.2009e-05 | 6.7956e-08 | 6.7956e-08 | 5.2550e-05 | 6.7956e-08 | 6.7956e-08 | 6.7956e-08 | 0.1167 | 6.7956e-08 | - |  |
|  | run time |  | 39.9333 | 19.4119 | 23.7505 | 34.6596 | 13.0308 | 14.1564 | 21.6337 | 12.8438 | 13.6655 | **9.0428** |  |
