# Supplementary material for: Multi-task snake optimization algorithm for global optimization and planar kinematic arm control problem
Source: PeerJ Comput Sci. 2025 Feb 11;11:e2688. doi: 10.7717/peerj-cs.2688 (PMC11888922; doi:10.7717/peerj-cs.2688)
Supplement: Supplemental Information 19 [file peerj-cs-11-2688-s019.docx]

| Test Combinations | |  | SAMTO | MFEA | MFEARR | LDAMFEA | MFEALBS | EBSGA | GMFEA | EMTEA | MTEA | MTSO |  |
| --- | --- | --- | --- | --- | --- | --- | --- | --- | --- | --- | --- | --- | --- |
| 1 | Task1 | Mean | 10.0007 | 11.0522 | 11.0208 | 10.8458 | 11.0492 | 11.0108 | 11.0222 | 10.9140 | 10.3307 | **10** |  |
|  |  | Std | 0.0004 | 0.0339 | 0.0121 | 0.2468 | 0.0211 | 0.0175 | 0.0389 | 0.0958 | 0.0815 | **0** |  |
|  |  | p-value | 6.3864e-05 | 6.3864e-05 | 6.3864e-05 | 6.3864e-05 | 6.3864e-05 | 6.3864e-05 | 6.3864e-05 | 6.3864e-05 | 6.3864e-05 | **-** |  |
|  | Task2 | Mean | 10.0003 | 22.1661 | 33.1851 | 12.3083 | 21.7948 | 31.4878 | 12.0631 | 22.9343 | 23.6540 | **10** |  |
|  |  | Std | 0.0002 | 8.0770 | 3.7587 | 3.3575 | 6.8213 | 9.7010 | 0.6762 | 4.8967 | 3.7461 | **0** |  |
|  |  | p-value | 6.3864e-05 | 6.3864e-05 | 6.3864e-05 | 6.3864e-05 | 6.3864e-05 | 6.3864e-05 | 6.3864e-05 | 0.1818 | 6.3864e-05 | **-** |  |
|  | run time |  | 48.475 | 23.4591 | 28.7640 | 42.5013 | 15.7559 | 17.1184 | 26.1091 | 15.4868 | 16.5772 | **9.833** |  |
| 2 | Task1 | Mean | 10.0105 | 11.1892 | 10.6775 | 12.4526 | 11.2583 | 10.6488 | 11.0016 | 10.4852 | 10.1982 | **10** |  |
|  |  | Std | 0.0042 | 0.3905 | 0.2930 | 0.5516 | 0.3409 | 0.2983 | 0.3444 | 0.2933 | 0.0672 | **0** |  |
|  |  | p-value | 6.3864e-05 | 6.3864e-05 | 6.3864e-05 | 6.3864e-05 | 6.3864e-05 | 6.3864e-05 | 6.3864e-05 | 6.3864e-05 | 6.3864e-05 | **-** |  |
|  | Task2 | Mean | 10.0011 | 22.9403 | 39.0216 | 22.6929 | 23.8359 | 30.3381 | 13.1910 | 24.6026 | 22.3765 | **10** |  |
|  |  | Std | 0.0009 | 6.4943 | 8.1703 | 4.6181 | 5.3002 | 8.2905 | 3.2688 | 2.8066 | 4.0868 | **0** |  |
|  |  | p-value | 6.3864e-05 | 6.3864e-05 | 6.3864e-05 | 6.3864e-05 | 6.3864e-05 | 6.3864e-05 | 6.3864e-05 | 0.1818 | 6.3864e-05 | **-** |  |
|  | run time |  | 41.286 | 21.6697 | 27.0815 | 38.2364 | 15.0453 | 16.8033 | 25.8573 | 14.7800 | 15.4621 | **9.737** |  |
| 3 | Task1 | Mean | **10** | 11.3 | 10.6 | 14.8 | 11.2 | 10.7 | 11.0 | 10.5 | **10** | **10** |  |
|  |  | Std | 0.0145 | 0.3606 | 0.1995 | 1.2186 | 0.4533 | 0.4650 | 0.2180 | 0.1080 | 0.0302 | **0** |  |
|  |  | p-value | 6.3864e-05 | 6.3864e-05 | 6.3864e-05 | 6.3864e-05 | 6.3864e-05 | 0.1818 | 6.3864e-05 | 6.3864e-05 | 6.3864e-05 | **-** |  |
|  | Task2 | Mean | **1.5368e+03** | 1.9820e+03 | 1.6631e+03 | 3.4482e+03 | 2.0063e+03 | 1.6933e+03 | 1.8636e+03 | 1.6527e+03 | 1.8315e+03 | 3.9555e+03 |  |
|  |  | Std | **250.2424** | 477.9580 | 411.1307 | 543.3261 | 367.2894 | 283.8110 | 283.0637 | 348.3263 | 356.5711 | 919.8908 |  |
|  |  | p-value | 1.8267e-04 | 1.8267e-04 | 1.8267e-04 | 0.4274 | 1.8267e-04 | 1.8267e-04 | 1.8267e-04 | 0.1818 | 1.8267e-04 | - |  |
|  | run time |  | 44.325 | 22.6617 | 27.9563 | 41.7159 | 15.4353 | 16.5753 | 25.2840 | 15.0218 | 16.1239 | **10.047** |  |
| 4 | Task1 | Mean | 10.0003 | 18.4632 | 37.0405 | 10.0302 | 23.3919 | 30.1704 | 12.4131 | 22.2392 | 21.0861 | **10** |  |
|  |  | Std | 0.0002 | 7.2876 | 3.0776 | 0.0290 | 5.8891 | 6.8777 | 0.4754 | 3.3141 | 4.7030 | **0** |  |
|  |  | p-value | 6.3864e-05 | 6.3864e-05 | 6.3864e-05 | 6.3864e-05 | 6.3864e-05 | 6.3864e-05 | 6.3864e-05 | 6.3864e-05 | 6.3864e-05 | **-** |  |
|  | Task2 | Mean | **10** | 10.0160 | 10.0088 | 10.0002 | 10.0163 | 10.0083 | 10.0122 | 10.0042 | 10.0007 | **10** |  |
|  |  | Std | **0** | 0.0042 | 0.0028 | 0.0002 | 0.0047 | 0.0021 | 0.0024 | 0.0021 | 0.0004 | **0** |  |
|  |  | p-value | 6.3864e-05 | 6.3864e-05 | 6.3864e-05 | 6.3864e-05 | 6.3864e-05 | 6.3864e-05 | 6.3864e-05 | 0.1818 | 6.3864e-05 | **-** |  |
|  | run time |  | 42.089 | 23.3309 | 29.5363 | 40.8091 | 15.5381 | 16.9854 | 26.0091 | 15.2488 | 13.7762 | **7.874** |  |
| 5 | Task1 | Mean | 10.0417 | 11.1647 | 10.5103 | 13.1881 | 11.0718 | 10.6097 | 11.1838 | 10.3701 | 10.0037 | **10** |  |
|  |  | Std | 0.0114 | 0.4749 | 0.1204 | 0.8932 | 0.2239 | 0.2014 | 0.3759 | 0.1100 | 0.0045 | **0** |  |
|  |  | p-value | 6.3864e-05 | 6.3864e-05 | 6.3864e-05 | 6.3864e-05 | 6.3864e-05 | 6.3864e-05 | 6.3864e-05 | 6.3864e-05 | 6.3864e-05 | **-** |  |
|  | Task2 | Mean | 83.7798 | 95.8520 | 86.9020 | 43.3304 | 115.1157 | 132.2997 | 139.1000 | 99.8994 | 79.4019 | **38.2876** |  |
|  |  | Std | 21.8488 | 45.6634 | 35.2674 | 17.2054 | 35.1739 | 72.8975 | 50.5977 | 34.7151 | 31.9576 | **0.8658** |  |
|  |  | p-value | 0.0028 | 0.0211 | 0.0173 | 0.9097 | 1.8267e-04 | 1.8267e-04 | 1.8267e-04 | 0.1818 | 0.1041 | **-** |  |
|  | run time |  | 43.221 | 22.7892 | 27.5848 | 38.7720 | 15.1517 | 16.4239 | 25.1570 | 14.6352 | 15.7326 | **9.771** |  |
| 6 | Task1 | Mean | 10.0074 | 10.3819 | 10.3520 | 10.1715 | 10.3807 | 10.7010 | 10.4145 | 10.3045 | 10.0004 | **10** |  |
|  |  | Std | 0.0024 | 0.0783 | 0.0753 | 0.4254 | 0.1143 | 0.4480 | 0.0931 | 0.0622 | 0.0004 | **0** |  |
|  |  | p-value | 6.3864e-05 | 6.3864e-05 | 6.3864e-05 | 6.3864e-05 | 6.3864e-05 | 6.3864e-05 | 6.3864e-05 | 6.3864e-05 | 6.3864e-05 | **-** |  |
|  | Task2 | Mean | 10.1419 | 11.4734 | 11.4259 | 10.6034 | 11.4504 | 11.2005 | 11.5628 | 11.9000 | 10.1514 | **10** |  |
|  |  | Std | 0.0289 | 0.1589 | 0.1251 | 0.5724 | 0.2401 | 0.1657 | 0.1935 | 0.1366 | 0.0630 | **0** |  |
|  |  | p-value | 6.3864e-05 | 6.3864e-05 | 6.3864e-05 | 6.3864e-05 | 6.3864e-05 | 6.3864e-05 | 6.3864e-05 | 0.1818 | 6.3864e-05 | **-** |  |
|  | run time |  | 52.837 | 35.9140 | 42.8797 | 51.6958 | 27.8508 | 28.8004 | 37.7383 | 27.4866 | 28.8540 | **24.051** |  |
| 7 | Task1 | Mean | 10.2587 | 25.6990 | 35.4189 | 40.1412 | 26.7818 | 32.7076 | 28.3435 | 27.8744 | 23.4709 | **10** |  |
|  |  | Std | 0.1060 | 2.3959 | 7.8990 | 7.3986 | 3.4329 | 8.8554 | 3.6908 | 2.8039 | 2.8258 | **0** |  |
|  |  | p-value | 6.3864e-05 | 6.3864e-05 | 6.3864e-05 | 6.3864e-05 | 6.3864e-05 | 6.3864e-05 | 6.3864e-05 | 6.3864e-05 | 6.3864e-05 | **-** |  |
|  | Task2 | Mean | 94.6885 | 104.5505 | 108.8248 | **37.9496** | 113.0444 | 88.6591 | 111.7771 | 84.4852 | 75.2232 | 38.2599 |  |
|  |  | Std | 35.8293 | 29.1413 | 40.6339 | 1.0084 | 38.0231 | 30.1330 | 38.7332 | 34.3627 | 37.0461 | **0.6885** |  |
|  |  | p-value | 0.0028 | 1.8267e-04 | 1.8267e-04 | 0.1859 | 1.8267e-04 | 1.8267e-04 | 1.8267e-04 | 0.1818 | 0.2413 | **-** |  |
|  | run time |  | 46.398 | 22.2308 | 27.3875 | 40.1320 | 15.7565 | 16.5845 | 25.0912 | 14.9420 | 15.7830 | **9.759** |  |
| 8 | Task1 | Mean | 10.0225 | 10.7407 | 10.7442 | 10.1375 | 10.7258 | 10.9946 | 10.8173 | 10.7922 | 10.0092 | **10** |  |
|  |  | Std | 0.0178 | 0.1917 | 0.1061 | 0.0973 | 0.1596 | 0.0457 | 0.1368 | 0.1509 | 0.0132 | **0** |  |
|  |  | p-value | 6.3864e-05 | 6.3864e-05 | 6.3864e-05 | 6.3864e-05 | 6.3864e-05 | 6.3864e-05 | 6.3864e-05 | 6.3864e-05 | 6.3864e-05 | **-** |  |
|  | Task2 | Mean | 10.2910 | 11.5105 | 11.4987 | 10.6690 | 11.4624 | 11.1831 | 11.5750 | 10.8799 | 10.1102 | **10** |  |
|  |  | Std | 0.1073 | 0.2175 | 0.1142 | 0.2401 | 0.1721 | 0.1770 | 0.1614 | 0.0991 | 0.0925 | **0** |  |
|  |  | p-value | 6.3864e-05 | 6.3864e-05 | 6.3864e-05 | 6.3864e-05 | 6.3864e-05 | 6.3864e-05 | 6.3864e-05 | 0.1818 | 6.3864e-05 | **-** |  |
|  | run time |  | 50.113 | 34.4686 | 39.5348 | 49.8755 | 27.3690 | 28.7603 | 39.2262 | 27.2688 | 28.0101 | **23.382** |  |
| 9 | Task1 | Mean | 10.1 | 25.9 | 31.5 | 99.6 | 25.1 | 34.6 | 27.2 | 31.2 | 34.1 | **10** |  |
|  |  | Std | 0.0568 | 3.3173 | 4.0681 | 23.1852 | 3.0272 | 5.5083 | 3.0711 | 2.0975 | 6.4926 | **0** |  |
|  |  | p-value | 6.3864e-05 | 6.3864e-05 | 6.3864e-05 | 6.3864e-05 | 6.3864e-05 | 6.3864e-05 | 6.3864e-05 | 6.3864e-05 | 6.3864e-05 | **-** |  |
|  | Task2 | Mean | **1.6767e+03** | 2.0877e+03 | 2.0293e+03 | 3.3335e+03 | 1.8214e+03 | 1.7965e+03 | 1.8089e+03 | 1.8708e+03 | 1.9523e+03 | 4.4536e+03 |  |
|  |  | Std | **201.3052** | 295.7972 | 491.0466 | 623.4152 | 392.8547 | 280.1191 | 271.9370 | 279.0046 | 316.8564 | 607.6476 |  |
|  |  | p-value | 1.8267e-04 | 1.8267e-04 | 1.8267e-04 | 0.0036 | 1.8267e-04 | 1.8267e-04 | 1.8267e-04 | 0.1818 | 1.8267e-04 | - |  |
|  | run time |  | 39.668 | 18.9909 | 23.0260 | 33.5925 | 12.6675 | 13.6826 | 20.8503 | 12.3916 | 13.1330 | **7.978** |  |
