# Supplementary material for: Multi-task snake optimization algorithm for global optimization and planar kinematic arm control problem
Source: PeerJ Comput Sci. 2025 Feb 11;11:e2688. doi: 10.7717/peerj-cs.2688 (PMC11888922; doi:10.7717/peerj-cs.2688)
Supplement: Supplemental Information 20 [file peerj-cs-11-2688-s020.doc]

|  | **Population size** | **30** | **50** | **100** |
| --- | --- | --- | --- | --- |
| 1 | Task1 average rank | 2.30 | 1.95 | 1.75 |
|  | Task2 average rank | 2 | 2 | 2 |
| 2 | Task1 average rank | 2 | 2 | 2 |
|  | Task2 average rank | 2.45 | 1.80 | 1.75 |
| 3 | Task1 average rank | 2 | 2 | 2 |
|  | Task2 average rank | 2.45 | 1.85 | 1.70 |
| 4 | Task1 average rank | 2 | 2 | 2 |
|  | Task2 average rank | 2 | 2 | 2 |
| 5 | Task1 average rank | 2 | 2 | 2 |
|  | Task2 average rank | 2.30 | 1.90 | 1.80 |
| 6 | Task1 average rank | 2.15 | 2 | 1.85 |
|  | Task2 average rank | 2 | 2 | 2 |
| 7 | Task1 average rank | 2 | 2 | 2 |
|  | Task2 average rank | 2.15 | 2 | 1.85 |
| 8 | Task1 average rank | 2 | 2 | 2 |
|  | Task2 average rank | 2 | 2 | 2 |
| 9 | Task1 average rank | 2 | 2 | 2 |
|  | Task2 average rank | 2.20 | 1.95 | 1.85 |
|  | Total ranking | 38 | 35.45 | 34.55 |
|  | Rank | 3 | 2 | 1 |
