# Supplementary material for: Multi-task snake optimization algorithm for global optimization and planar kinematic arm control problem
Source: PeerJ Comput Sci. 2025 Feb 11;11:e2688. doi: 10.7717/peerj-cs.2688 (PMC11888922; doi:10.7717/peerj-cs.2688)
Supplement: Supplemental Information 21 [file peerj-cs-11-2688-s021.doc]

|  | parameter | RMP = 0.9,   R1 = 0.95 | RMP = 0.9,   R1 = 0.85 | RMP = 0.9,  R1 = 0.75 | RMP = 0.7,  R1 = 0.95 | RMP = 0.7,  R1 = 0.85 | RMP = 0.7,   R1 = 0.75 | RMP = 0.5,  R1 = 0.95 | RMP = 0.5,  R1 = 0.85 | RMP = 0.5,  R1 = 0.75 | RMP = 0.3,  R1 = 0.95 | RMP = 0.3,   R1 = 0.85 | RMP = 0.3,   R1 = 0.75 |
| --- | --- | --- | --- | --- | --- | --- | --- | --- | --- | --- | --- | --- | --- |
| 1 | Task1 | 6.5 | 6.5 | 6.5 | 6.5 | 6.5 | 6.5 | 6.5 | 6.5 | 6.5 | 6.5 | 6.5 | 6.5 |
|  | Task2 | 6.5 | 6.5 | 6.5 | 6.5 | 6.5 | 6.5 | 6.5 | 6.5 | 6.5 | 6.5 | 6.5 | 6.5 |
| 2 | Task1 | 6.5 | 6.5 | 6.5 | 6.5 | 6.5 | 6.5 | 6.5 | 6.5 | 6.5 | 6.5 | 6.5 | 6.5 |
|  | Task2 | 6.5 | 6.5 | 6.5 | 6.5 | 6.5 | 6.5 | 6.5 | 6.5 | 6.5 | 6.5 | 6.5 | 6.5 |
| 3 | Task1 | 6.5 | 6.5 | 6.5 | 6.5 | 6.5 | 6.5 | 6.5 | 6.5 | 6.5 | 6.5 | 6.5 | 6.5 |
|  | Task2 | 7.8 | 6.4 | 5.3 | 4.9 | 5.3 | 6.1 | 5.0 | 6.1 | 7.2 | 9.4 | 6.5 | 8.0 |
| 4 | Task1 | 6.5 | 6.5 | 6.5 | 6.5 | 6.5 | 6.5 | 6.5 | 6.5 | 6.5 | 6.5 | 6.5 | 6.5 |
|  | Task2 | 6.5 | 6.5 | 6.5 | 6.5 | 6.5 | 6.5 | 6.5 | 6.5 | 6.5 | 6.5 | 6.5 | 6.5 |
| 5 | Task1 | 6.5 | 6.5 | 6.5 | 6.5 | 6.5 | 6.5 | 6.5 | 6.5 | 6.5 | 6.5 | 6.5 | 6.5 |
|  | Task2 | 9.0 | 6.7 | 6.2 | 6.9 | 6.1 | 7.0 | 5.1 | 7.6 | 5.8 | 6.0 | 4.7 | 6.9 |
| 6 | Task1 | 6.5 | 6.5 | 6.5 | 6.5 | 6.5 | 6.5 | 6.5 | 6.5 | 6.5 | 6.5 | 6.5 | 6.5 |
|  | Task2 | 6.5 | 6.5 | 6.5 | 6.5 | 6.5 | 6.5 | 6.5 | 6.5 | 6.5 | 6.5 | 6.5 | 6.5 |
| 7 | Task1 | 6.5 | 6.5 | 6.5 | 6.5 | 6.5 | 6.5 | 6.5 | 6.5 | 6.5 | 6.5 | 6.5 | 6.5 |
|  | Task2 | 7.1 | 7.2 | 7.8 | 7.4 | 5.8 | 7.4 | 6.2 | 7.4 | 5.5 | 5.2 | 4.8 | 6.2 |
| 8 | Task1 | 6.5 | 6.5 | 6.5 | 6.5 | 6.5 | 6.5 | 6.5 | 6.5 | 6.5 | 6.5 | 6.5 | 6.5 |
|  | Task2 | 6.5 | 6.5 | 6.5 | 6.5 | 6.5 | 6.5 | 6.5 | 6.5 | 6.5 | 6.5 | 6.5 | 6.5 |
| 9 | Task1 | 6.5 | 6.5 | 6.5 | 6.5 | 6.5 | 6.5 | 6.5 | 6.5 | 6.5 | 6.5 | 6.5 | 6.5 |
|  | Task2 | 5.4 | 5.5 | 6.2 | 6.7 | 5.5 | 8.4 | 5.6 | 7.1 | 6.9 | 7.1 | 6.3 | 7.3 |
|  | Total ranking | 120.3 | 116.8 | 116.5 | 116.9 | 113.7 | 119.9 | 112.9 | 119.2 | 116.4 | 118.5 | 113.1 | 119.2 |
|  | Rank | 11 | 6 | 5 | 7 | 3 | 10 | 1 | 9 | 4 | 8 | 2 | 9 |
