# Supplementary material for: Multi-task snake optimization algorithm for global optimization and planar kinematic arm control problem
Source: PeerJ Comput Sci. 2025 Feb 11;11:e2688. doi: 10.7717/peerj-cs.2688 (PMC11888922; doi:10.7717/peerj-cs.2688)
Supplement: Supplemental Information 22 [file peerj-cs-11-2688-s022.doc]

| **Num** | **Population size** | **1/10*N** | **1/5*N** | **1/2*N** |
| --- | --- | --- | --- | --- |
| 1 | Task1 average rank | 2 | 2 | 2 |
|  | Task2 average rank | 2 | 2 | 2 |
| 2 | Task1 average rank | 2 | 2 | 2 |
|  | Task2 average rank | 2 | 2 | 2 |
| 3 | Task1 average rank | 2 | 2 | 2 |
|  | Task2 average rank | 2 | 1.4 | 2.6 |
| 4 | Task1 average rank | 2 | 2 | 2 |
|  | Task2 average rank | 2 | 2 | 2 |
| 5 | Task1 average rank | 2 | 2 | 2 |
|  | Task2 average rank | 1.6 | 2 | 2.4 |
| 6 | Task1 average rank | 2 | 2 | 2 |
|  | Task2 average rank | 2 | 2 | 2 |
| 7 | Task1 average rank | 2 | 2 | 2 |
|  | Task2 average rank | 1.7 | 2.1 | 2.2 |
| 8 | Task1 average rank | 2 | 2 | 2 |
|  | Task2 average rank | 2 | 2 | 2 |
| 9 | Task1 average rank | 2 | 2 | 2 |
|  | Task2 average rank | 2.5 | 2 | 1.5 |
|  | Total ranking | 35.8 | 35.5 | 36.7 |
|  | Rank | 2 | 1 | 3 |
