# Supplementary material for: Multi-task snake optimization algorithm for global optimization and planar kinematic arm control problem
Source: PeerJ Comput Sci. 2025 Feb 11;11:e2688. doi: 10.7717/peerj-cs.2688 (PMC11888922; doi:10.7717/peerj-cs.2688)
Supplement: Supplemental Information 23 [file peerj-cs-11-2688-s023.doc]

| **Number** | **Task** | **Knowledge utilization rate** | **Task** | **Knowledge utilization rate** |
| --- | --- | --- | --- | --- |
| 1 | Task1 | 0.3214 | Task2 | 0.2731 |
| 2 | Task1 | 0.3506 | Task2 | 0.2866 |
| 3 | Task1 | 0.3029 | Task2 | 0.5500 |
| 4 | Task1 | 0.2462 | Task2 | 0.4296 |
| 5 | Task1 | 0.3350 | Task2 | 0.6253 |
| 6 | Task1 | 0.3951 | Task2 | 0.2001 |
| 7 | Task1 | 0.1324 | Task2 | 0.6745 |
| 8 | Task1 | 0.3098 | Task2 | 0.1982 |
| 9 | Task1 | 0.1473 | Task2 | 0.4723 |
