# Supplementary material for: Multi-task snake optimization algorithm for global optimization and planar kinematic arm control problem
Source: PeerJ Comput Sci. 2025 Feb 11;11:e2688. doi: 10.7717/peerj-cs.2688 (PMC11888922; doi:10.7717/peerj-cs.2688)
Supplement: Supplemental Information 24 [file peerj-cs-11-2688-s024.doc]

|  | **Algorithm Name** | **Mean** | **Std** | **Run time** |
| --- | --- | --- | --- | --- |
| 5 tasks in 5 dimensions | MTSO | **0.3076** | 1.7022e-05 | **8.8439** |
|  | MFEA | 0.3211 | 2.0709e-05 | 17.6964 |
|  | MFEARR | 0.3210 | 7.3031e-09 | 22.4309 |
|  | EBSGA | 0.3230 | 0.0045 | 34.2621 |
|  | GMFEA | 0.3210 | 1.3364e-06 | 19.0942 |
|  | EMTEA | 0.3211 | **2.6471e-10** | 31.4896 |
|  | MTEA | 0.3210 | 7.2178e-09 | 35.7847 |
| 5 tasks in 10 dimensions | MTSO | **0.3158** | 4.9406e-05 | **11.7468** |
|  | MFEA | 0.3284 | 1.9342e-06 | 18.6173 |
|  | MFEARR | 0.3283 | 1.0711e-07 | 23.7195 |
|  | EBSGA | 0.3288 | 0.0012 | 36.6925 |
|  | GMFEA | 0.3283 | 1.4112e-06 | 20.1048 |
|  | EMTEA | 0.3283 | **4.9627e-08** | 34.0945 |
|  | MTEA | 0.3283 | 6.8272e-08 | 37.6432 |
| 5 tasks in 20 dimensions | MTSO | **0.3201** | 9.4061e-05 | **17.5135** |
|  | MFEA | 0.3322 | 4.2842e-06 | 19.3467 |
|  | MFEARR | 0.3321 | 1.7749e-06 | 24.3141 |
|  | EBSGA | 0.3326 | 0.0012 | 38.5810 |
|  | GMFEA | 0.3322 | 8.0129e-07 | 20.7831 |
|  | EMTEA | 0.3321 | **1.4033e-07** | 35.9231 |
|  | MTEA | 0.3321 | 2.8983e-07 | 39.5354 |
| 10 tasks in 5 dimensions | MTSO | **0.2926** | 7.9567e-05 | 30.7927 |
|  | MFEA | 0.3228 | 6.6682e-06 | **28.5592** |
|  | MFEARR | 0.3228 | 1.2774e-05 | 34.7434 |
|  | EBSGA | 0.3232 | 6.5676e-04 | 82.7234 |
|  | GMFEA | 0.3228 | 5.2002e-06 | 30.8124 |
|  | EMTEA | 0.3228 | 5.2904e-09 | 75.6532 |
|  | MTEA | 0.3228 | **3.0057e-09** | 75.5142 |
| 10 tasks in 10 dimensions | MTSO | **0.2976** | 2.6395e-05 | 49.6888 |
|  | MFEA | 0.3281 | 1.5316e-06 | **30.9493** |
|  | MFEARR | 0.3281 | 4.8060e-07 | 37.2470 |
|  | EBSGA | 0.3319 | 0.0049 | 92.7837 |
|  | GMFEA | 0.3281 | 1.1479e-07 | 33.6735 |
|  | EMTEA | 0.3281 | **2.7757e-08** | 98.2108 |
|  | MTEA | 0.3281 | 3.6312e-08 | 100.3812 |
| 10 tasks in 20 dimensions | MTSO | **0.3026** | 4.4554e-04 | 66.9099 |
|  | MFEA | 0.3315 | 0.0039 | **31.1880** |
|  | MFEARR | 0.3313 | 0.0018 | 36.7254 |
|  | EBSGA | 0.3327 | 0.0029 | 100.485 |
|  | GMFEA | 0.3314 | 6.5085e-04 | 33.5761 |
|  | EMTEA | 0.3313 | **2.0622e-06** | 94.9603 |
|  | MTEA | 0.3313 | 2.0644e-06 | 98.5762 |
