# Supplementary material for: Multi-task snake optimization algorithm for global optimization and planar kinematic arm control problem
Source: PeerJ Comput Sci. 2025 Feb 11;11:e2688. doi: 10.7717/peerj-cs.2688 (PMC11888922; doi:10.7717/peerj-cs.2688)
Supplement: Supplemental Information 25 [file peerj-cs-11-2688-s025.doc]

| **Algorithm Name** | **Mean** | **Std** | **Run time(s)** |
| --- | --- | --- | --- |
| MTSO | **1.5689** | 0.9686 | **86.9822** |
| MFEA | 3.2169 | 0.3019 | 127.2030 |
| MFEARR | 3.5121 | 0.6358 | 103.0602 |
| EBSGA | 3.7202 | **0.2078** | 90.6607 |
| GMFEA | 3.3260 | 0.3632 | 120.1103 |
| EMTEA | 3.6792 | 0.3385 | 128.9005 |
| MTEA | 3.1415 | 0.2240 | 122.9216 |
