# Supplementary material for: Multi-task snake optimization algorithm for global optimization and planar kinematic arm control problem
Source: PeerJ Comput Sci. 2025 Feb 11;11:e2688. doi: 10.7717/peerj-cs.2688 (PMC11888922; doi:10.7717/peerj-cs.2688)
Supplement: Supplemental Information 26 [file peerj-cs-11-2688-s026.doc]

| **Algorithm Name** | **Mean** | **Std** | **Run time(s)** |
| --- | --- | --- | --- |
| MTSO | **17.9085** | 0.1558 | **4.3714** |
| MFEA | 23.1184 | 0.0988 | 9.9254 |
| MFEARR | 23.0441 | 0.0398 | 12.3935 |
| EBSGA | 23.0849 | 0.0826 | 6.8581 |
| GMFEA | 23.0744 | 0.1276 | 11.1446 |
| EMTEA | 23.0296 | **0.0138** | 6.1104 |
| MTEA | 23.0364 | 0.0145 | 6.5282 |
