# Supplementary material for: Multi-task snake optimization algorithm for global optimization and planar kinematic arm control problem
Source: PeerJ Comput Sci. 2025 Feb 11;11:e2688. doi: 10.7717/peerj-cs.2688 (PMC11888922; doi:10.7717/peerj-cs.2688)
Supplement: Supplemental Information 27 [file peerj-cs-11-2688-s027.pdf]

Supplemental Figure S1 Convergence curve plots for 9 sets of multitask benchmark test functions

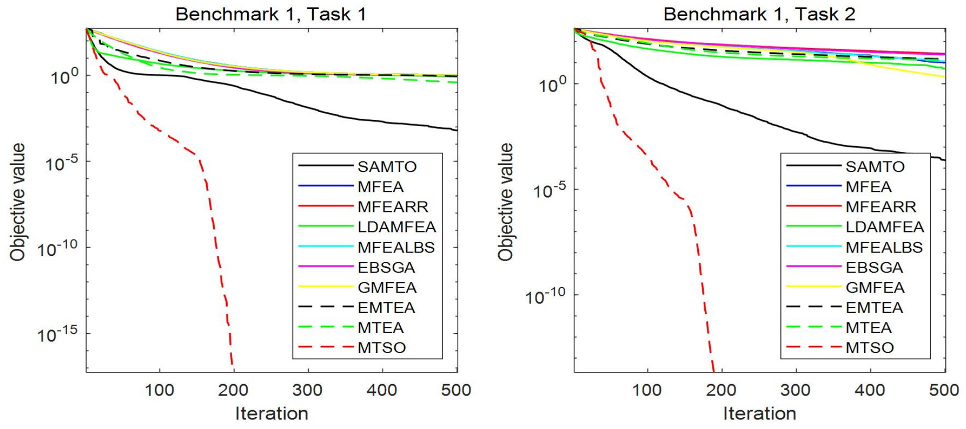

(a)

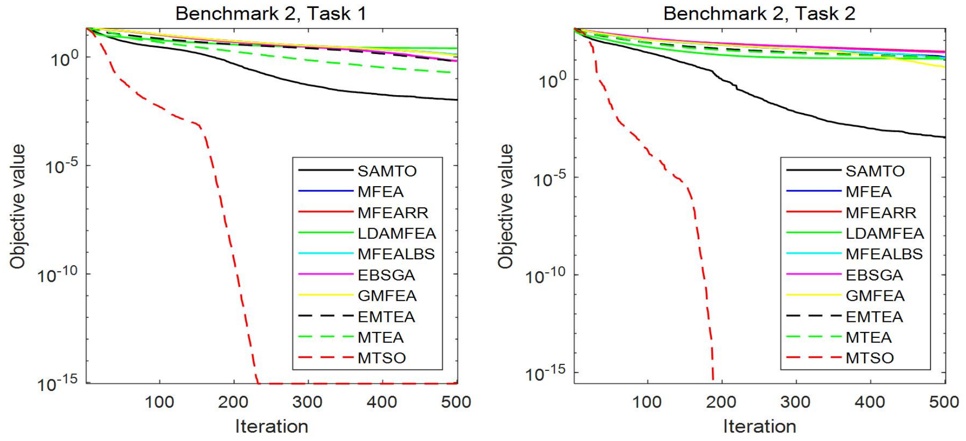

(b)

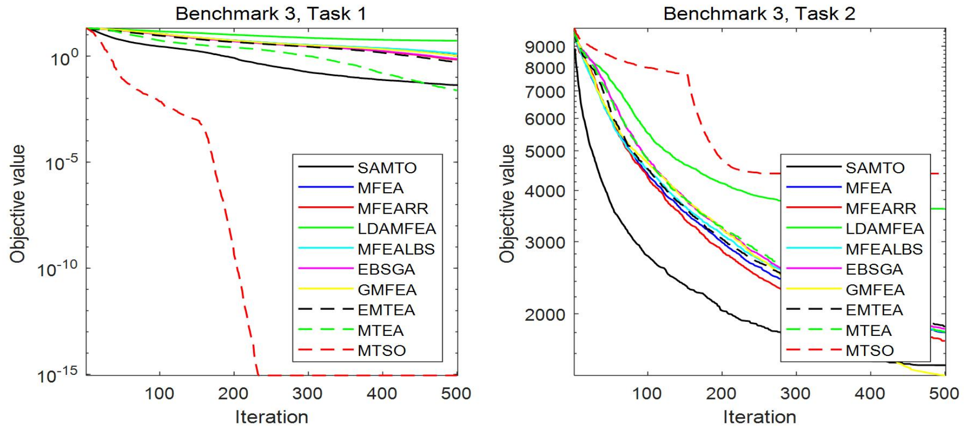

(c)

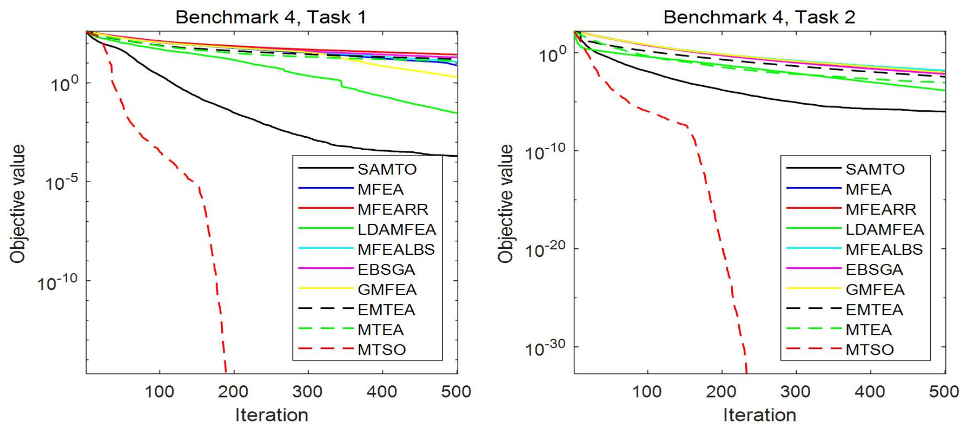
Benchmark 4, Task 1
Benchmark 4, Task 2
Objective value
Iteration
SAMTO
MFEA
MFEARR
LDAMFEA
MFEALBS
EBSGA
GMFEA
EMTEA
MTEA
MTSO

(d)

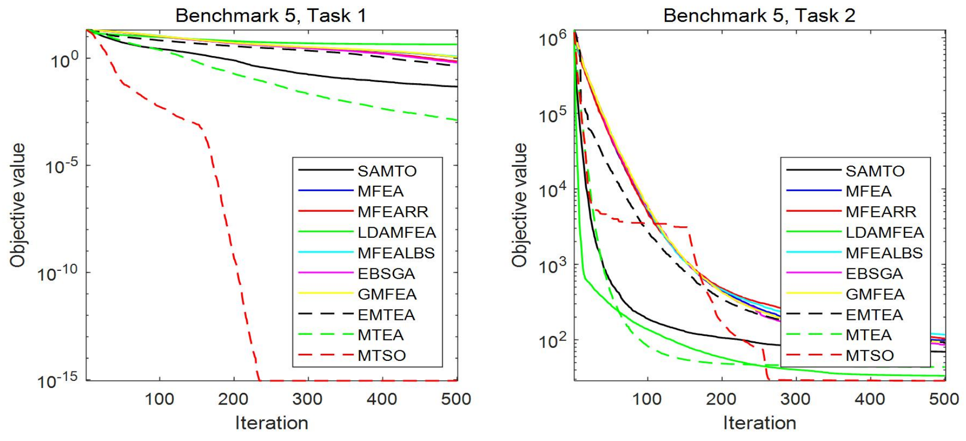
Benchmark 5, Task 1
Benchmark 5, Task 2
Objective value
Iteration
SAMTO
MFEA
MFEARR
LDAMFEA
MFEALBS
EBSGA
GMFEA
EMTEA
MTEA
MTSO

(e)

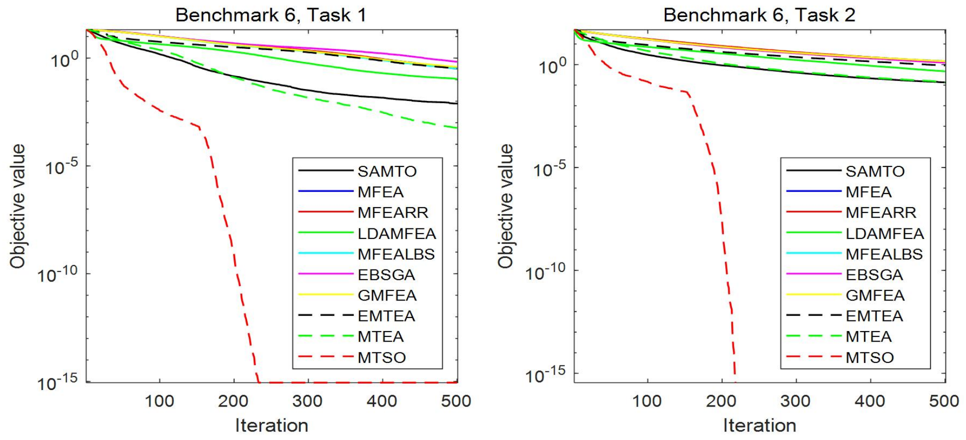
Benchmark 6, Task 1
Benchmark 6, Task 2
Objective value
Iteration
SAMTO
MFEA
MFEARR
LDAMFEA
MFEALBS
EBSGA
GMFEA
EMTEA
MTEA
MTSO

(f)

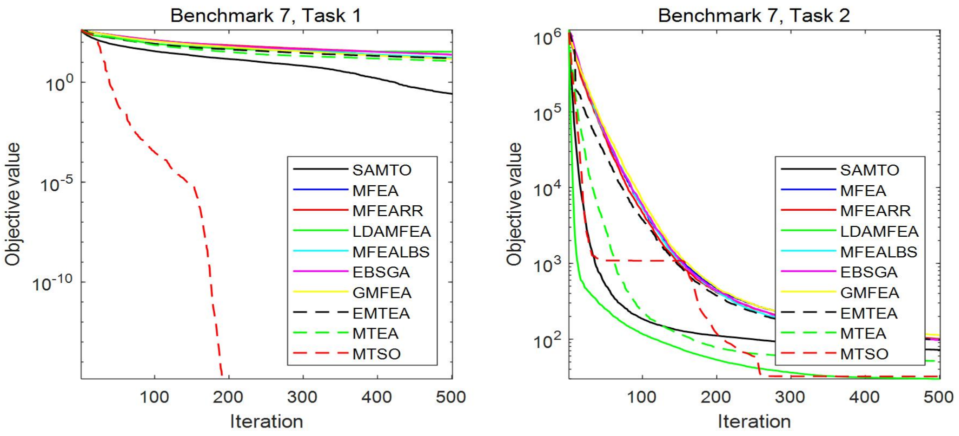
Benchmark 7, Task 1
Benchmark 7, Task 2
Objective value
Iteration
SAMTO
MFEA
MFEARR
LDAMFEA
MFEALBS
EBSGA
GMFEA
EMTEA
MTEA
MTSO

(g)

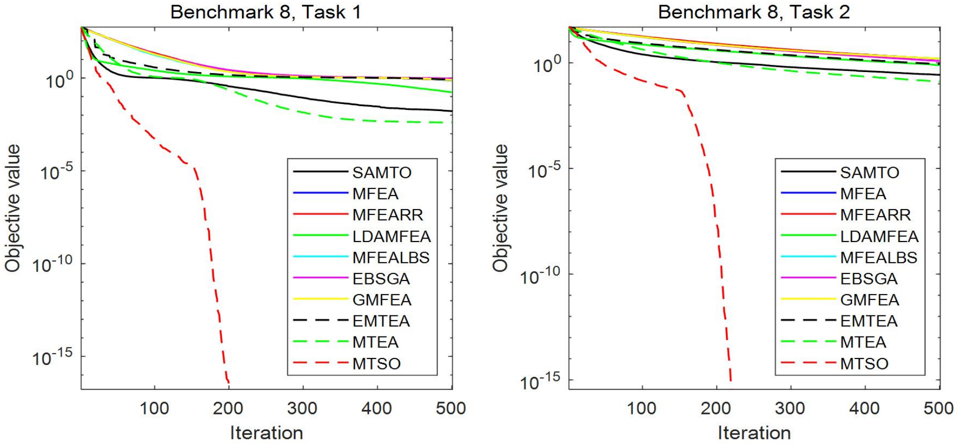
Benchmark 8, Task 1
Benchmark 8, Task 2
Objective value
Iteration
SAMTO
MFEA
MFEARR
LDAMFEA
MFEALBS
EBSGA
GMFEA
EMTEA
MTEA
MTSO

(h)

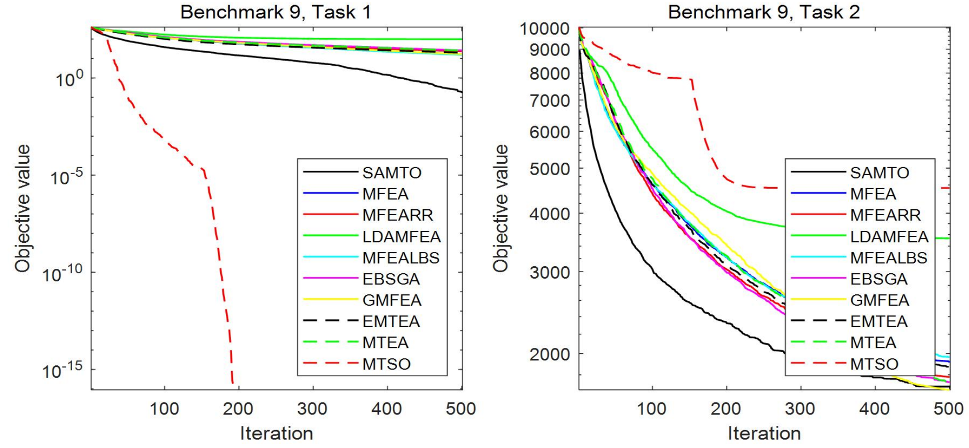
Benchmark 9, Task 1
Benchmark 9, Task 2
Objective value
Iteration
SAMTO
MFEA
MFEARR
LDAMFEA
MFEALBS
EBSGA
GMFEA
EMTEA
MTEA
MTSO

(i)

Supplemental Figure S2 Error bars for 9 sets of multitask benchmark test functions

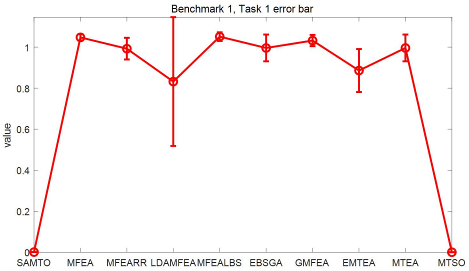

(1a)

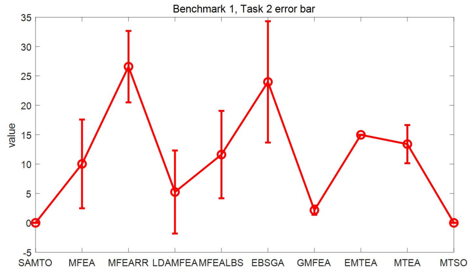

(1b)

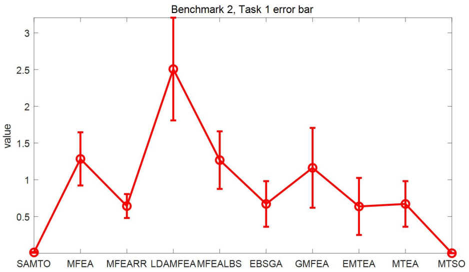

(2a)

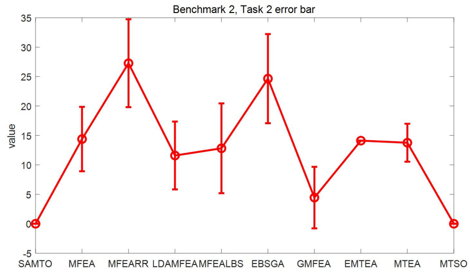

(2b)

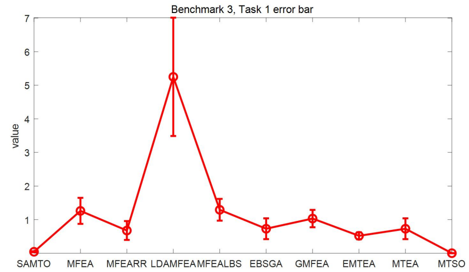

(3a)

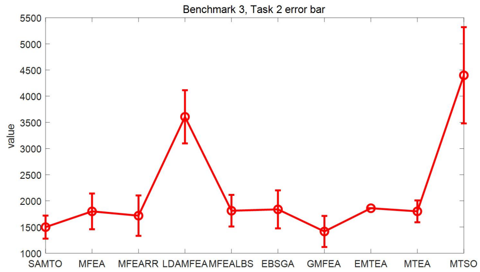

(3b)

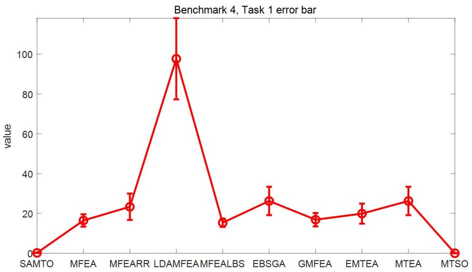

(4a)

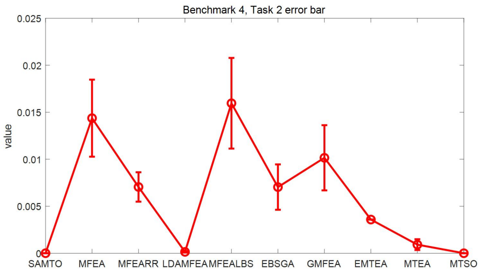

(4b)

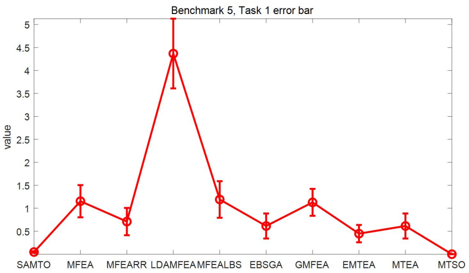

(5a)

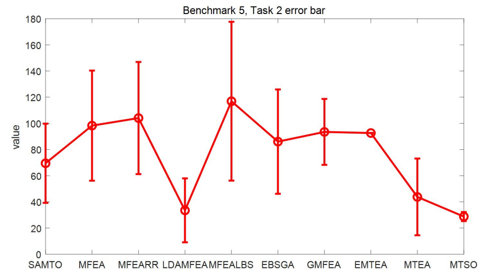

(5b)

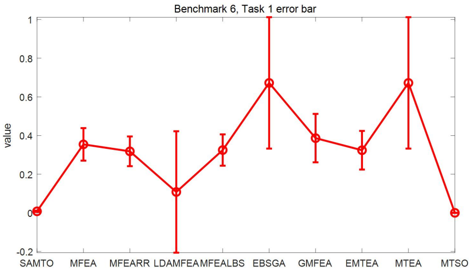

(6a)

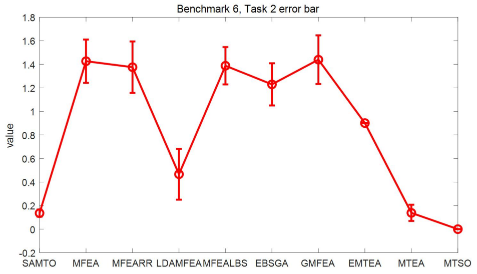

(6b)

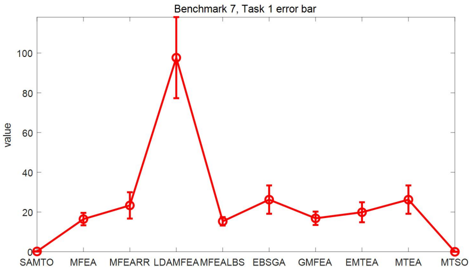

(7a)

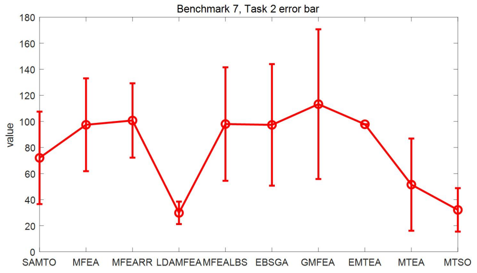

(7b)

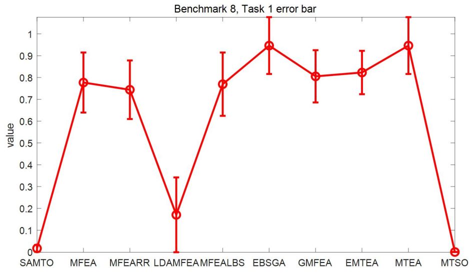

(8a)

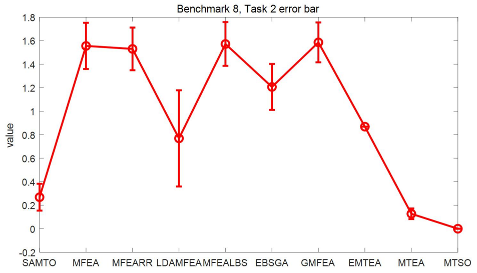

(8b)

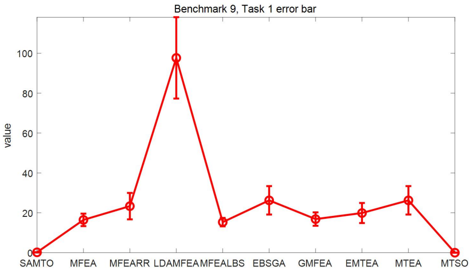

(9a)

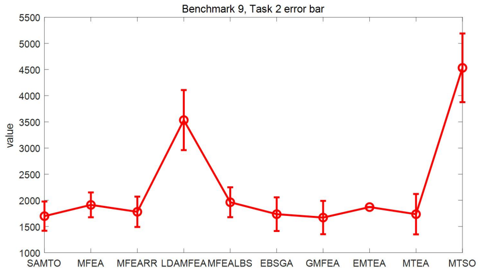

(9b)

Supplemental Figure S3 Noise Test Function Convergence curve plots for 9 sets of multitask benchmark test functions

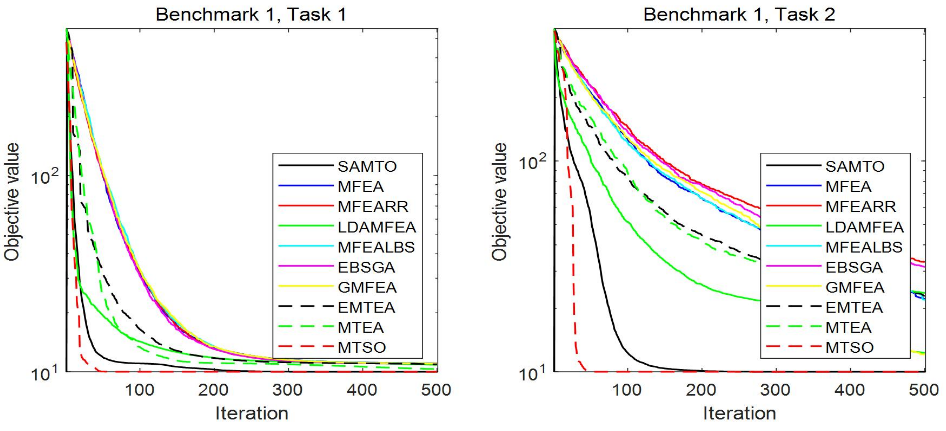

(a)

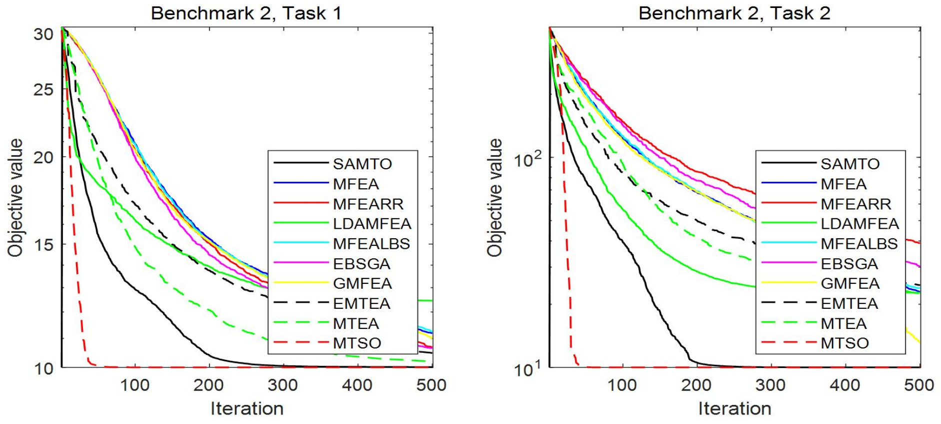

(b)

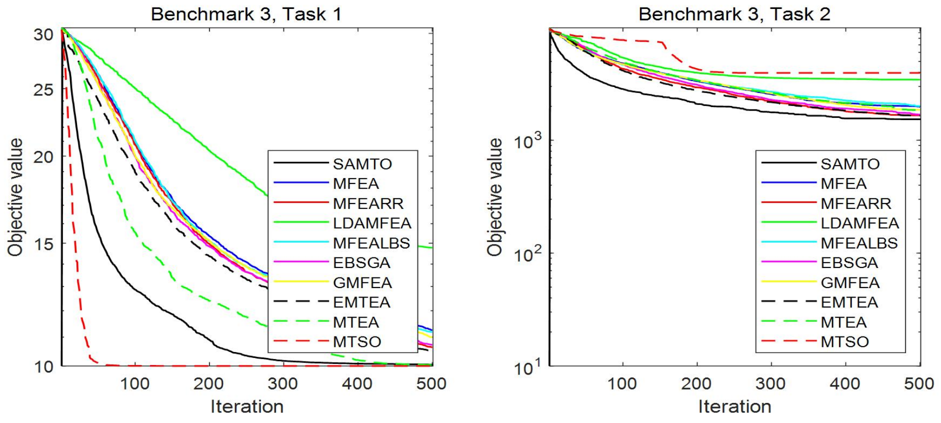

(c)

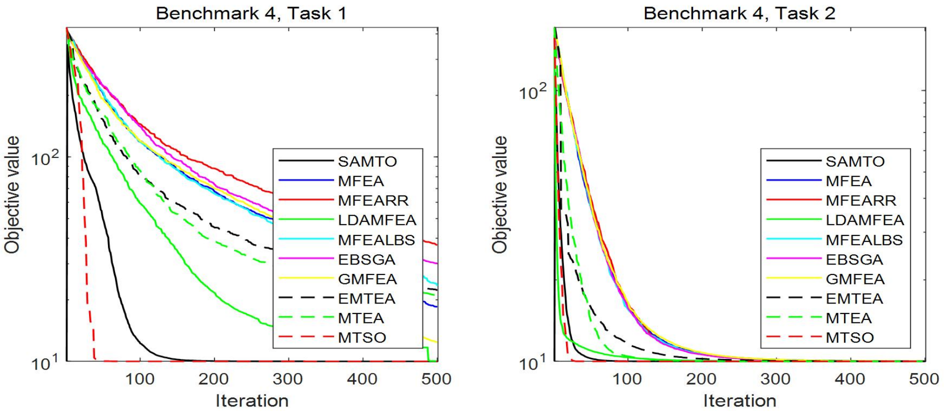
Benchmark 4, Task 1
Benchmark 4, Task 2
Objective value
Iteration
SAMTO
MFEA
MFEARR
LDAMFEA
MFEALBS
EBSGA
GMFEA
EMTEA
MTEA
MTSO

(d)

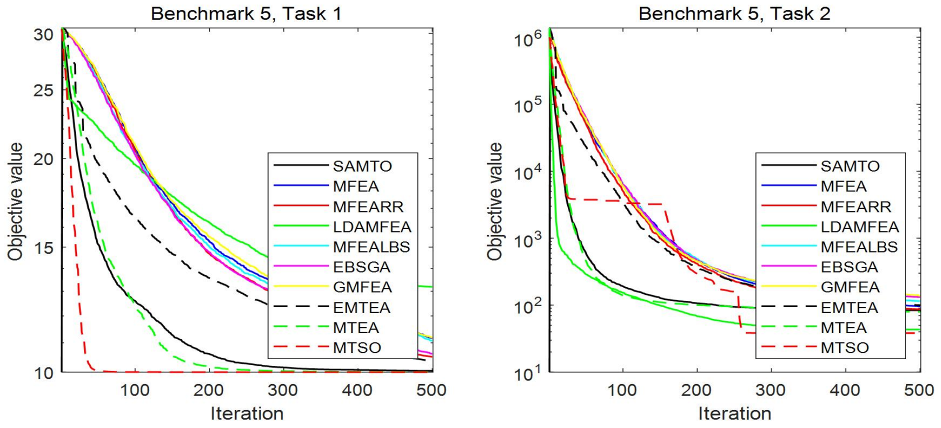
Benchmark 5, Task 1
Benchmark 5, Task 2
Objective value
Iteration
SAMTO
MFEA
MFEARR
LDAMFEA
MFEALBS
EBSGA
GMFEA
EMTEA
MTEA
MTSO

(e)

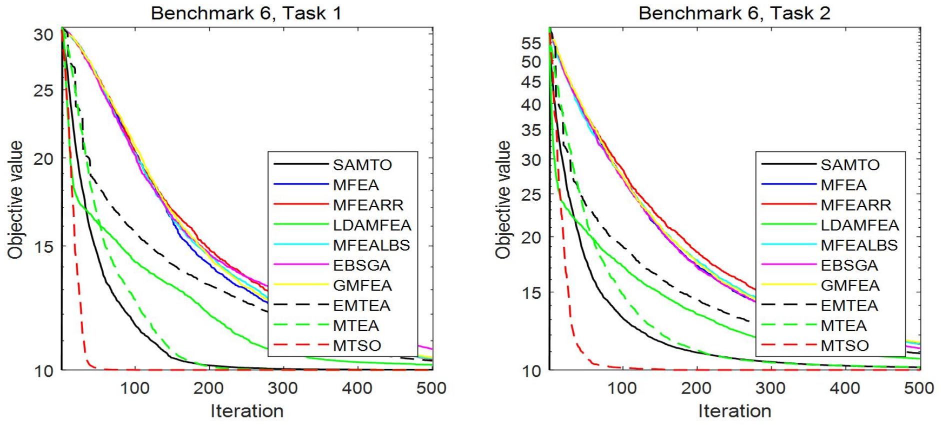
Benchmark 6, Task 1
Benchmark 6, Task 2
Objective value
Iteration
SAMTO
MFEA
MFEARR
LDAMFEA
MFEALBS
EBSGA
GMFEA
EMTEA
MTEA
MTSO

(f)

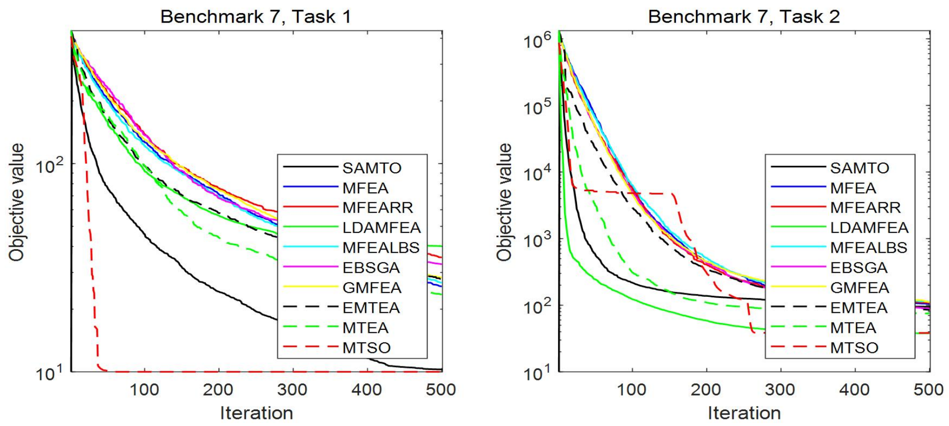
Benchmark 7, Task 1
Objective value
Iteration
SAMTO
MFEA
MFEARR
LDAMFEA
MFEALBS
EBSGA
GMFEA
EMTEA
MTEA
MTSO
Benchmark 7, Task 2
Objective value
Iteration
SAMTO
MFEA
MFEARR
LDAMFEA
MFEALBS
EBSGA
GMFEA
EMTEA
MTEA
MTSO

(g)

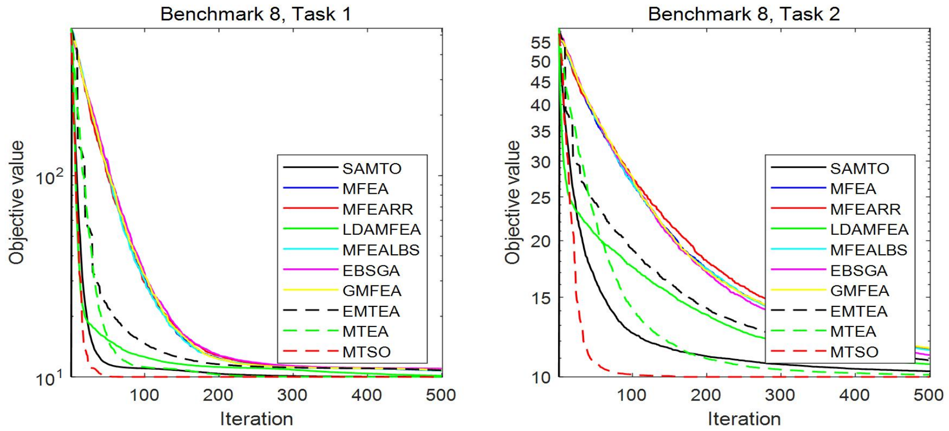
Benchmark 8, Task 1
Objective value
Iteration
SAMTO
MFEA
MFEARR
LDAMFEA
MFEALBS
EBSGA
GMFEA
EMTEA
MTEA
MTSO
Benchmark 8, Task 2
Objective value
Iteration
SAMTO
MFEA
MFEARR
LDAMFEA
MFEALBS
EBSGA
GMFEA
EMTEA
MTEA
MTSO

(h)

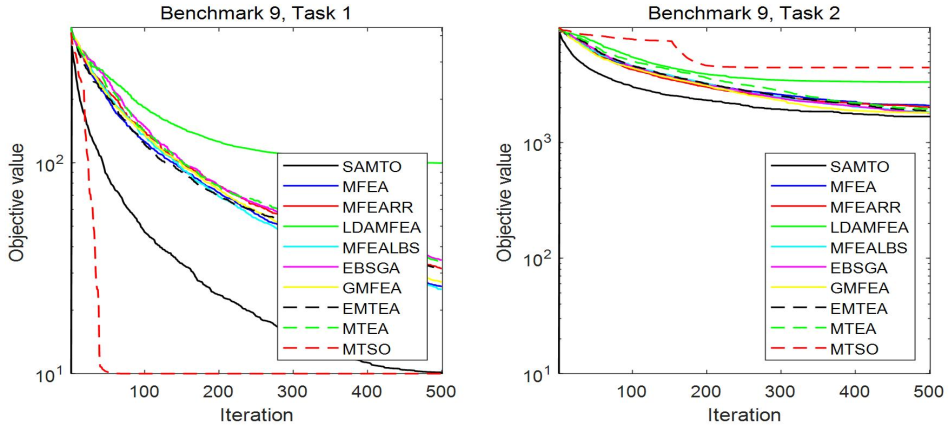
Benchmark 9, Task 1
Objective value
Iteration
SAMTO
MFEA
MFEARR
LDAMFEA
MFEALBS
EBSGA
GMFEA
EMTEA
MTEA
MTSO
Benchmark 9, Task 2
Objective value
Iteration
SAMTO
MFEA
MFEARR
LDAMFEA
MFEALBS
EBSGA
GMFEA
EMTEA
MTEA
MTSO

(i)

Supplemental Figure S4 The error bar chart for the 9 sets of noisy multitask benchmark test functions

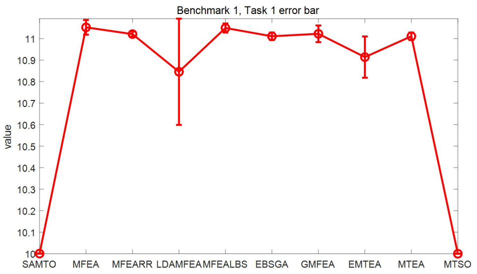

(1a)

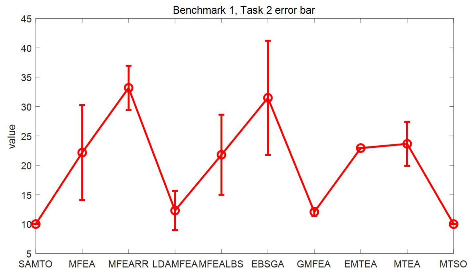

(1b)

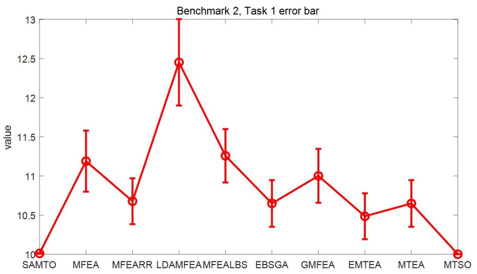

(2a)

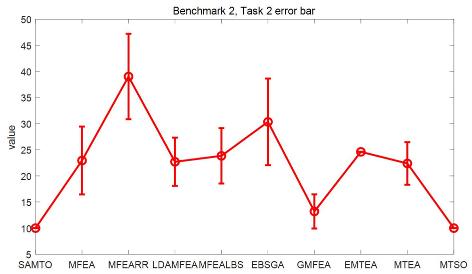

(2b)

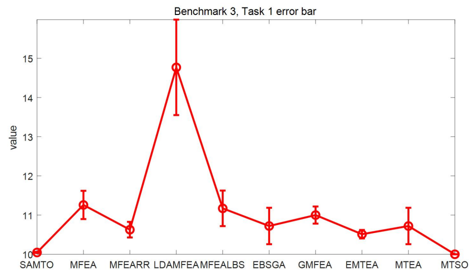

(3a)

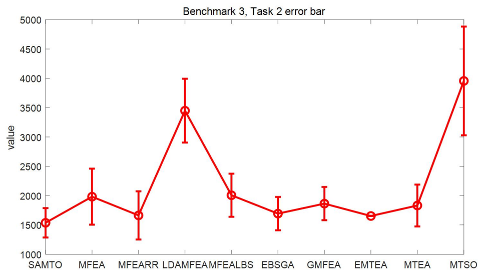

(3b)

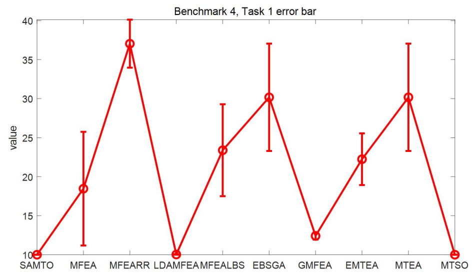

(4a)

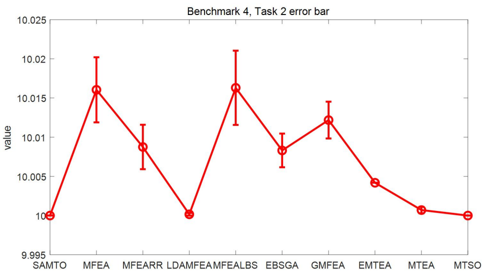

(4b)

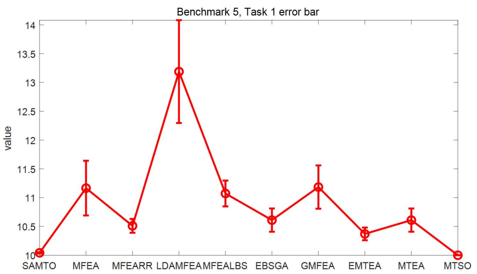

(5a)

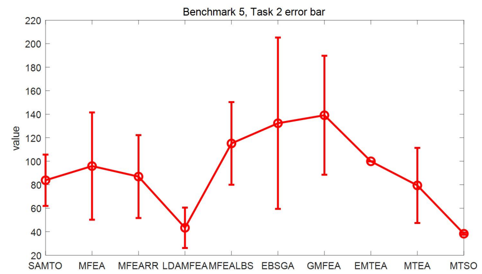

(5b)

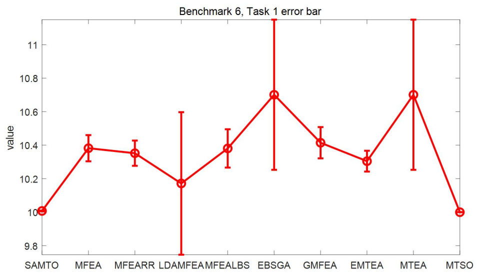

(6a)

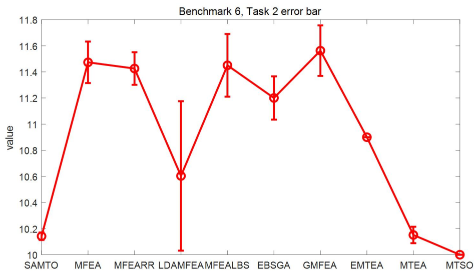

(6b)

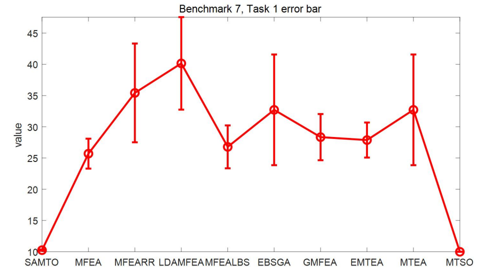

(7a)

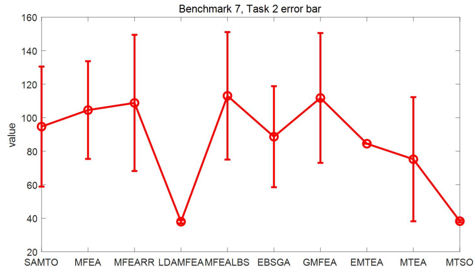

(7b)

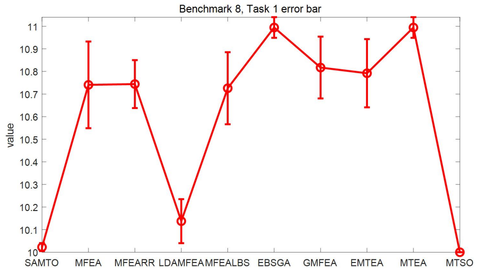

(8a)

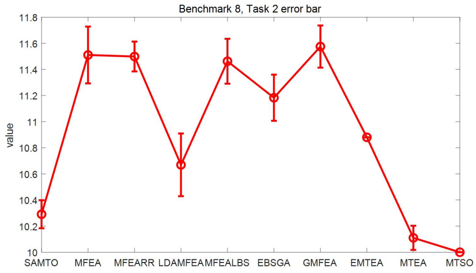

(8b)

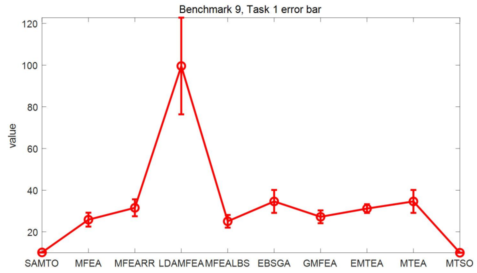

(9a)

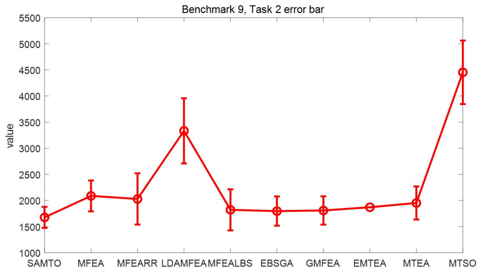

(9b)

Supplemental Figure S5 Knowledge Transfer Status During the Iterative Process

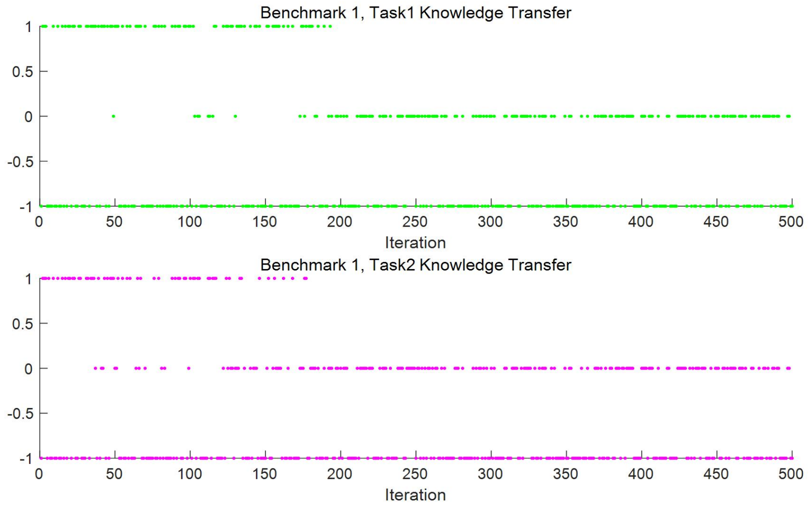

(1)

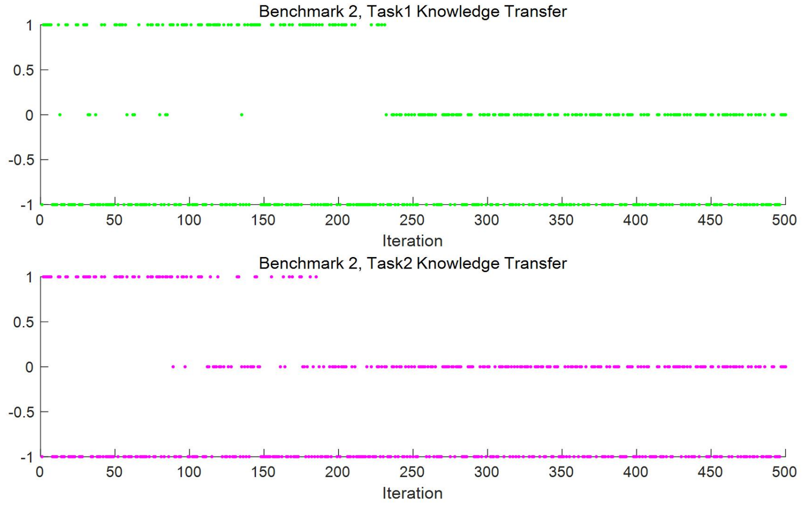

(2)

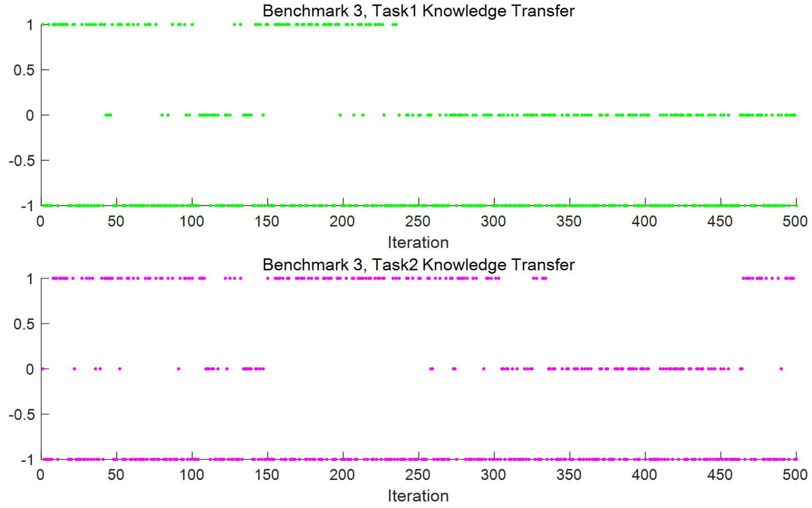

(3)

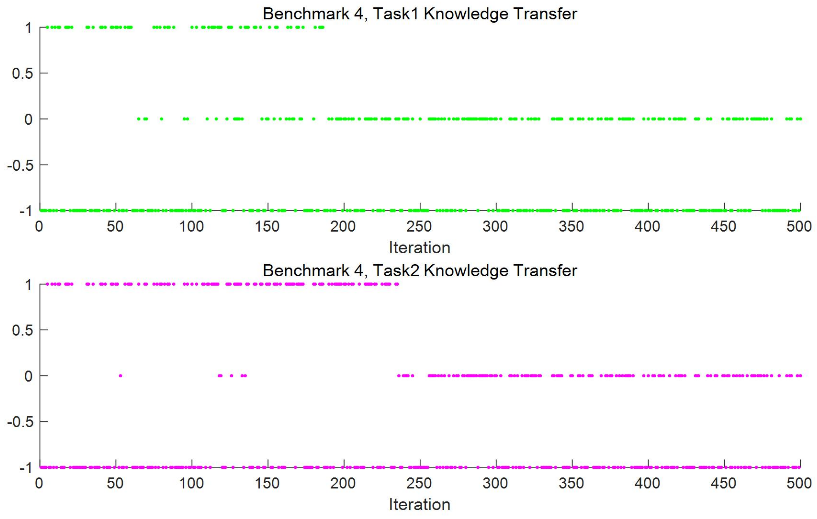
Benchmark 4, Task1 Knowledge Transfer
Benchmark 4, Task2 Knowledge Transfer
1
0.5
0
-0.5
-1
0
50
100
150
200
250
300
350
400
450
500
Iteration

(4)

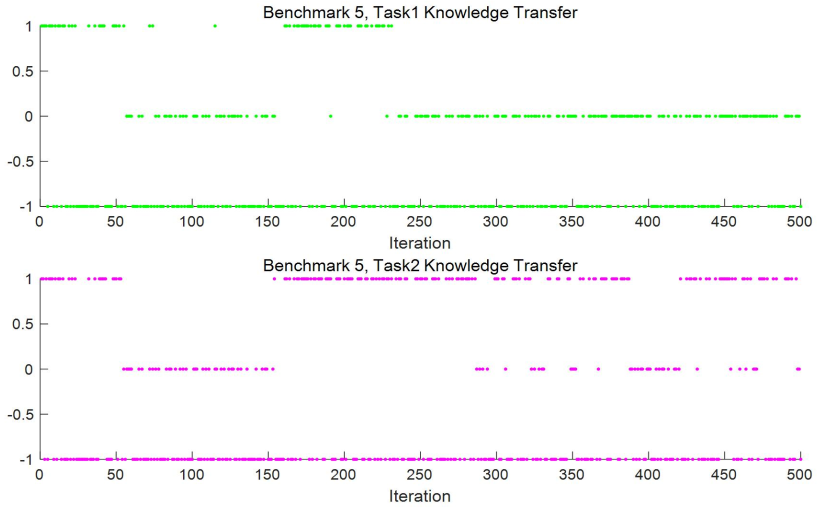
Benchmark 5, Task1 Knowledge Transfer
Benchmark 5, Task2 Knowledge Transfer
1
0.5
0
-0.5
-1
0
50
100
150
200
250
300
350
400
450
500
Iteration

(5)

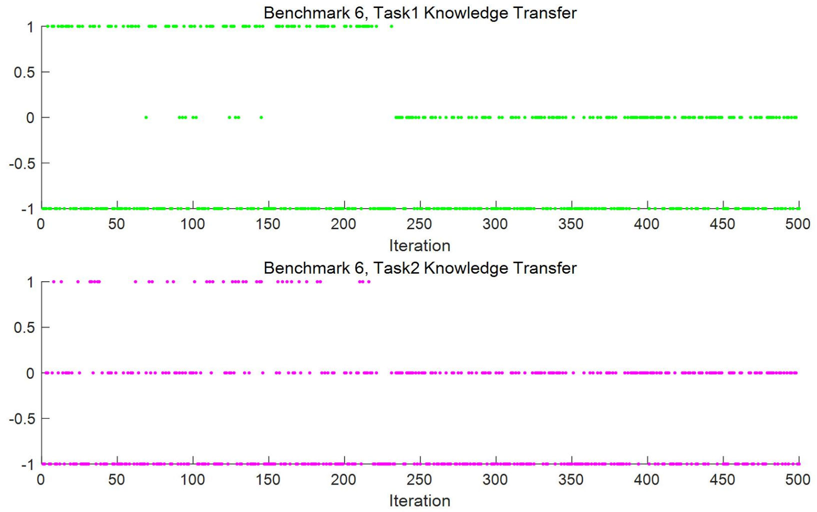
Benchmark 6, Task1 Knowledge Transfer
Benchmark 6, Task2 Knowledge Transfer
1
0.5
0
-0.5
-1
0
50
100
150
200
250
300
350
400
450
500
Iteration

(6)

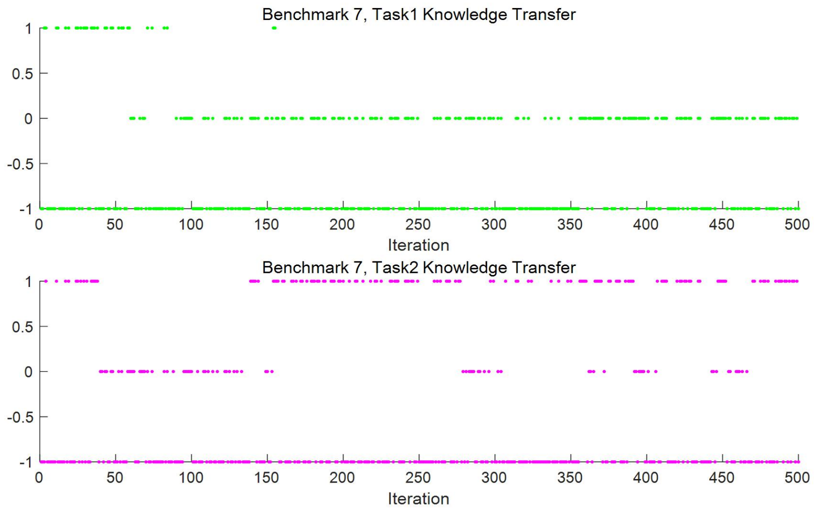
Benchmark 7, Task1 Knowledge Transfer
Iteration
Benchmark 7, Task2 Knowledge Transfer
Iteration

(7)

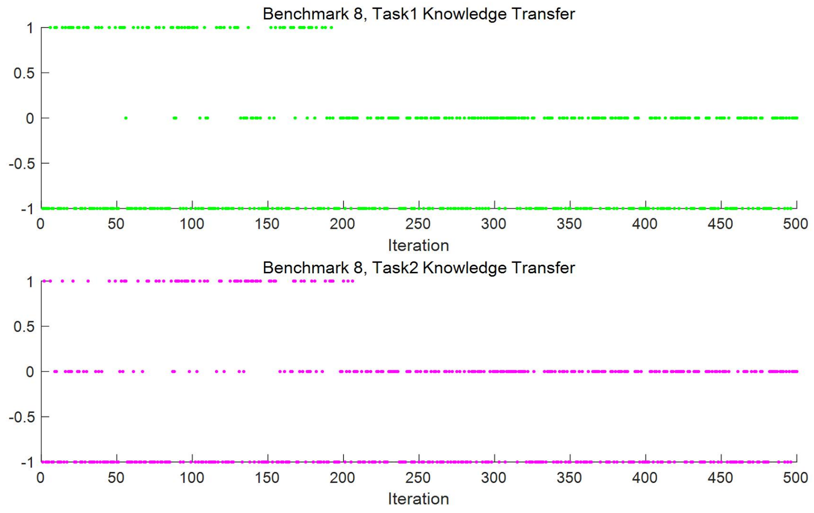
Benchmark 8, Task1 Knowledge Transfer
Iteration
Benchmark 8, Task2 Knowledge Transfer
Iteration

(8)

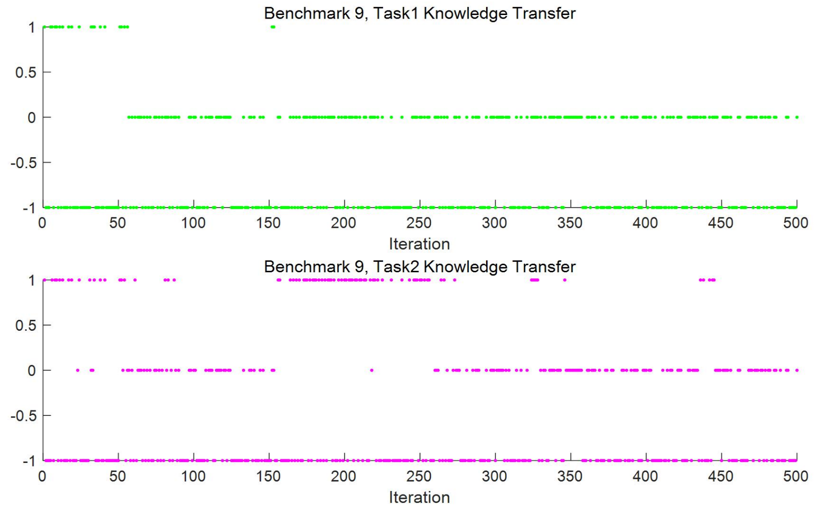
Benchmark 9, Task1 Knowledge Transfer
Iteration
Benchmark 9, Task2 Knowledge Transfer
Iteration

(9)

Supplemental Figure S6 Schematic Diagram of the PKACP for Two Tasks. Task 1 (a), Task 2 (b)

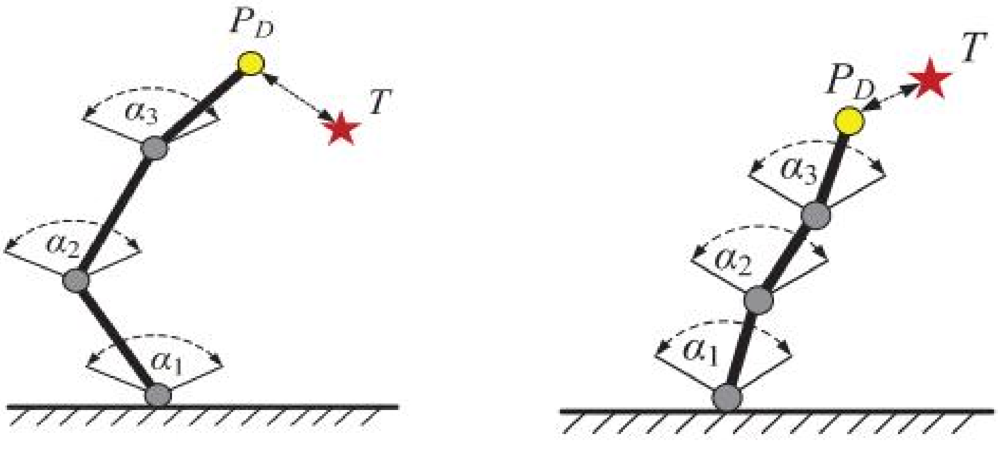

(a) (b)

Supplemental Figure S7 Average convergence curve (a) and error bar (b) for the PKACP with 5 tasks in 5 dimensions

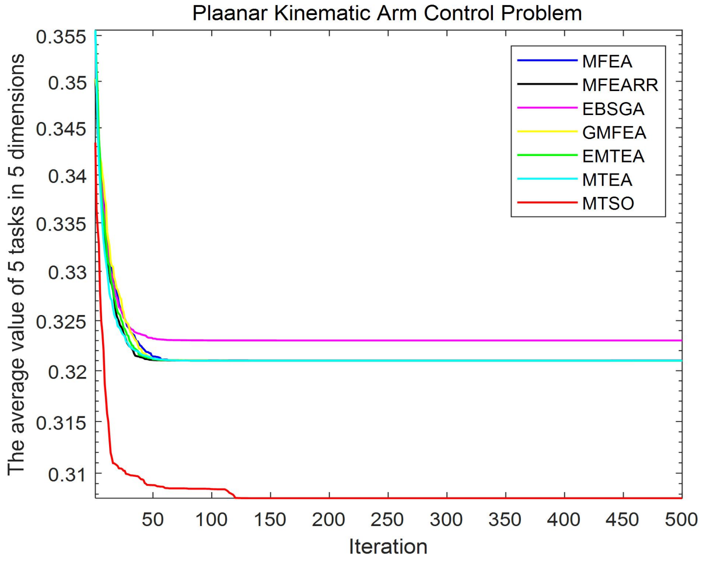

(a)

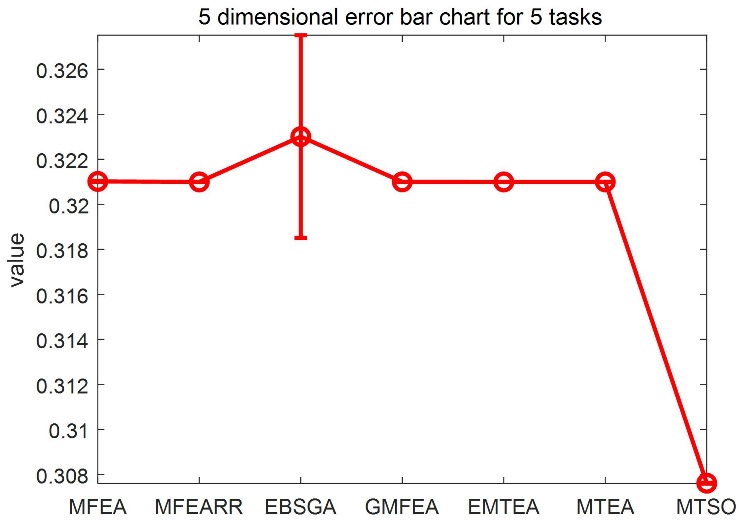

(b)

Supplemental Figure S8 Average convergence curve(a) and error bar (b) for the PKACP with 5 tasks in 10 dimensions

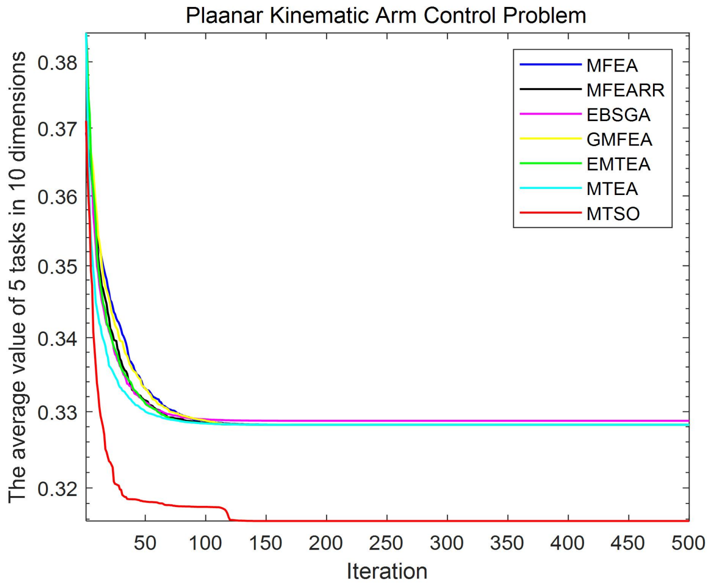

(a)

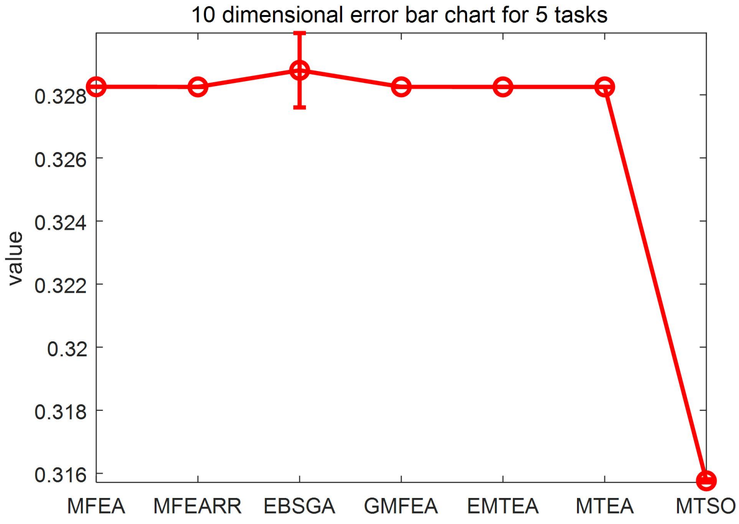

(b)

Supplemental Figure S9 Average convergence curve (a) and error bar (b) for the PKACP with 5 tasks in 20 dimensions

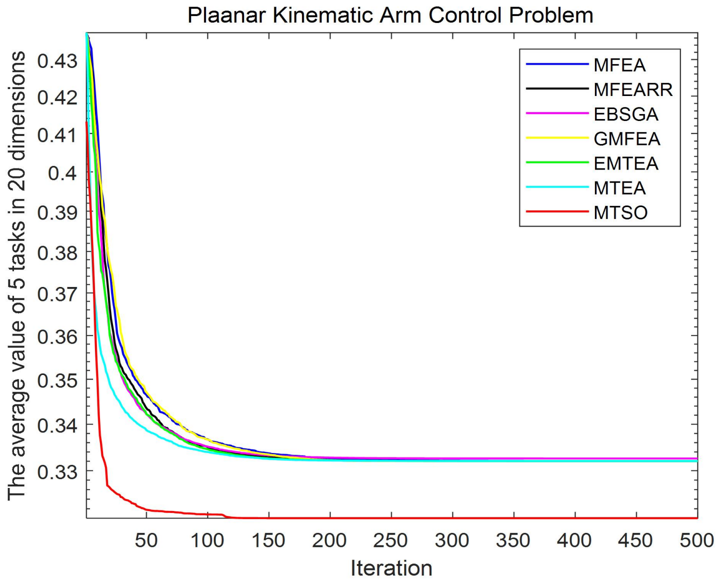

(a)

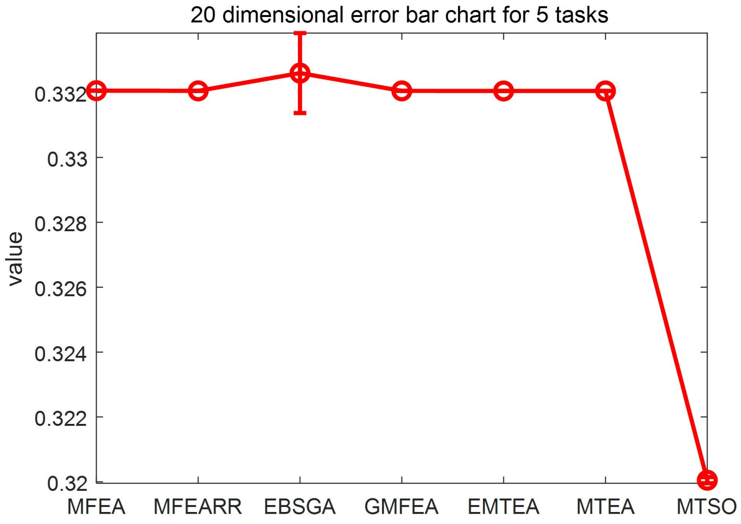

(b)

Supplemental Figure S10 Average convergence curve (a) and error bar(b) for the PKACP with 10 tasks in 5 dimensions

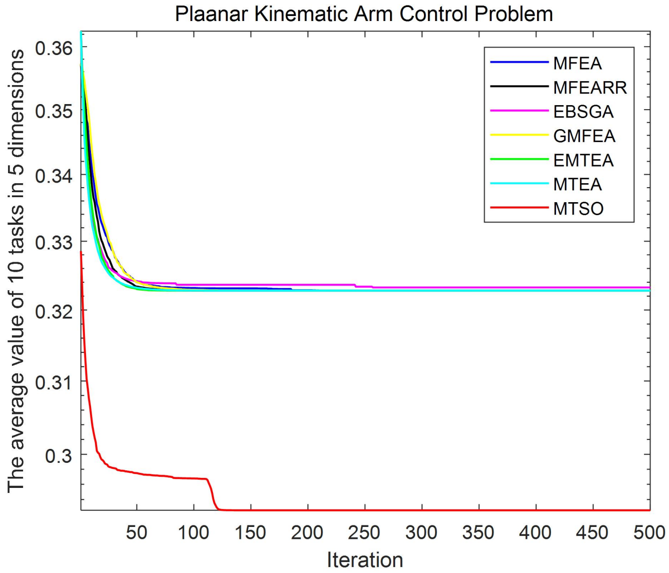

(a)

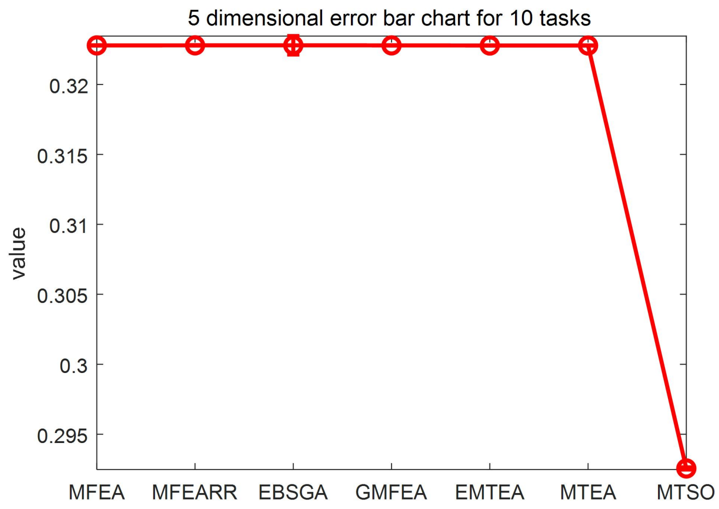

(b)

Supplemental Figure S11 Average convergence curve (a) and error bar(b) for the PKACP with 10 tasks in 10 dimensions

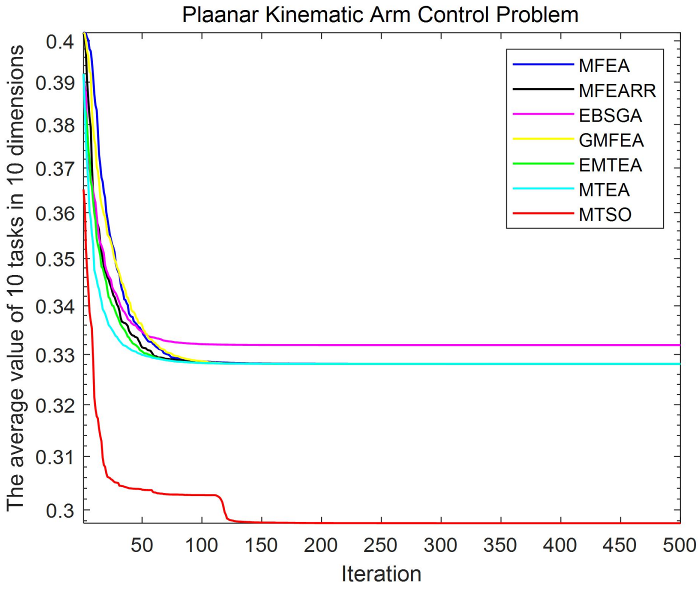

(a)

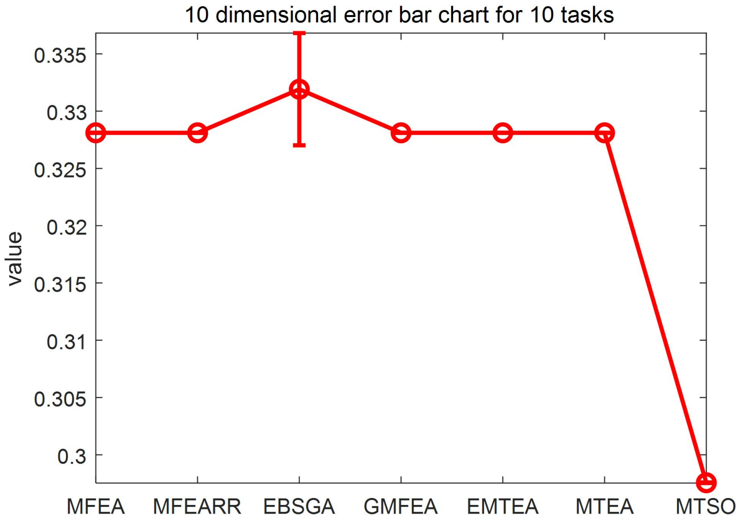

(b)

Supplemental Figure S12 Average convergence curve (a) and error bar(b) for the PKACP with 10 tasks in 20 dimensions

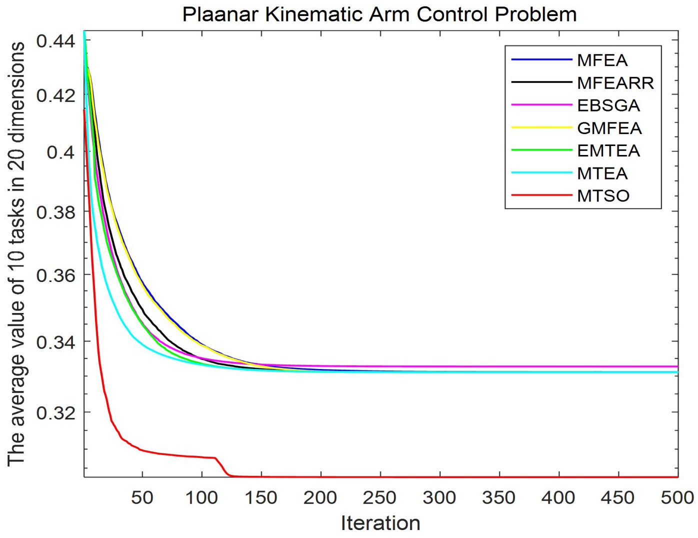

(a)

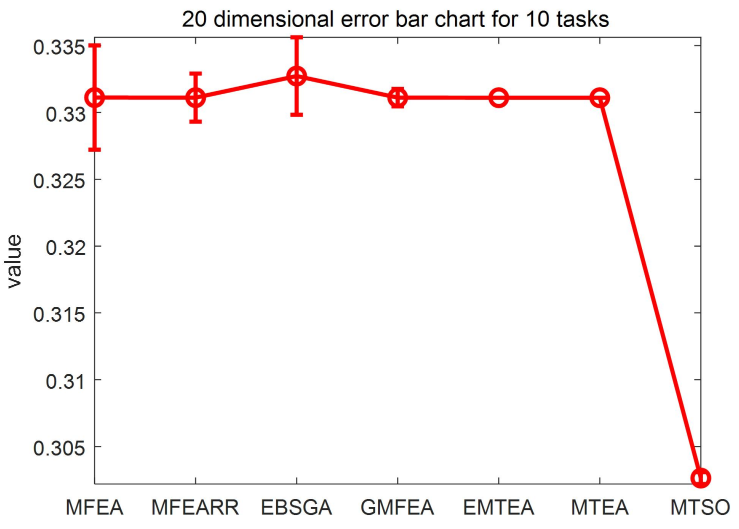

(b)

Supplemental Figure S13 Average convergence curve(a) and error bar(b) for the robot gripper design problem

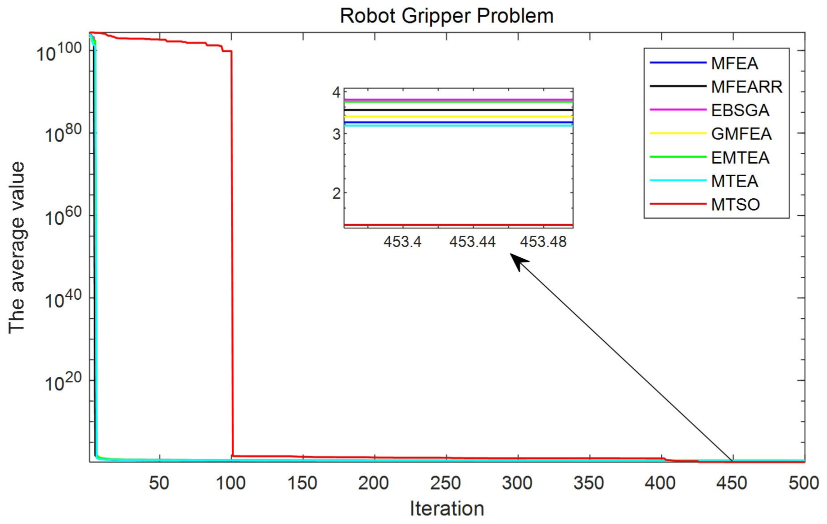

(a)

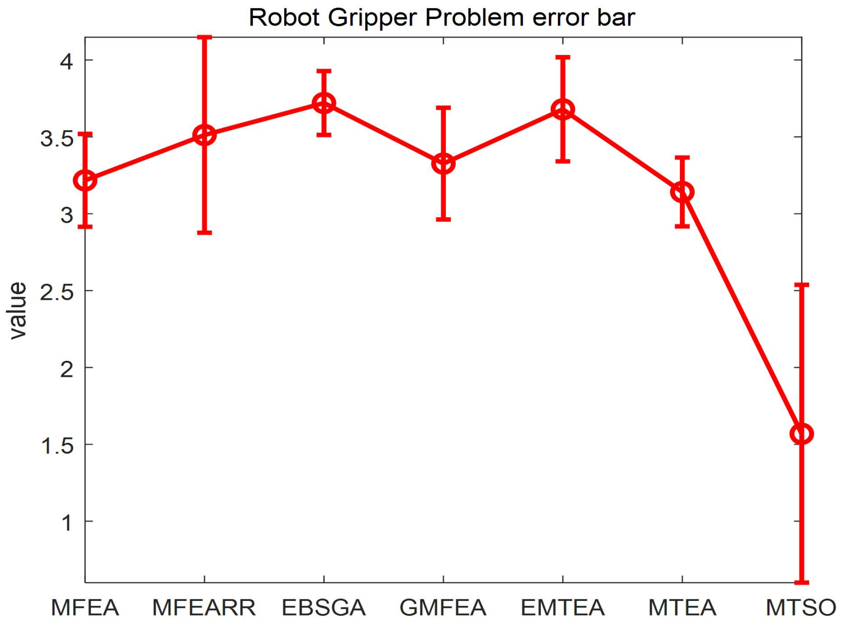

(b)

Supplemental Figure S14 Average convergence curve(a) and error bar(b) for the car side impact design problem

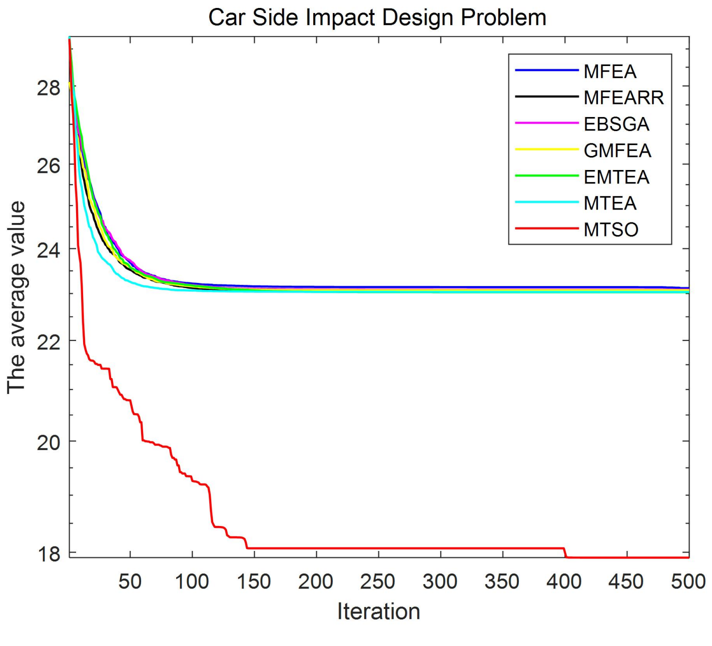

(a)

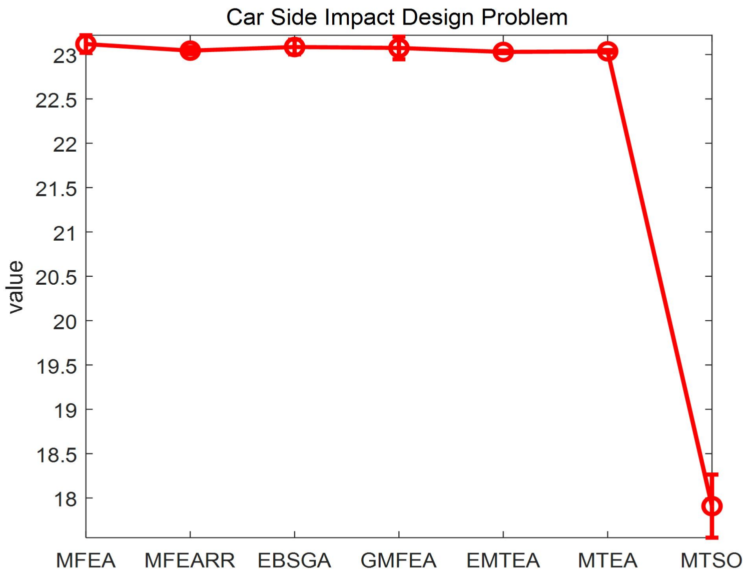
Car Side Impact Design Problem
value
23
22.5
22
21.5
21
20.5
20
19.5
19
18.5
18
MFEA
MFEARR
EBSGA
GMFEA
EMTEA
MTEA
MTSO

(b)
