# Supplementary material for: Multi-task snake optimization algorithm for global optimization and planar kinematic arm control problem
Source: PeerJ Comput Sci. 2025 Feb 11;11:e2688. doi: 10.7717/peerj-cs.2688 (PMC11888922; doi:10.7717/peerj-cs.2688)
Supplement: Supplemental Information 28 [file peerj-cs-11-2688-s028.pdf]

Supplemental Table S1 Abbreviations of professional nomenclature and their meanings

Symbol	Meaning
MTO	The Multitask Optimization
MTSO	Multitask Snake Optimization Algorithm
SO	Snake Optimization Algorithm
SI	swarm intelligence
KT	Knowledge transfer
MFEA	Multi-Factor Evolutionary Algorithm
Std	The standard deviation
PKACP	Planar Kinematic Arm Control Problem

Supplemental Table S2 The pseudo code for the MTSO

Algorithm 2 Multitask Snake Optimization Algorithm

Set Algorithm Parameters (Tasks,*N*, *T*,RMP,R1,nRepeat,ntasks)

Population initialization

While ($t \leq T_{max}$) **do**

    Using the Snake Optimization Algorithm to individually optimize and solve n tasks.

    Selecting the top fifth of individuals for each task as elite individuals.

  While (k≤no_of_tasks) **do**

    Generate random numbers r1 and r2.

    If (*r1<RMP*) **then**

      If (*r2<R1*) **then**

        Randomly transferring knowledge from the elite repository to non-task-specific individuals.

      Else

        Random perturbation applied to the worst-performing individuals in this task.

      End If

    Else

        Reverse learning through lens imaging applied to all individuals in this task.

    End If

  End While

End While

Return to the best solution.

Supplemental Table S3 Benchmark Test Functions for Multitask Optimization

Number	Name	Global minimum	Dimension	Function Characteristics
1	Griewank	0	30	Multi-modal
2	Rastrigin	0	30	Multi-modal
3	Ackley	0	30	Multi-modal
4	Schwefel	0	30	Multi-modal
5	Sphere	0	30	Multi-modal
6	Weierstrass	0	30	Multi-modal
7	Rosenbrock	0	30	Multi-modal

Supplemental Table S4 Results of Benchmark Function Testing

**Test Combinations**			**SAMTO**	**MFEA**	**MFEARR**	**LDAMFEA**	**MFEALBS**	**EBSGA**	**GMFEA**	**EMTEA**	**MTEA**	**MTSO**
		Mean	0.6312e-03	1.0477	0.9926	0.8321	1.0514	0.9961	1.0323	0.8860	0.3877	**0**
	Task1	Std	0.5018e-03	0.0160	0.0527	0.3142	0.0212	0.0653	0.0276	0.1047	0.1400	**0**
1		P-value	1.8214e-07	8.0065e-09	8.0061e-09	8.0065e-09	8.0065e-09	8.0063e-09	8.0065e-09	8.0065e-09	8.0065e-09	**-**
		Mean	0.2372e-03	10.0278	26.5813	5.2459	11.6189	23.9840	2.1528	14.9788	13.3953	**0**
	Task2	Std	0.2068e-03	7.5510	6.0821	7.0724	7.4337	10.3268	0.7868	3.7980	3.2456	**0**
		P-value	1.8213e-07	8.0065e-09	8.0065e-09	8.0062e-09	8.0065e-09	8.0065e-09	8.0065e-09	2.1518e-05	8.0065e-09	**-**
	run time		48.1177	27.6400	31.8536	43.9701	16.0716	349.477	17.4739	15.8550	16.9278	**10.9489**
		Mean	0.0106	1.2840	0.6417	2.5068	1.2674	0.6704	1.1626	0.6365	0.1848	**0**
	Task1	Std	0.0051	0.3620	0.1626	0.6995	0.3921	0.3105	0.5437	0.3880	0.0737	**0**
2		P-value	1.8214e-07	8.0065e-09	8.0065e-09	8.0065e-09	8.0065e-09	8.0065e-09	8.0065e-09	8.0065e-09	8.0065e-09	**-**
		Mean	0.0011	14.3847	27.2652	11.5904	12.8151	24.6515	4.4426	14.1171	13.7643	**0**
	Task2	Std	0.0012	5.4700	7.4646	5.7654	7.6266	7.5733	5.2226	3.0000	3.2217	**0**
		P-value	1.8214e-07	8.0065e-09	8.0065e-09	8.0065e-09	8.0065e-09	8.0065e-09	8.0065e-09	2.1518e-05	8.0065e-09	**-**
	run time		45.0181	23.0929	28.6530	40.4025	16.2880	17.7763	27.3497	15.3660	17.7276	**10.4660**
		Mean	**0**	1.3	0.7	5.2	1.3	0.7	1.0	0.5	**0**	**0**
	Task1	Std	0.0144	0.3883	0.2806	1.7615	0.3247	0.3092	0.2580	0.1048	0.0203	**0**
3		P-value	1.8214e-07	8.0065e-09	8.0065e-09	8.0065e-09	8.0065e-09	8.0065e-09	8.0065e-09	8.0065e-09	8.0065e-09	**-**
		Mean	1.4979e+03	1.7993e+03	1.7172e+03	3.6059e+03	1.8113e+03	1.8374e+03	**1.4147e+03**	1.8591e+03	1.7998e+03	4.3995e+03
	Task2	Std	220.3479	341.6261	385.8081	507.8107	302.3329	363.4312	296.8111	309.6063	**208.9724**	919.8908
		P-value	1.2009e-05	6.7956e-08	6.7956e-08	0.0043	6.7956e-08	6.7956e-08	6.7956e-08	0.1167	6.7956e-08	-
	run time		44.9512	27.6286	31.8203	43.8813	16.1299	17.5403	26.8410	15.8994	17.0137	**11.0284**
		Mean	0.1989e-03	7.7363	27.2852	0.0296	10.9225	19.4656	2.0076	16.0976	12.6099	**0**
	Task1	Std	0.1659e-03	6.0853	8.7128	0.0164	7.0862	10.0420	0.7292	6.7000	4.3864	**0**

4		P-value	1.8214e-07	8.0065e-09	8.0065e-09	8.0065e-09	8.0065e-09	8.0065e-09	8.0065e-09	8.0065e-09	8.0065e-09	**-**
	Task2	Mean	0.0010	0.0144	0.0070	0.0001	0.0160	0.0070	0.0101	0.0036	0.0009	**0**
		Std	0.0008e-03	0.0041	0.0016	0.0001	0.0048	0.0024	0.0035	0.0013	0.0006	**0**
		P-value	1.8214e-07	8.0065e-09	8.0065e-09	8.0065e-09	8.0065e-09	8.0065e-09	8.0065e-09	2.1518e-05	8.0065e-09	-
	run time		44.5827	24.3489	29.5979	43.5837	16.1211	17.4975	26.8535	15.8589	16.9460	**10.8286**
5	Task1	Mean	0.0471	1.1535	0.7104	4.3679	1.1913	0.6138	1.1285	0.4474	0.0013	**0**
		Std	0.0177	0.3503	0.2974	0.7589	0.3988	0.2728	0.2929	0.1884	0.0013	**0**
		P-value	1.8214e-07	8.0065e-09	8.0065e-09	8.0065e-09	8.0065e-09	8.0065e-09	8.0065e-09	8.0065e-09	8.0065e-09	**-**
	Task2	Mean	69.5086	98.2676	104.0581	33.4881	116.9216	86.0457	93.4695	92.6134	43.7678	**28.7488**
		Std	30.2752	42.1083	42.8453	24.4403	60.7051	39.8790	25.1838	30.4625	29.3195	**3.4728**
		P-value	4.6958e-04	1.0646e-07	1.0646e-07	0.5792	1.8074e-05	1.8030e-06	1.2009e-06	0.1167	0.6554	-
	run time		45.1073	23.9711	28.4164	41.3712	15.3974	16.1541	26.8961	16.3333	18.0274	**12.0562**
6	Task1	Mean	0.0078	0.3541	0.3181	0.1078	0.3247	0.6721	0.3863	0.3238	0.0006	**0**
		Std	0.0034	0.0840	0.0768	0.3140	0.0809	0.3398	0.1253	0.0999	0.0006	**0**
		P-value	1.8214e-07	8.0065e-09	8.0065e-09	8.0065e-09	8.0065e-09	8.0065e-09	8.0065e-09	8.0065e-09	8.0065e-09	**-**
	Task2	Mean	0.1359	1.4264	1.3755	0.4663	1.3878	1.2296	1.4394	0.9007	0.1383	**0**
		Std	0.0305	0.1844	0.2186	0.2159	0.1588	0.1791	0.2069	0.1496	0.0690	**0**
		P-value	1.8214e-07	8.0065e-09	8.0065e-09	8.0065e-09	8.0065e-09	8.0065e-09	8.0065e-09	2.1518e-05	8.0065e-09	-
	run time		51.4328	36.4051	44.0726	53.4250	29.0845	30.0345	38.1124	27.8354	28.9654	**25.0328**
7	Task1	Mean	0.2599	15.8928	23.9193	33.8001	17.2246	24.2140	14.7936	15.9240	11.6804	**0**
		Std	0.1448	3.1351	5.0561	11.1391	3.1973	5.8462	3.1364	4.1156	3.6569	**0**
		P-value	1.8214e-07	8.0065e-09	8.0065e-09	8.0065e-09	8.0065e-09	8.0065e-09	8.0065e-09	8.0065e-09	8.0065e-09	**-**
	Task2	Mean	72.0445	97.4159	100.7158	**29.8540**	97.9908	97.3434	113.2386	97.8188	51.5326	32.1433
		Std	35.4370	35.6230	28.5418	**8.6396**	43.5270	46.6113	57.4444	31.4901	35.3664	16.6833
		P-value	0.0014	4.539e-07	4.539e-07	0.0639	4.539e-07	3.4156e-07	2.9598e-07	0.1604	0.7150	-
	run time		45.0336	24.2641	29.4173	43.3907	16.1454	17.4576	26.7884	15.8866	16.9636	**10.9071**

8	Task1	Mean	0.0165	0.7770	0.7439	0.1704	0.7694	0.9463	0.8054	0.8230	0.0040	**0**
		Std	0.0157	0.1376	0.1344	0.1715	0.1453	0.1303	0.1200	0.0994	0.0057	**0**
		P-value	1.8214e-07	8.0065e-09	8.0065e-09	8.0065e-09	8.0065e-09	8.0065e-09	8.0065e-09	8.0065e-09	8.0065e-09	**-**
	Task2	Mean	0.2681	1.5554	1.5301	0.7688	1.5719	1.2065	1.5856	0.8684	0.1271	**0**
		Std	0.1142	0.1963	0.1816	0.4091	0.1867	0.1956	0.1693	0.1212	0.0454	**0**
		P-value	1.8214e-07	8.0065e-09	8.0065e-09	8.0065e-09	8.0065e-09	8.0065e-09	8.0065e-09	2.1518e-05	8.0065e-09	**-**
	run time		52.6870	35.6631	41.3456	52.3301	28.4588	30.6134	41.3324	28.6141	30.7128	**26.4293**
9	Task1	Mean	0.2	16.5	23.3	97.7	15.3	26.2	16.8	19.9	25.5	**0**
		Std	0.0901	3.1174	6.6179	20.4052	2.1104	7.1304	3.3623	5.0259	7.2174	**0**
		P-value	1.8214e-07	8.0065e-09	8.0065e-09	8.0065e-09	8.0065e-09	8.0065e-09	8.0065e-09	8.0065e-09	8.0065e-09	**-**
	Task2	Mean	1.6982e+03	1.9133e+03	1.7811e+03	3.5338e+03	1.9642e+03	1.7361e+03	**1.6711e+03**	1.8715e+03	1.7356e+03	4.5326e+03
		Std	279.4933	**237.3464**	290.2974	573.9668	285.3062	321.4278	318.1099	371.4803	386.4274	656.4530
		P-value	1.2009e-05	6.7956e-08	6.7956e-08	5.2550e-05	6.7956e-08	6.7956e-08	6.7956e-08	0.1167	6.7956e-08	-
	run time		39.9333	19.4119	23.7505	34.6596	13.0308	14.1564	21.6337	12.8438	13.6655	**9.0428**

Supplemental Table S5 Results of Noisy Multitask Benchmark Function Testing

Test Combinations			SAMTO	MFEA	MFEARR	LDAMFEA	MFEALBS	EBSGA	GMFEA	EMTEA	MTEA	MTSO
1	Task1	Mean	10.0007	11.0522	11.0208	10.8458	11.0492	11.0108	11.0222	10.9140	10.3307	**10**
		Std	0.0004	0.0339	0.0121	0.2468	0.0211	0.0175	0.0389	0.0958	0.0815	**0**
		p-value	6.3864e-05	6.3864e-05	6.3864e-05	6.3864e-05	6.3864e-05	6.3864e-05	6.3864e-05	6.3864e-05	6.3864e-05	**-**
	Task2	Mean	10.0003	22.1661	33.1851	12.3083	21.7948	31.4878	12.0631	22.9343	23.6540	**10**
		Std	0.0002	8.0770	3.7587	3.3575	6.8213	9.7010	0.6762	4.8967	3.7461	**0**
		p-value	6.3864e-05	6.3864e-05	6.3864e-05	6.3864e-05	6.3864e-05	6.3864e-05	6.3864e-05	0.1818	6.3864e-05	**-**
	run time		48.475	23.4591	28.7640	42.5013	15.7559	17.1184	26.1091	15.4868	16.5772	**9.833**
		Mean	10.0105	11.1892	10.6775	12.4526	11.2583	10.6488	11.0016	10.4852	10.1982	**10**

	Task1	Std	0.0042	0.3905	0.2930	0.5516	0.3409	0.2983	0.3444	0.2933	0.0672	**0**
		p-value	6.3864e-05	6.3864e-05	6.3864e-05	6.3864e-05	6.3864e-05	6.3864e-05	6.3864e-05	6.3864e-05	6.3864e-05	-
2		Mean	10.0011	22.9403	39.0216	22.6929	23.8359	30.3381	13.1910	24.6026	22.3765	**10**
	Task2	Std	0.0009	6.4943	8.1703	4.6181	5.3002	8.2905	3.2688	2.8066	4.0868	**0**
		p-value	6.3864e-05	6.3864e-05	6.3864e-05	6.3864e-05	6.3864e-05	6.3864e-05	6.3864e-05	0.1818	6.3864e-05	-
	run time		41.286	21.6697	27.0815	38.2364	15.0453	16.8033	25.8573	14.7800	15.4621	**9.737**
		Mean	**10**	11.3	10.6	14.8	11.2	10.7	11.0	10.5	**10**	**10**
	Task1	Std	0.0145	0.3606	0.1995	1.2186	0.4533	0.4650	0.2180	0.1080	0.0302	**0**
		p-value	6.3864e-05	6.3864e-05	6.3864e-05	6.3864e-05	6.3864e-05	0.1818	6.3864e-05	6.3864e-05	6.3864e-05	-
3		Mean	**1.5368e+03**	1.9820e+03	1.6631e+03	3.4482e+03	2.0063e+03	1.6933e+03	1.8636e+03	1.6527e+03	1.8315e+03	3.9555e+03
	Task2	Std	**250.2424**	477.9580	411.1307	543.3261	367.2894	283.8110	283.0637	348.3263	356.5711	919.8908
		p-value	1.8267e-04	1.8267e-04	1.8267e-04	0.4274	1.8267e-04	1.8267e-04	1.8267e-04	0.1818	1.8267e-04	-
	run time		44.325	22.6617	27.9563	41.7159	15.4353	16.5753	25.2840	15.0218	16.1239	**10.047**
		Mean	10.0003	18.4632	37.0405	10.0302	23.3919	30.1704	12.4131	22.2392	21.0861	**10**
	Task1	Std	0.0002	7.2876	3.0776	0.0290	5.8891	6.8777	0.4754	3.3141	4.7030	**0**
		p-value	6.3864e-05	6.3864e-05	6.3864e-05	6.3864e-05	6.3864e-05	6.3864e-05	6.3864e-05	6.3864e-05	6.3864e-05	-
4		Mean	**10**	10.0160	10.0088	10.0002	10.0163	10.0083	10.0122	10.0042	10.0007	**10**
	Task2	Std	**0**	0.0042	0.0028	0.0002	0.0047	0.0021	0.0024	0.0021	0.0004	**0**
		p-value	6.3864e-05	6.3864e-05	6.3864e-05	6.3864e-05	6.3864e-05	6.3864e-05	6.3864e-05	0.1818	6.3864e-05	-
	run time		42.089	23.3309	29.5363	40.8091	15.5381	16.9854	26.0091	15.2488	13.7762	**7.874**
		Mean	10.0417	11.1647	10.5103	13.1881	11.0718	10.6097	11.1838	10.3701	10.0037	**10**
	Task1	Std	0.0114	0.4749	0.1204	0.8932	0.2239	0.2014	0.3759	0.1100	0.0045	**0**
		p-value	6.3864e-05	6.3864e-05	6.3864e-05	6.3864e-05	6.3864e-05	6.3864e-05	6.3864e-05	6.3864e-05	6.3864e-05	-
5		Mean	83.7798	95.8520	86.9020	43.3304	115.1157	132.2997	139.1000	99.8994	79.4019	**38.2876**
	Task2	Std	21.8488	45.6634	35.2674	17.2054	35.1739	72.8975	50.5977	34.7151	31.9576	**0.8658**
		p-value	0.0028	0.0211	0.0173	0.9097	1.8267e-04	1.8267e-04	1.8267e-04	0.1818	0.1041	-

	run time		43.221	22.7892	27.5848	38.7720	15.1517	16.4239	25.1570	14.6352	15.7326	**9.771**
6	Task1	Mean	10.0074	10.3819	10.3520	10.1715	10.3807	10.7010	10.4145	10.3045	10.0004	**10**
		Std	0.0024	0.0783	0.0753	0.4254	0.1143	0.4480	0.0931	0.0622	0.0004	**0**
		p-value	6.3864e-05	6.3864e-05	6.3864e-05	6.3864e-05	6.3864e-05	6.3864e-05	6.3864e-05	6.3864e-05	6.3864e-05	**-**
	Task2	Mean	10.1419	11.4734	11.4259	10.6034	11.4504	11.2005	11.5628	11.9000	10.1514	**10**
		Std	0.0289	0.1589	0.1251	0.5724	0.2401	0.1657	0.1935	0.1366	0.0630	**0**
		p-value	6.3864e-05	6.3864e-05	6.3864e-05	6.3864e-05	6.3864e-05	6.3864e-05	6.3864e-05	0.1818	6.3864e-05	**-**
	run time		52.837	35.9140	42.8797	51.6958	27.8508	28.8004	37.7383	27.4866	28.8540	**24.051**
7	Task1	Mean	10.2587	25.6990	35.4189	40.1412	26.7818	32.7076	28.3435	27.8744	23.4709	**10**
		Std	0.1060	2.3959	7.8990	7.3986	3.4329	8.8554	3.6908	2.8039	2.8258	**0**
		p-value	6.3864e-05	6.3864e-05	6.3864e-05	6.3864e-05	6.3864e-05	6.3864e-05	6.3864e-05	6.3864e-05	6.3864e-05	**-**
	Task2	Mean	94.6885	104.5505	108.8248	**37.9496**	113.0444	88.6591	111.7771	84.4852	75.2232	38.2599
		Std	35.8293	29.1413	40.6339	1.0084	38.0231	30.1330	38.7332	34.3627	37.0461	**0.6885**
		p-value	0.0028	1.8267e-04	1.8267e-04	0.1859	1.8267e-04	1.8267e-04	1.8267e-04	0.1818	0.2413	**-**
	run time		46.398	22.2308	27.3875	40.1320	15.7565	16.5845	25.0912	14.9420	15.7830	**9.759**
8	Task1	Mean	10.0225	10.7407	10.7442	10.1375	10.7258	10.9946	10.8173	10.7922	10.0092	**10**
		Std	0.0178	0.1917	0.1061	0.0973	0.1596	0.0457	0.1368	0.1509	0.0132	**0**
		p-value	6.3864e-05	6.3864e-05	6.3864e-05	6.3864e-05	6.3864e-05	6.3864e-05	6.3864e-05	6.3864e-05	6.3864e-05	**-**
	Task2	Mean	10.2910	11.5105	11.4987	10.6690	11.4624	11.1831	11.5750	10.8799	10.1102	**10**
		Std	0.1073	0.2175	0.1142	0.2401	0.1721	0.1770	0.1614	0.0991	0.0925	**0**
		p-value	6.3864e-05	6.3864e-05	6.3864e-05	6.3864e-05	6.3864e-05	6.3864e-05	6.3864e-05	0.1818	6.3864e-05	**-**
	run time		50.113	34.4686	39.5348	49.8755	27.3690	28.7603	39.2262	27.2688	28.0101	**23.382**
9	Task1	Mean	10.1	25.9	31.5	99.6	25.1	34.6	27.2	31.2	34.1	**10**
		Std	0.0568	3.3173	4.0681	23.1852	3.0272	5.5083	3.0711	2.0975	6.4926	**0**
		p-value	6.3864e-05	6.3864e-05	6.3864e-05	6.3864e-05	6.3864e-05	6.3864e-05	6.3864e-05	6.3864e-05	6.3864e-05	**-**
		Mean	**1.6767e+03**	2.0877e+03	2.0293e+03	3.3335e+03	1.8214e+03	1.7965e+03	1.8089e+03	1.8708e+03	1.9523e+03	4.4536e+03

Task2	Std	**201.3052**	295.7972	491.0466	623.4152	392.8547	280.1191	271.9370	279.0046	316.8564	607.6476
	p-value	1.8267e-04	1.8267e-04	1.8267e-04	0.0036	1.8267e-04	1.8267e-04	1.8267e-04	0.1818	1.8267e-04	-
run time		39.668	18.9909	23.0260	33.5925	12.6675	13.6826	20.8503	12.3916	13.1330	**7.978**

Supplemental Table S6 Average Ranking from Friedman Test of MTSO with Different Population Sizes on Benchmark Test Functions

	**Population size**	**30**	**50**	**100**
1	Task1 average rank	2.30	1.95	1.75
	Task2 average rank	2	2	2
2	Task1 average rank	2	2	2
	Task2 average rank	2.45	1.80	1.75
3	Task1 average rank	2	2	2
	Task2 average rank	2.45	1.85	1.70
4	Task1 average rank	2	2	2
	Task2 average rank	2	2	2
5	Task1 average rank	2	2	2
	Task2 average rank	2.30	1.90	1.80
6	Task1 average rank	2.15	2	1.85
	Task2 average rank	2	2	2
7	Task1 average rank	2	2	2
	Task2 average rank	2.15	2	1.85
8	Task1 average rank	2	2	2
	Task2 average rank	2	2	2
9	Task1 average rank	2	2	2
	Task2 average rank	2.20	1.95	1.85
	Total ranking	38	35.45	34.55
	Rank	3	2	1

Supplemental Table S7 Friedman average rankings of MTSO on benchmark test functions under different parameter settings.

	parameter	RMP = 0.9, R1 = 0.95	RMP = 0.9, R1 = 0.85	RMP = 0.9, R1 = 0.75	RMP = 0.7, R1 = 0.95	RMP = 0.7, R1 = 0.85	RMP = 0.7, R1 = 0.75	RMP = 0.5, R1 = 0.95	RMP = 0.5, R1 = 0.85	RMP = 0.5, R1 = 0.75	RMP = 0.3, R1 = 0.95	RMP = 0.3, R1 = 0.85	RMP = 0.3, R1 = 0.75
1	Task1	6.5	6.5	6.5	6.5	6.5	6.5	6.5	6.5	6.5	6.5	6.5	6.5
	Task2	6.5	6.5	6.5	6.5	6.5	6.5	6.5	6.5	6.5	6.5	6.5	6.5
2	Task1	6.5	6.5	6.5	6.5	6.5	6.5	6.5	6.5	6.5	6.5	6.5	6.5
	Task2	6.5	6.5	6.5	6.5	6.5	6.5	6.5	6.5	6.5	6.5	6.5	6.5
3	Task1	6.5	6.5	6.5	6.5	6.5	6.5	6.5	6.5	6.5	6.5	6.5	6.5
	Task2	7.8	6.4	5.3	4.9	5.3	6.1	5.0	6.1	7.2	9.4	6.5	8.0
4	Task1	6.5	6.5	6.5	6.5	6.5	6.5	6.5	6.5	6.5	6.5	6.5	6.5
	Task2	6.5	6.5	6.5	6.5	6.5	6.5	6.5	6.5	6.5	6.5	6.5	6.5
5	Task1	6.5	6.5	6.5	6.5	6.5	6.5	6.5	6.5	6.5	6.5	6.5	6.5
	Task2	9.0	6.7	6.2	6.9	6.1	7.0	5.1	7.6	5.8	6.0	4.7	6.9
6	Task1	6.5	6.5	6.5	6.5	6.5	6.5	6.5	6.5	6.5	6.5	6.5	6.5
	Task2	6.5	6.5	6.5	6.5	6.5	6.5	6.5	6.5	6.5	6.5	6.5	6.5
7	Task1	6.5	6.5	6.5	6.5	6.5	6.5	6.5	6.5	6.5	6.5	6.5	6.5
	Task2	7.1	7.2	7.8	7.4	5.8	7.4	6.2	7.4	5.5	5.2	4.8	6.2
8	Task1	6.5	6.5	6.5	6.5	6.5	6.5	6.5	6.5	6.5	6.5	6.5	6.5
	Task2	6.5	6.5	6.5	6.5	6.5	6.5	6.5	6.5	6.5	6.5	6.5	6.5
9	Task1	6.5	6.5	6.5	6.5	6.5	6.5	6.5	6.5	6.5	6.5	6.5	6.5
	Task2	5.4	5.5	6.2	6.7	5.5	8.4	5.6	7.1	6.9	7.1	6.3	7.3
	Total ranking	120.3	116.8	116.5	116.9	113.7	119.9	112.9	119.2	116.4	118.5	113.1	119.2
	Rank	11	6	5	7	3	10	1	9	4	8	2	9

Supplemental Table S8 Friedman Average Rankings of Experimental Results on Benchmark Test Functions for Different Knowledge Transfer Scales

**Num**	**Population size**	**1/10*N**	**1/5*N**	**1/2*N**
1	Task1 average rank	2	2	2
	Task2 average rank	2	2	2
2	Task1 average rank	2	2	2
	Task2 average rank	2	2	2
3	Task1 average rank	2	2	2
	Task2 average rank	2	1.4	2.6
4	Task1 average rank	2	2	2
	Task2 average rank	2	2	2
5	Task1 average rank	2	2	2
	Task2 average rank	1.6	2	2.4
6	Task1 average rank	2	2	2
	Task2 average rank	2	2	2
7	Task1 average rank	2	2	2
	Task2 average rank	1.7	2.1	2.2
8	Task1 average rank	2	2	2
	Task2 average rank	2	2	2
9	Task1 average rank	2	2	2
	Task2 average rank	2.5	2	1.5
	Total ranking	35.8	35.5	36.7
	Rank	2	1	3

Supplemental Table S9 Knowledge Utilization Rate of MTSO on Benchmark Test Functions

**Number**	**Task**	**Knowledge utilization rate**	**Task**	**Knowledge utilization rate**
1	Task1	0.3214	Task2	0.2731
2	Task1	0.3506	Task2	0.2866
3	Task1	0.3029	Task2	0.5500
4	Task1	0.2462	Task2	0.4296
5	Task1	0.3350	Task2	0.6253
6	Task1	0.3951	Task2	0.2001
7	Task1	0.1324	Task2	0.6745
8	Task1	0.3098	Task2	0.1982
9	Task1	0.1473	Task2	0.4723

Supplemental Table S10 The PKACP experimental result of 6 experimental groups.

	Algorithm Name	Mean	Std	Run time
5 tasks in 5 dimensions	MTSO	**0.3076**	1.7022e-05	**8.8439**
	MFEA	0.3211	2.0709e-05	17.6964
	MFEARR	0.3210	7.3031e-09	22.4309
	EBSGA	0.3230	0.0045	34.2621
	GMFEA	0.3210	1.3364e-06	19.0942
	EMTEA	0.3211	**2.6471e-10**	31.4896
	MTEA	0.3210	7.2178e-09	35.7847
5 tasks in 10 dimensions	MTSO	**0.3158**	4.9406e-05	**11.7468**
	MFEA	0.3284	1.9342e-06	18.6173
	MFEARR	0.3283	1.0711e-07	23.7195
	EBSGA	0.3288	0.0012	36.6925
	GMFEA	0.3283	1.4112e-06	20.1048
	EMTEA	0.3283	**4.9627e-08**	34.0945
	MTEA	0.3283	6.8272e-08	37.6432
5 tasks in 20 dimensions	MTSO	**0.3201**	9.4061e-05	**17.5135**
	MFEA	0.3322	4.2842e-06	19.3467
	MFEARR	0.3321	1.7749e-06	24.3141
	EBSGA	0.3326	0.0012	38.5810
	GMFEA	0.3322	8.0129e-07	20.7831
	EMTEA	0.3321	**1.4033e-07**	35.9231
	MTEA	0.3321	2.8983e-07	39.5354
10 tasks in 5 dimensions	MTSO	**0.2926**	7.9567e-05	30.7927
	MFEA	0.3228	6.6682e-06	**28.5592**
	MFEARR	0.3228	1.2774e-05	34.7434
	EBSGA	0.3232	6.5676e-04	82.7234
	GMFEA	0.3228	5.2002e-06	30.8124
	EMTEA	0.3228	5.2904e-09	75.6532
	MTEA	0.3228	**3.0057e-09**	75.5142
10 tasks in 10 dimensions	MTSO	**0.2976**	2.6395e-05	49.6888
	MFEA	0.3281	1.5316e-06	**30.9493**
	MFEARR	0.3281	4.8060e-07	37.2470
	EBSGA	0.3319	0.0049	92.7837
	GMFEA	0.3281	1.1479e-07	33.6735
	EMTEA	0.3281	**2.7757e-08**	98.2108
	MTEA	0.3281	3.6312e-08	100.3812
10 tasks in 20 dimensions	MTSO	**0.3026**	4.4554e-04	66.9099
	MFEA	0.3315	0.0039	**31.1880**
	MFEARR	0.3313	0.0018	36.7254
	EBSGA	0.3327	0.0029	100.485
	GMFEA	0.3314	6.5085e-04	33.5761
	EMTEA	0.3313	**2.0622e-06**	94.9603
	MTEA	0.3313	2.0644e-06	98.5762

Supplemental Table S11 The statistical results of MTSO and other comparison algorithms on the two-task robot gripper design problem.

Algorithm Name	Mean	Std	Run time(s)
MTSO	**1.5689**	0.9686	**86.9822**
MFEA	3.2169	0.3019	127.2030
MFEARR	3.5121	0.6358	103.0602
EBSGA	3.7202	**0.2078**	90.6607
GMFEA	3.3260	0.3632	120.1103
EMTEA	3.6792	0.3385	128.9005
MTEA	3.1415	0.2240	122.9216

Supplemental Table S12 The statistical results of MTSO and other comparison algorithms on the two-task car side impact design problem.

Algorithm Name	Mean	Std	Run time(s)
MTSO	**17.9085**	0.1558	**4.3714**
MFEA	23.1184	0.0988	9.9254
MFEARR	23.0441	0.0398	12.3935
EBSGA	23.0849	0.0826	6.8581
GMFEA	23.0744	0.1276	11.1446
EMTEA	23.0296	**0.0138**	6.1104
MTEA	23.0364	0.0145	6.5282
